# Palladium(II)/Norbornene Cooperative Catalysis for Regioselective C–H Silylation Toward Diversified Silylindoles

**DOI:** 10.3390/molecules31152724

**Published:** 2026-08-05

**Authors:** Wenguang Li, Xinfeng Cheng, Qi Suo, Ke Hu, Yuqing Wang, Jianguang Zhu, Yongqi Yu

**Affiliations:** 1 College of Chemistry and Pharmaceutical Engineering, Nanyang Normal University, Nanyang 473061, China; 2 Key Laboratory of Research on Effective Substances and Quality Control of Traditional Chinese Medicine, Nanyang Medical College, Nanyang 473061, China; 3 School of Pharmacy, Henan University of Chinese Medicine, Zhengzhou 450046, China

**Keywords:** C–H silylation, Catellani reaction, palladacycle

## Abstract

A strategy for the selective C–H silylation of 7-phenyl-1*H*-indoles has been established via palladium(II)/norbornene cooperative catalysis. This method employs norbornene (NBE) as both a C–H activation mediator and an alkylating agent to access arylsilanes with exceptional selectivity. Kinetic isotope effect (KIE) studies revealed that C–H cleavage is the rate-determining step, consistent with a mechanism proceeding through an eight-membered palladacycle.

## 1. Introduction

Organosilicon compounds represent a class of highly significant molecules, prized for their distinct chemical and biological properties. Owing to this unique combination of features, they have found extensive applications across diverse fields, including synthetic chemistry [1], materials science [2], and pharmaceutical development [3]. In the past few years, transition-metal-catalyzed C−H silylation has proven to be a powerful strategy to access valuable silicon-containing molecules with high selectivity and efficiency [4]. Among them, the employment of hexamethyldisilane as a trimethylsilylating reagent in palladium-catalyzed direct C−H silylation has garnered considerable attention, owing to its efficacy in these transformations. However, the developed methods are predominantly limited to Pd(0)-catalyzed C–H silylation of aryl halides [5,6]. While effective, current Pd(II)-catalyzed C–H silylation strategies for arenes inherently depend on pre-installed directing groups [7,8,9,10], which considerably restricts their applicability and overall efficiency. Therefore, the development of new Pd(II)-catalyzed protocols for the selective C–H silylation of arenes without directing groups is highly valuable.

**Scheme 1:**
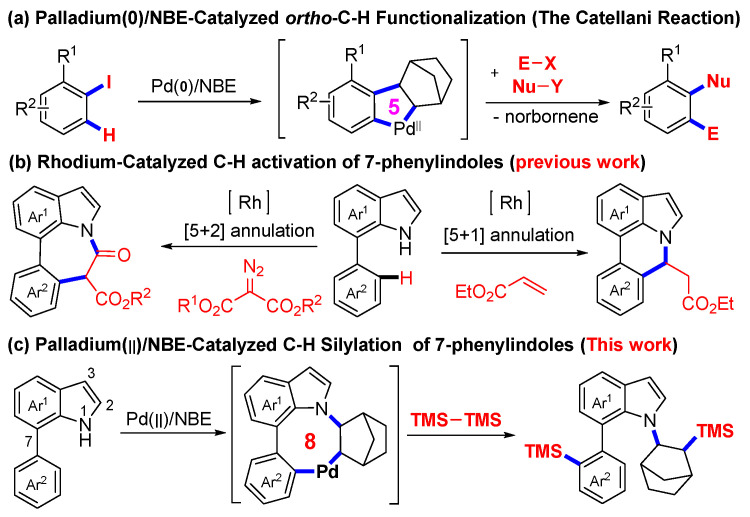
Transition-metal-catalyzed selective C–H functionalization.

Proceeding via a pivotal five-membered aryl-NBE palladacycle (**ANP**), the Catellani reaction has established itself as a highly valuable method for the synthesis of polysubstituted arenes and benzofused compounds. This transformation, first reported by Catellani et al. [11,12,13,14,15,16,17,18], permits the sequential functionalization of the *ortho*- and *ipso*-positions of aryl halides (Scheme 1a). Furthermore, it is well known that a wide range of nitrogen-based motifs, such as heterocycles, imines, and azo groups, have been widely employed as monodentate directing groups due to their strong coordination to various metals [19]. In contrast to the conventional Catellani reaction that employs a **C-ANP** species, these transformations skillfully leverage a more challenging polar **N-ANP** intermediate. Currently, Pd(II)/NBE-catalyzed *ortho*-C−H functionalization of free NH-indoles was realized by the Bach group [20] and others [21,22,23]. Recently, Huang and co-workers first reported the development of rhodium-catalyzed C−H activation of 7-phenyl-1*H*-indoles (Scheme 1b) [24]. Inspired by these valuable results, we directed our efforts toward the development of a palladium(II)/NBE-catalyzed C–H silylation reaction specifically for 7-phenyl-1*H*-indoles. Herein, we detail our results.

## 2. Results and Discussion

Our initial studies involved the intermolecular cascade reaction of 7-phenyl-1*H*-indole (**1a**) with norbornene (NBE) and hexamethyldisilane employing the conditions of Pd(OAc)_2_ (10 mol%), Cu(OAc)_2_ (3 equiv), and K_2_CO_3_ (2 equiv) in MeCN at 100 °C under an air atmosphere. This leads to the generation of disilylation product **2a** in 56% yield (entry 1). Under nitrogen atmosphere, **2a** was obtained in 43% yield (entry 2). Other catalysts such as Pd_2_(dba)_3_, PdCl_2_(PPh_3_)_2_, Pd(PPh_3_)_4_, and PdCl_2_ gave unsatisfactory results (entries 3−6). No reaction was observed without the addition of a palladium catalyst (entry 7). Replacing Cu(OAc)_2_ with other oxidants such as CuCl_2_, benzoquinone (BQ), and K_2_S_2_O_8_ failed to furnish the desired products, and starting material **1a** was recovered (entries 8−10). When other bases such as KOH, *^t^*BuOK, KOAc, Cs_2_CO_3_, and K_3_PO_4_ were employed, an obvious decrease in yield was observed across the board (entries 11−15). After an extensive screen of solvents, we found that the use of CH_3_CN gave the best result for **2a**, and other solvents such as 1,4-dioxane, toluene, and DMF led to almost complete reaction shutdown (entries 16−18). Next, we attempted to employ DABCO as an additive for the silylation reaction, and to our delight, the reaction of 7-phenyl-1*H*-indole **1a** with NBE and hexamethyldisilane afforded target product **2a** in an isolated yield of 75% (entry 19). Finally, deviation from the optimal temperature, either higher or lower, resulted in slightly reduced yield (entries 20−21). Moreover, compound **2a** was isolated in 70% yield from the reaction of **1a** on a 1 mmol scale. Accordingly, the optimal reaction conditions were shown in entry 19 of Table 1.

**Scheme 2:**
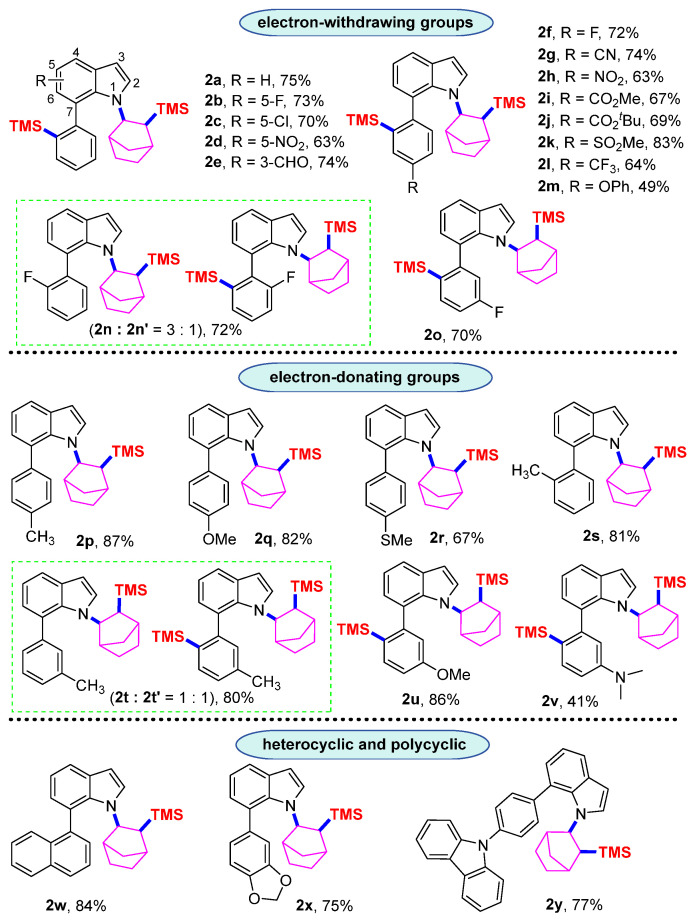
Substrate scope of 7-phenyl-1*H*-Indols in the presence of norbornene ^[a,b]^. ^[a]^ Reaction conditions: 7-phenyl-1*H*-indole **1a** (0.3 mmol, 1.0 equiv), NBE (0.6 mmol, 2 equiv), hexamethyldisilane (0.6 mmol, 2 equiv), Pd(OAc)_2_ (10 mol%), Cu(OAc)_2_ (3 equiv), K_2_CO_3_ (2 equiv), DABCO (1 equiv), and MeCN (3 mL) at 100 °C for 24 h under an air atmosphere. ^[b]^ Isolated yields.

Subsequently, our efforts were directed toward expanding the substrate scope of this methodology, utilizing the aforementioned optimal conditions (Scheme 2). Initially, a range of electron-withdrawing groups (for example, 5-F, 5-Cl, 5-NO_2_, and 3-CHO), on the phenyl of indole were examined, and the relevant products **2b**–**2e** were acquired in 63–74% yields. We were pleased to find that the electron-withdrawing groups (F, CN, NO_2_, CO_2_CH_3_, CO_2_*^t^*Bu, SO_2_Me, CF_3_, and OPh) at the *para* position on the 7-phenyl of 7-phenyl-1*H*-indole were also well tolerated, and the associated products **2f**–**2m** were acquired in 49–83% yields. In addition, when 7-(2-fluorophenyl)-1*H*-indole (**1n**) was used as a substrate, it could smoothly be transformed into the target product **2n**/**2n’** in 72% yield. The formation of the monosilylation product (**2n**) in this reaction is presumably dictated by steric hindrance. Importantly, the 7-(3-fluorophenyl)-1*H*-indole could smoothly be transformed into the target product **2o** in 70% yield. To probe the electronic effects, we next screened 7-phenyl-1*H*-indole bearing electron-donating substituents on the 7-phenyl ring. 7-Phenyl-1*H*-indoles substituted with electron-donating (CH_3_, OMe, and SMe) at the *para*-position on the 7-phenyl ring all reacted satisfactorily with NBE and hexamethyldisilane to afford the corresponding monosilylation products **2p**–**2r** in 67–87% yields. The CH_3_ at the *ortho*-position on the 7-phenyl ring of 7-phenyl-1*H*-indole could also be compatible, giving corresponding product **2s** in 81% yield. Substrates with CH_3_ at *meta*-position on the 7-phenyl ring gave **2t**/**2t′** in 80% yield. Similarly, a substituent (such as OMe) at the *meta*-position on the 7-phenyl ring, and the reaction also reacted smoothly to afford the disilylation product **2u** in 86% yield. In the case of using 3-(1*H*-indol-7-yl)-*N*, *N*-dimethylaniline (**1v**) as the substrate, the disilylation product **2v** could be obtained in 41% yield. The reason for the formation of the disilylation products (**2t’**–**2v**) may be the weak electron-donating effects of the substitutes at *meta*-position on the 7-phenyl ring, which is beneficial for C–H activation and helps stabilize the palladacycle intermediate. Finally, we examined 7-polycyclic and 7-heterocyclic substituent indoles. In the case of using 7-(naphthalen-1-yl)-1*H*-indole as the substrate, the monosilylation/*N*-alkylation product **2w** could be obtained in 84% yield. It was noteworthy that 7-(benzo[*d*][1,3]dioxol-5-yl)-1*H*-indole (**1x**) was also a suitable substrate, albeit giving a decreased yield. The carbazolyl at *para*-position on the 7-phenyl ring of 7-phenyl-1*H*-indole reacted well with NBE and hexamethyldisilane to give the corresponding monosilylation product **2y** 77% yields. These results indicate that the electronic effect may play an important role in this transformation. We suspect that electron-withdrawing groups and electron-donating groups are beneficial for the disilylation and monosilylation reaction of 7-phenyl-1*H*-indole, respectively. In the case of electron-withdrawing groups, C–H activation is favored, forming a palladacycle intermediate. For electron-donating groups, however, C–H activation is suppressed, diverting the mechanism toward aminopalladation via the NBE pathway, which delivers the monosilylated product.

**Scheme 3:**
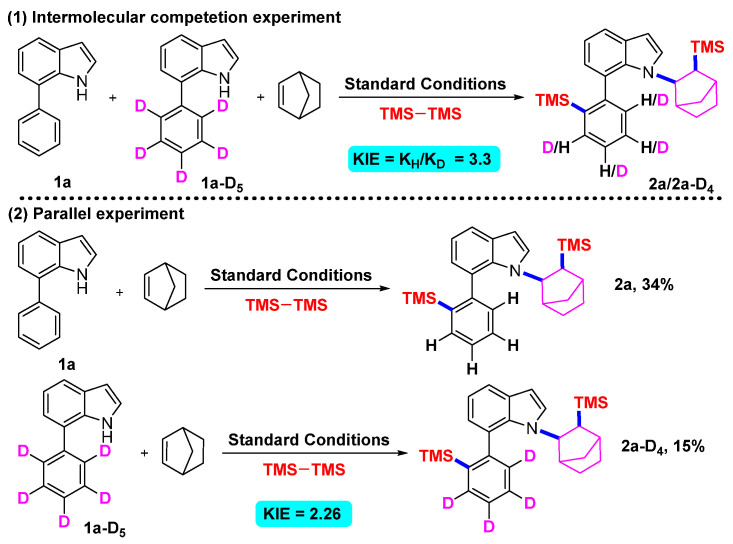
Control experiments.

To investigate the reaction mechanism, kinetic isotope effect (KIE) studies were performed using substrates **1a** and **1a-[D_5_]** in control experiments. The measured KIE values were 3.3 and 2.26 in the presence of norbornene, respectively (Scheme 3). These kinetic results indicate that C–H bond activation was the rate-determining step in this transformation.

**Scheme 4:**
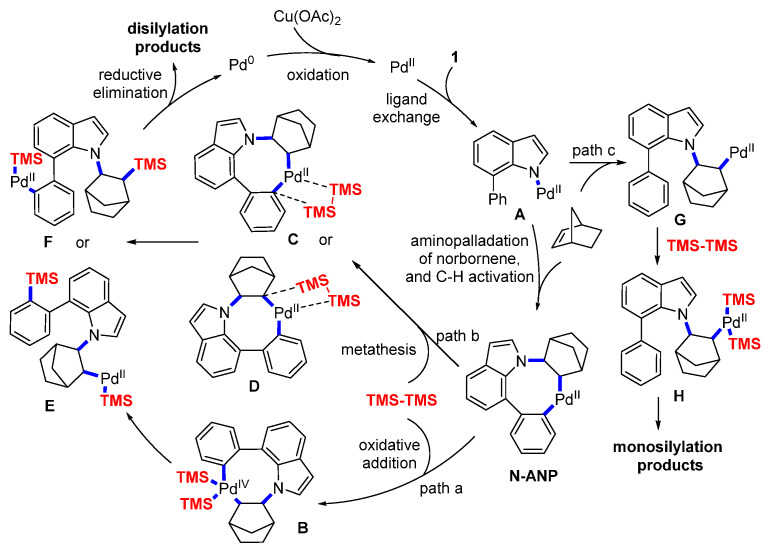
Possible mechanism.

On the basis of the above findings and literature reports [21], a reaction mechanism has been proposed and is shown in Scheme 4. First, intermediate **A** is formed via ligand exchange. The reaction of **N−Pd(II)** species with NBE to afford the eight-membered palladacycle **N-ANP** by aminopalladation of NBE and C−H activation. Next, the reaction of the **N-ANP** with hexamethyldisilane forms the intermediate **E** or **F** via two paths (path a: oxidative addition of the **N-ANP** to hexamethyldisilane, followed by reductive elimination; path b: the σ bond metathesis pathway involving intermediate **C** or **D**). Finally, reductive elimination of the intermediate **E** or **F** takes place to provide the desired disilylation products. Different from disilylation products, monosilylation products are obtained via aminopalladation of NBE without C−H activation (path c: oxidative addition of the intermediate **G** to hexamethyldisilane, followed by reductive elimination).

## 3. Materials and Methods

### 3.1. Materials

^1^H and ^13^C NMR spectra were recorded on a Bruker ARX400 spectrometer (Bruker, Billerica, MA, USA; FT, 400 MHz for ^1^H; 100 MHz for ^13^C) at room temperature, unless otherwise noted. Chemical shifts were reported in ppm on the scale relative to CDCl_3_ (δ = 7.26 for ^1^H-NMR, δ = 77.00 for ^13^C-NMR) or DMSO-*d*_6_ (δ = 2.50 for ^1^H-NMR, δ = 39.60 for ^13^C-NMR) as an internal reference. Multiplicities are reported using the following abbreviations: s = singlet, d = doublet, t = triplet, q = quartet, m = multiplet. HPLC/Q-TOF-MS analysis was performed with an Agilent 1290 LC system, coupled with a 6530Q-TOF/MS accurate-mass spectrometer (Agilent Technologies, 5301 Stevens Creek Blvd, Santa Clara, CA, USA). Mass spectrometry was performed in positive electrospray ionization (ESI+) mode. Reactions were monitored by thin-layer chromatography. Column chromatography (petroleum ether/ethyl acetate) was performed on silica gel (200–300 mesh). Unless otherwise noted, all starting materials were commercially available and were used without further purification.

### 3.2. General Methods for the Preparation of 1-((2R,3S)-3-(Trimethylsilyl)bicyclo[2.2.1]heptan-2-yl)-7-(2-(trimethylsilyl)phenyl)-1H-indole Derivatives

The stirred mixture of 7-phenyl-1*H*-indole (**1**, 0.3 mmol), NBE (2 equiv), Hexamethyldisilane (2.0 equiv), Pd(OAc)_2_ (10 mol %), Cu(OAc)_2_ (3.0 equiv), and K_2_CO_3_ (2 equiv), DABCO (1.0 equiv) in CH_3_CN (3 mL) at 100 °C for 24 h. After completion of the reaction (monitored by TLC), the reaction mixture was filtered, the filtrate was evaporated under reduced pressure, and the crude product was purified by column chromatography (petroleum ether:ethyl acetate =100:1 unless otherwise noted) to provide the desired products.

1-((2*R*,3*S*)-3-(trimethylsilyl)bicyclo[2.2.1]heptan-2-yl)-7-(2-(trimethylsilyl)phenyl)-1*H*-indole **(2a)**: pale yellow oil, isolated yield 75% (97 mg). ^1^H NMR (400 MHz, CDCl_3_) δ 7.52 (d, *J* = 7.60 Hz, 2H), 7.47 (dd, *J* = 7.36 Hz, 1.50 Hz, 1H), 7.16 (d, J = 7.60 Hz, 1H), 7.12 (t, *J* = 7.44 Hz, 1H), 7.05 (t, *J* = 7.68 Hz, 1H), 7.02–7.00 (m, 2H), 6.48–6.46 (m, 1H), 3.43 (d, *J* = 11.13 Hz, 1H), 1.92 (d, *J* = 3.59 Hz, 1H), 1.69 (s, 1H), 1.28 (d, *J* = 1.43 Hz, 1H), 1.25 (d, J = 1.43 Hz, 1H), 1.04 (s, 1H), 0.80 (d, J = 2.23 Hz, 1H), 0.78 (s, 1H), 0.53 (d, *J* = 10.04 Hz, 1H), 0.35 (s, 9H), 0.25 (d, *J* = 9.88 Hz, 1H), −0.39 (s, 9H). ^13^C NMR (100 MHz, CDCl_3_) δ 149.8, 142.6, 136.0, 136.0, 135.2, 134.9, 128.7, 126.8, 125.3, 123.8, 122.8, 119.6, 119.4, 102.7, 54.7, 43.9, 39.9, 38.9, 38.5, 33.1, 32.5, 2.2, −0.3. HRMS (ESI) *m*/*z* calcd for C_27_H_38_NSi_2_+ (M + H)^+^ 432.2537, found 432.2533.

5-fluoro-1-((2*R*,3*S*)-3-(trimethylsilyl)bicyclo[2.2.1]heptan-2-yl)-7-(2-(trimethylsilyl)phenyl)-1*H*-indole **(2b)**: pale yellow oil, isolated yield 73% (98 mg). ^1^H NMR (400 MHz, CDCl_3_) δ 8.10 (s, 1H), 7.63 (d, *J* = 7.67 Hz, 2H), 7.56–7.52 (m, 3H), 7.43 (t, *J* = 7.35 Hz, 1H), 6.97–6.94 (m, 1H), 3.39 (d, *J* = 10.61 Hz, 1H), 2.68 (s, 1H), 2.44 (d, *J* = 3.43 Hz, 1H), 2.16 (d, *J* = 9.30 Hz, 1H), 1.88–1.82 (m, 1H), 1.71–1.67 (m, 1H), 1.50 (d, *J* = 9.79 Hz, 2H), 1.43 (s, 1H), 0.41 (d, *J* = 4.57 Hz, 1H), 0.36 (s, 9H), −0.39 (s, 9H). ^13^C NMR (100 MHz, CDCl_3_) δ 157.4 (d, *J* = 233.53 Hz), 138.4, 136.9, 133.2, 129.3, 129.0 (d, *J* = 5.35 Hz), 128.1, 127.8, 127.5, 127.1 (d, *J* = 9.82 Hz), 126.9, 125.8 (d, *J* = 9.82 Hz), 109.9 (d, *J* = 27.22 Hz), 105.5 (d, *J* = 24.09 Hz), 45.0, 43.8, 41.6, 39.3, 33.3, 32.3, 29.7, −0.2, −0.5. HRMS (ESI) *m*/*z* calcd for C_27_H_37_FNSi_2_+ (M + H)^+^ 450.2443, found 450.2442.

5-chloro-1-((2*R*,3*S*)-3-(trimethylsilyl)bicyclo[2.2.1]heptan-2-yl)-7-(2-(trimethylsilyl)phenyl)-1*H*-indole **(2c)**: pale yellow oil, isolated yield 70% (98 mg). ^1^H NMR (400 MHz, CDCl_3_) δ 8.12 (s, 1H), 7.82 (s, 1H), 7.61 (d, *J* = 7.31 Hz, 2H), 7.53 (t, *J* = 7.31 Hz, 2H), 7.43 (t, *J* = 7.31 Hz, 1H), 7.14 (s, 1H), 3.36 (d, *J* = 10.48 Hz, 1H), 2.66 (s, 1H), 2.43 (s, 1H), 2.15 (d, *J* = 9.06 Hz, 1H), 1.86–1.82 (d, *J* = 10.64 Hz, 1H), 1.67 (s, 1H), 1.50 (d, *J* = 9.21 Hz, 1H), 1.42 (s, 1H), 0.87 (d, *J* = 6.35 Hz, 2H), 0.35 (s, 9H), −0.41 (s, 9H). ^13^C NMR (100 MHz, CDCl_3_) δ 138.2, 136.5, 135.0, 129.3, 128.7, 128.1, 128.0, 127.8, 126.2, 124.7, 121.6, 120.1, 119.2, 101.4, 45.0, 43.8, 41.7, 39.4, 39.3, 33.3, 32.3, −0.3, −0.6. HRMS (ESI) *m*/*z* calcd for C_27_H_37_ClNSi_2_+ (M + H)^+^ 466.2148, found 466.2141.

5-nitro-1-((2*R*,3*S*)-3-(trimethylsilyl)bicyclo[2.2.1]heptan-2-yl)-7-(2-(trimethylsilyl)phenyl)-1*H*-indole **(2d)**: pale yellow oil, isolated yield 63% (90 mg). ^1^H NMR (400 MHz, CDCl_3_) δ 8.57 (d, *J* = 1.93 Hz, 1H), 7.90 (d, *J* = 1.79 Hz, 1H), 7.46 (s, 4H), 7.36 (s, 2H), 4.24 (d, *J* = 9.83 Hz, 1H), 2.35 (d, *J* = 3.18 Hz, 1H), 2.24 (d, *J* = 2.75 Hz, 1H), 1.79 (d, *J* = 10.12 Hz, 1H), 1.30–1.26 (m, 4H), 0.79–0.75 (m, 2H), 0.40 (s, 9H), −0.42 (s, 9H). ^13^C NMR (100 MHz, CDCl_3_) δ 140.3, 139.1, 139.1, 134.8, 132.2, 129.9, 128.7, 128.2, 128.1, 128.0, 126.7, 119.6, 117.7, 113.4, 62.0, 43.5, 43.5, 38.7, 37.9, 31.5, 29.6, −0.3, −1.3. HRMS (ESI) *m*/*z* calcd for C_27_H_37_N_2_O_2_Si_2_+ (M + H)^+^ 477.2388, found 477.2390.

1-((2*R*,3*S*)-3-(trimethylsilyl)bicyclo[2.2.1]heptan-2-yl)-7-(2-(trimethylsilyl)phenyl)-1*H*-indole-3-carbaldehyde **(2e)**: pale yellow oil, isolated yield 74% (102 mg). ^1^H NMR (400 MHz, CDCl_3_) δ 9.97 (s, 1H), 8.46 (s, 1H), 8.25 (d, *J* = 7.84 Hz, 1H), 7.76 (d, *J* = 7.27 Hz, 1H), 7.56 (d, *J* = 7.27 Hz, 1H), 7.38 (d, *J* = 7.05 Hz, 1H), 7.34 (d, *J* = 7.73 Hz, 1H), 7.20 (t, *J* = 7.27 Hz, 1H), 7.06 (d, *J* = 7.27 Hz, 1H), 3.52 (d, *J* = 10.91 Hz, 1H), 2.02 (s, 1H), 1.71 (s, 1H), 1.49 (d, *J* = 3.64 Hz, 1H), 1.35 (d, *J* = 11.37 Hz, 1H), 1.25 (s, 2H), 1.14 (d, *J* = 9.77 Hz, 2H), 0.56 (d, *J* = 9.32 Hz, 1H), 0.41 (s, 9H), −0.32 (s, 9H). ^13^C NMR (100 MHz, CDCl_3_) δ 185.4, 149.7, 143.0, 136.9, 135.6, 135.2, 134.7, 134.7, 129.2, 125.4, 125.1, 123.4, 122.6, 120.9, 119.8, 54.5, 44.0, 40.2, 38.9, 38.5, 33.1, 32.4, 2.2, −0.3. HRMS (ESI) *m*/*z* calcd for C_28_H_38_NOSi_2_+ (M + H)^+^ 460.2486, found 460.2482.

7-(4-fluoro-2-(trimethylsilyl)phenyl)-1-((2*R*,3*S*)-3-(trimethylsilyl)bicyclo[2.2.1]heptan-2-yl)-1*H*-indole **(2f)**: pale yellow oil, isolated yield 72% (97 mg). ^1^H NMR (400 MHz, CDCl_3_) δ 7.87 (d, *J* = 7.97 Hz, 1H), 7.57 (t, *J* = 7.40 Hz, 2H), 7.45 (s, 1H), 7.43 (s, 1H), 7.32 (d, *J* = 8.54 Hz, 1H), 7.29 (d, *J* = 7.40 Hz, 1H), 7.11 (d, *J* = 7.11 Hz, 1H), 4.43 (d, *J* = 9.67 Hz, 1H), 2.54 (d, *J* = 3.70 Hz, 1H), 2.44 (s, 1H), 2.03 (d, *J* = 9.96 Hz, 1H), 1.75–1.73 (m, 1H), 1.65–1.61 (m, 1H), 1.45 (s, 2H), 0.98 (d, *J* = 9.67 Hz, 2H), 0.55 (s, 9H), −0.22 (s, 9H). ^13^C NMR (100 MHz, CDCl_3_) δ 137.5, 136.0, 133.0, 131.9, 131.7 (d, *J* = 7.29 Hz), 130.5 (d, *J* = 7.87 Hz), 125.5, 124.6, 121.3, 120.4, 118.5, 114.6, 114.4, 109.5, 61.7, 43.7, 38.7, 37.9, 37.8, 31.7, 29.7, −0.2, −1.2. HRMS (ESI) *m*/*z* calcd for C_27_H_37_FNSi_2_+ (M + H)^+^ 450.2443, found 450.2442.

3-(trimethylsilyl)-4-(1-((2*R*,3*S*)-3-(trimethylsilyl)bicyclo[2.2.1]heptan-2-yl)-1*H*-indol-7-yl)benzonitrile **(2g)**: pale yellow oil, isolated yield 74% (101 mg). ^1^H NMR (400 MHz, CDCl_3_) δ 7.78 (d, *J* = 7.96 Hz, 1H), 7.73 (d, *J* = 7.68 Hz, 1H), 7.69 (d, *J* = 8.05 Hz, 1H), 7.63 (t, *J* = 7.96 Hz, 1H), 7.59 (d, *J* = 5.85 Hz, 1H), 7.08 (t, *J* = 7.23 Hz, 1H), 7.00 (s, 1H), 6.90 (d, *J* = 7.23 Hz, 1H), 4.62 (d, *J* = 9.51 Hz, 1H), 2.89 (s, 1H), 2.10 (s, 1H), 1.70 (d, *J* = 10.06 Hz, 1H), 1.57 (s, 2H), 1.51–1.49 (m, 1H), 1.16 (d, *J* = 9.79 Hz, 1H), 0.93 (d, *J* = 10.43 Hz, 1H), 0.89–0.86 (m, 1H), 0.43 (s, 9H), −0.68 (s, 9H). ^13^C NMR (100 MHz, CDCl_3_) δ 140.4, 138.8, 131.9, 129.5, 129.1, 127.8, 127.4, 126.5, 125.0, 124.1, 123.4, 121.5, 121.3, 119.0, 118.3, 65.2, 46.2, 40.8, 39.2, 38.9, 31.3, 31.2, 3.2, −0.4. HRMS (ESI) *m*/*z* calcd for C_28_H_37_N_2_Si_2_+ (M + H)^+^ 457.2490, found 457.2482.

7-(4-nitro-2-(trimethylsilyl)phenyl)-1-((2*R*,3*S*)-3-(trimethylsilyl)bicyclo[2.2.1]heptan-2-yl)-1*H*-indole **(2h)**: pale yellow oil, isolated yield 63% (90 mg). ^1^H NMR (400 MHz, CDCl_3_) δ 8.33 (dd, *J* = 19.74 Hz, 8.43 Hz, 1H), 8.22 (d, *J* = 8.43 Hz, 1H), 7.74 (d, *J* = 8.11 Hz, 1H), 7.57 (d, *J* = 8.43 Hz, 1H), 7.10 (t, *J* = 7.46 Hz, 1H), 7.02 (s, 1H), 6.92 (d, *J* = 6.81 Hz, 1H), 4.64 (d, *J* = 9.40 Hz, 1H), 2.91 (s, 1H), 2.10 (s, 1H), 1.71 (d, *J* = 9.40 Hz, 1H), 1.56 (s, 1H), 1.48 (d, *J* = 6.81 Hz, 1H), 1.16 (d, *J* = 9.73 Hz, 1H), 0.96 (d, *J* = 9.08 Hz, 1H), 0.87 (d, *J* = 7.46 Hz, 2H), 0.43 (s, 9H), −0.67 (s, 9H). ^13^C NMR (100 MHz, CDCl_3_) δ 147.5, 140.2, 138.8, 132.0, 131.8, 130.3, 130.1, 129.4, 126.4, 125.4, 121.1, 119.0, 118.2, 110.7, 65.1, 46.2, 40.8, 39.2, 38.9, 31.3, 31.1, 3.2, −1.4. HRMS (ESI) *m*/*z* calcd for C_27_H_37_N_2_O_2_Si_2_+ (M + H)^+^ 477.2388, found 477.2389.

methyl 3-(trimethylsilyl)-4-(1-((2*R*,3*S*)-3-(trimethylsilyl)bicyclo[2.2.1]heptan-2-yl)-1*H*-indol-7-yl)benzoate **(2i)**: pale yellow oil, isolated yield 67% (98 mg). ^1^H NMR (400 MHz, CDCl_3_) δ 8.14 (dd, *J* = 7.96 Hz, 1.41 Hz, 1H), 8.02 (dd, *J* = 7.96 Hz, 1.41 Hz, 1H), 7.66–7.63 (m, 1H), 7.59 (d, *J* = 7.71 Hz, 1H), 7.48 (d, *J* = 7.84 Hz, 1H), 7.08 (t, *J* = 7.45 Hz, 1H), 7.00 (s, 1H), 6.93 (d, *J* = 7.07 Hz, 1H), 4.75 (d, *J* = 9.51 Hz, 1H), 3.97 (s, 3H), 2.88 (s, 1H), 2.06 (s, 1H), 1.70 (d, *J* = 9.76 Hz, 1H), 1.46–1.43 (m, 2H), 1.14 (d, *J* = 9.68 Hz, 1H), 0.99–0.95 (m, 2H), 0.50 (dd, *J* = 9.48 Hz, 1.50 Hz, 1H), 0.43 (s, 9H), −0.67 (s, 9H). ^13^C NMR (100 MHz, CDCl_3_) δ 167.0, 147.4, 139.8, 139.0, 131.1, 130.2, 129.4, 128.9, 128.5, 127.6, 126.5, 126.3, 120.6, 118.9, 118.0, 64.9, 52.2, 46.0, 40.8, 39.3, 38.8, 31.2, 31.1, 3.2, −0.4. HRMS (ESI) *m*/*z* calcd for C_29_H_40_NO_2_Si_2_+ (M + H)^+^ 490.2592, found 490.2586.

tert-butyl 3-(trimethylsilyl)-4-(1-((2*R*,3*S*)-3-(trimethylsilyl)bicyclo[2.2.1]heptan-2-yl)-1*H*-indol-7-yl)benzoate **(2j)**: pale yellow oil, isolated yield 69% (110 mg). ^1^H NMR (400 MHz, CDCl_3_) δ 8.30 (s, 1H), 8.12 (s, 1H), 8.04 (d, *J* = 7.34 Hz, 1H), 7.90 (d, *J* = 8.07 Hz, 1H), 7.80 (d, *J* = 7.71 Hz, 1H), 7.59 (t, *J* = 7.71 Hz, 1H), 7.19–7.12 (m, 2H), 4.12 (dd, *J* = 13.94 Hz, 6.60 Hz, 1H), 3.41 (d, *J* = 10.64 Hz, 1H), 2.72 (s, 1H), 2.43 (s, 1H), 2.23 (d, *J* = 8.81 Hz, 1H), 2.05 (s, 1H), 1.83 (d, *J* = 10.27 Hz, 1H),, 1.61 (s, 9H), 1.48 (d, *J* = 8.81 Hz, 1H), 1.43 (s, 1H), 1.26 (s, 1H), 0.36 (s, 9H), −0.42 (s, 9H). ^13^C NMR (100 MHz, CDCl_3_) δ 165.5, 139.5, 136.4, 134.8, 132.8, 132.1, 129.2, 129.0, 128.9, 128.2, 127.2, 124.3, 121.6, 121.6, 119.1, 81.2, 60.4, 45.1, 43.7, 41.7, 39.3, 33.3, 32.4, 28.2, −0.2, −0.6. HRMS (ESI) *m*/*z* calcd for C_32_H_46_NO_2_Si_2_+ (M + H)^+^ 532.3062, found 532.3061.

7-(4-(methylsulfonyl)-2-(trimethylsilyl)phenyl)-1-((2*R*,3*S*)-3-(trimethylsilyl)bicyclo[2.2.1]heptan-2-yl)-1*H*-indole **(2k)**: pale yellow oil, isolated yield 83% (127 mg). ^1^H NMR (400 MHz, CDCl_3_) δ 8.06–8.00 (m, 2H), 7.73 (d, *J* = 7.79 Hz, 1H), 7.67 (d, *J* = 7.30 Hz, 1H), 7.63 (d, *J* = 8.28 Hz, 1H), 7.25 (s, 1H), 7.12 (t, *J* = 7.79 Hz, 1H), 6.92 (d, *J* = 7.30 Hz, 1H), 4.07 (d, *J* = 9.74 Hz, 1H), 3.13 (s, 3H), 2.37 (d, *J* = 2.43 Hz, 1H), 2.26 (s, 1H), 1.84 (d, *J* = 9.74 Hz, 1H), 1.52 (d, *J* = 11.69 Hz, 1H), 0.88 (d, *J* = 7.79 Hz, 2H), 0.76 (d, *J* = 9.25 Hz, 2H), 0.67 (d, *J* = 11.20 Hz, 1H), 0.37 (s, 9H), −0.40 (s, 9H). ^13^C NMR (100 MHz, CDCl_3_) δ 147.7, 139.3, 135.4, 133.2, 132.0, 131.2, 129.9, 126.9, 126.6, 124.4, 124.1, 122.0, 118.6, 110.0, 62.0, 44.6, 43.7, 43.7, 38.7, 37.8, 31.7, 29.7, −0.2, −1.2. HRMS (ESI) *m*/*z* calcd for C_28_H_40_NO_2_SSi_2_+ (M + H)^+^ 510.2313, found 510.2299.

7-(4-(trifluoromethyl)-2-(trimethylsilyl)phenyl)-1-((2*R*,3*S*)-3-(trimethylsilyl)bicyclo[2.2.1]heptan-2-yl)-1*H*-indole **(2l)**: pale yellow oil, isolated yield 64% (96 mg). ^1^H NMR (400 MHz, CDCl_3_) δ 8.36 (d, *J* = 7.88 Hz, 1H), 8.23 (d, *J* = 8.71 Hz, 1H), 7.74 (d, *J* = 8.29 Hz, 1H), 7.63 (d, *J* = 7.88 Hz, 1H), 7.57 (d, *J* = 8.71 Hz, 1H), 7.10 (t, *J* = 14.93 Hz, 1H), 7.02 (s, 1H), 6.92 (d, *J* = 7.05 Hz, 1H), 4.64 (d, *J* = 9.12 Hz, 1H), 2.91 (s, 1H), 2.10 (s, 1H), 1.72–1.68 (m, 2H), 1.57 (s, 1H), 1.49 (d, *J* = 6.64 Hz, 2H), 1.16 (d, *J* = 9.95 Hz, 2H), 0.44 (s, 9H), −0.67 (s, 9H). ^13^C NMR (100 MHz, CDCl_3_) δ 149.6, 146.7, 140.4, 138.8, 131.9, 130.4, 129.5, 126.5, 125.0, 124.2 (q, *J* = 22.01 Hz), 123.4 121.5, 121.3, 119.0, 118.2, 65.2, 46.2, 40.8, 39.2, 38.9, 31.3, 31.1, 3.2, −0.4. HRMS (ESI) *m*/*z* calcd for C_28_H_37_F_3_NSi_2_+ (M + H)^+^ 500.2411, found 500.2416.

7-(4-phenoxy-2-(trimethylsilyl)phenyl)-1-((2*R*,3*S*)-3-(trimethylsilyl)bicyclo[2.2.1]heptan-2-yl)-1*H*-indole **(2m)**: pale yellow oil, isolated yield 49% (77 mg). ^1^H NMR (400 MHz, CDCl_3_) δ 7.56 (d, *J* = 7.69 Hz, 1H), 7.53 (d, *J* = 8.07 Hz, 1H), 7.39–7.33 (m, 3H), 7.14 (d, *J* = 7.20 Hz, 1H), 7.11–7.07 (m, 4H), 7.04 (d, *J* = 7.09 Hz, 1H), 7.01–6.96 (m, 2H), 4.87 (d, *J* = 9.52 Hz, 1H), 2.89 (s, 1H), 2.14 (s, 1H), 1.75 (d, *J* = 9.52 Hz, 1H), 1.68 (s, 1H), 1.57 (s, 1H), 1.18 (d, *J* = 9.66 Hz, 2H), 1.09 (d, *J* = 8.28 Hz, 1H), 0.64 (d, *J* = 9.39 Hz, 1H), 0.43 (s, 9H), −0.66 (s, 9H). ^13^C NMR (100 MHz, CDCl_3_) δ 157.4, 156.3, 139.6, 139.5, 137.4, 132.3, 130.1, 130.0, 129.8, 126.9, 126.7, 123.2, 120.0, 118.9, 118.8, 118.3, 118.1, 117.4, 64.8, 46.1, 40.8, 39.4, 39.0, 31.2, 31.2, 3.3, −0.4. HRMS (ESI) *m*/*z* calcd for C_33_H_42_NOSi_2_+ (M + H)^+^ 524.2799, found 524.2791.

7-(2-fluorophenyl)-1-((2*R*,3*S*)-3-(trimethylsilyl)bicyclo[2.2.1]heptan-2-yl)-1*H*-indole and (7*S*)-7-(2-fluoro-6-(trimethylsilyl)phenyl)-1-((2*R*,3*S*)-3-(trimethylsilyl)bicyclo[2.2.1]heptan-2-yl)-1*H*-indole **(2n:2n’ = 3:1)**: pale yellow oil, isolated yield 72% (85 mg). ^1^H NMR (400 MHz, CDCl_3_) δ 7.86–7.82 (m, 0.66H), 7.56 (d, *J* = 7.78 Hz, 1H), 7.49 (t, *J* = 7.45 Hz, 0.33H), 7.37–7.31 (m, 1.67H), 7.25 (d, *J* = 7.13 Hz, 0.33H), 7.22 (d, *J* = 7.45 Hz, 0.33H), 7.19–7.15 (m, 2H), 7.12 (d, *J* = 10.21 Hz, 1H), 7.07 (d, *J* = 9.07 Hz, 1H), 7.03 (d, *J* = 7.61 Hz, 1H), 7.00 (s, 0.33H), 6.88 (dd, *J* = 13.93 Hz, 7.13 Hz, 1H), 6.51–6.49 (m, 1H), 4.20 (dd, *J* = 19.44 Hz, 9.56 Hz, 1H), 3.34 (d, *J* = 10.53 Hz, 0.33H), 2.65 (s, 0.33H), 2.36–2.35 (m, 1H), 2.15 (d, *J* = 10.53 Hz, 1.67H), 1.97 (s, 0.33H), 1.77–1.72 (m, 1.33H), 1.62–1.58 (m, 0.33H), 1.48–1.45 (m, 1H), 1.42–1.41 (m, 1H), 1.39–1.35 (m, 1H), 1.24 (s, 0.33H), 1.14 (d, *J* = 10.16 Hz, 0.66H), 0.87 (d, *J* = 9.20 Hz, 1H), 0.73 (d, *J* = 9.52 Hz, 1H), 0.55 (d, *J* = 9.20 Hz, 1H), 0.27 (s, 3H), −0.46 (s, 3H), −0.51 (d, *J* = 6.66 Hz, 9H). ^13^C NMR (100 MHz, CDCl_3_) δ 136.7, 134.7, 134.3, 132.7 (d, *J* = 3.17 Hz), 131.5 (d, *J* = 3.04 Hz), 129.4 (d, *J* = 7.59 Hz), 129.3, 129.2, 129.1 (d, *J* = 4.27 Hz), 128.9 (d, *J* = 12.34 Hz), 128.7, 127.1, 125.3, 124.7 (d, *J* = 6.64 Hz), 123.7 (d, *J* = 3.32 Hz), 123.6 (d, *J* = 3.32 Hz), 123.0, 121.9, 120.9, 120.8, 119.4, 119.2 (d, *J* = 5.20 Hz), 118.7, 118.5 (d, *J* = 2.31 Hz), 116.2 (d, *J* = 22.54 Hz), 115.5 (d, *J* = 21.97 Hz), 114.9 (d, *J* = 21.97 Hz), 101.7 (d, *J* = 16.19 Hz), 61.2 (d, *J* = 5.78 Hz), 45.1, 43.9, 43.8 (d, *J* = 3.24 Hz), 43.7 (d, *J* = 3.78 Hz), 41.7, 39.3 (d, *J* = 5.93 Hz), 38.7, 37.7, 37.4, 33.3, 32.4, 31.7, 29.7, −0.2, −0.6, −1.2.

7-(5-fluoro-2-(trimethylsilyl)phenyl)-1-((2*R*,3*S*)-3-(trimethylsilyl)bicyclo[2.2.1]heptan-2-yl)-1*H*-indole **(2o)**: pale yellow oil, isolated yield 70% (94 mg). ^1^H NMR (400 MHz, CDCl_3_) δ 7.71–7.69 (m, 1H), 7.39–7.38 (m, 1H), 7.25 (s, 1H), 7.22–7.18 (m, 1H), 7.13–7.08 (m, 3H), 6.94 (d, *J* = 6.77 Hz, 1H), 4.22 (d, *J* = 9.41 Hz, 1H), 2.35 (s, 1H), 2.26 (s, 1H), 1.85 (d, *J* = 9.79 Hz, 1H), 1.69 (s, 1H), 1.54–1.51 (m, 2H), 0.87 (s, 2H), 0.43 (d, *J* = 6.02 Hz, 1H), 0.37 (s, 9H), −0.39 (s, 9H). ^13^C NMR (100 MHz, CDCl_3_) δ 143.8, 135.7, 133.0, 131.9, 129.2, 126.1, 125.4 (d, *J* = 17.49 Hz), 124.2, 121.5, 120.6, 118.5, 113.9, 109.7, 101.8, 61.7 (d, *J* = 12.33 Hz), 43.8, 43.6 (d, *J* = 14.80 Hz), 38.7, 37.9, 31.9, 31.7, −0.2, −1.2. HRMS (ESI) *m*/*z* calcd for C_27_H_37_FNSi_2_+ (M + H)^+^ 450.2443, found 450.2437.

7-(*p*-tolyl)-1-((2*R*,3*S*)-3-(trimethylsilyl)bicyclo[2.2.1]heptan-2-yl)-1*H*-indole **(2p)**: pale yellow oil, isolated yield 87% (97 mg). ^1^H NMR (400 MHz, CDCl_3_) δ 7.63 (d, *J* = 7.91 Hz, 1H), 7.38 (d, *J* = 6.63 Hz, 1H), 7.32 (d, *J* = 7.49 Hz, 1H), 7.30–7.28 (m, 3H), 7.11 (t, *J* = 7.27 Hz, 1H), 6.97 (d, *J* = 7.06 Hz, 1H), 6.60 (d, *J* = 3.21 Hz, 1H), 4.36 (d, *J* = 9.62 Hz, 1H), 4.16 (dd, *J* = 14.11 Hz, 7.06 Hz, 1H), 2.49 (s, 3H), 2.35 (d, *J* = 3.21 Hz, 1H), 2.26 (d, *J* = 1.92 Hz, 1H), 2.10 (s, 1H), 1.86 (d, *J* = 10.05 Hz, 1H), 1.57–1.52 (m, 1H), 1.46–1.41 (m, 1H), 0.86–0.82 (m, 2H), −0.35 (s, 9H). ^13^C NMR (100 MHz, CDCl_3_) δ 138.4, 136.7, 130.1, 128.9, 128.6, 128.2, 126.5, 125.3, 124.5, 119.9, 118.4, 101.5, 61.5, 60.4, 43.7, 43.5, 38.6, 37.8, 31.7, 21.2, −1.1. HRMS (ESI) *m*/*z* calcd for C_25_H_32_NSi+ (M + H)^+^ 374.2299, found 374.2294.

7-(4-methoxyphenyl)-1-((2*R*,3*S*)-3-(trimethylsilyl)bicyclo[2.2.1]heptan-2-yl)-1*H*-indole **(2q)**: pale yellow oil, isolated yield 82% (96 mg). ^1^H NMR (400 MHz, CDCl_3_) δ 7.60 (d, *J* = 7.86 Hz, 1H), 7.39 (d, *J* = 7.44 Hz, 1H), 7.33 (d, *J* = 7.65 Hz, 1H), 7.27 (d, *J* = 3.19 Hz, 1H), 7.08 (t, *J* = 7.44 Hz, 1H), 6.98 (d, *J* = 8.71 Hz, 2H), 6.94 (d, *J* = 7.01 Hz, 1H), 6.58 (d, *J* = 3.19 Hz, 1H), 4.40 (d, *J* = 9.56 Hz, 1H), 3.90 (s, 3H), 2.34 (d, *J* = 3.40 Hz, 1H), 2.25 (d, *J* = 2.34 Hz, 1H), 1.85 (d, *J* = 9.99 Hz, 1H), 1.55–1.51 (m, 1H), 1.45–1.41 (m, 1H), 1.28 (s, 2H), 0.86–0.84 (m, 2H), −0.37 (s, 9H). ^13^C NMR (100 MHz, CDCl_3_) δ 158.8, 134.5, 133.8, 130.0, 128.7, 126.2, 125.3, 124.8, 120.0, 118.5, 113.1, 101.6, 61.6, 55.4, 43.8, 43.6, 38.7, 37.8, 31.7, 29.7, −1.1. HRMS (ESI) *m*/*z* calcd for C_25_H_32_NOSi+ (M + H)^+^ 390.2248, found 390.2242.

7-(4-(methylthio)phenyl)-1-((2*R*,3*S*)-3-(trimethylsilyl)bicyclo[2.2.1]heptan-2-yl)-1*H*-indole **(2r)**: pale yellow oil, isolated yield 67% (81 mg). ^1^H NMR (400 MHz, CDCl_3_) δ 7.48 (d, *J* = 7.71 Hz, 1H), 7.26–7.22 (m, 4H), 7.15 (s, 1H), 6.96 (t, *J* = 7.36 Hz, 1H), 6.80 (d, *J* = 7.01 Hz, 1H), 6.46 (s, 1H), 4.23 (d, *J* = 9.46 Hz, 1H), 2.44 (s, 3H), 2.21 (s, 1H), 2.13 (s, 1H), 1.72 (d, *J* = 9.81 Hz, 1H), 1.44–1.42 (m, 1H), 1.31–1.30 (m, 1H), 0.77–0.76 (m, 2H), 0.72–0.70 (m, 2H), −0.50 (s, 9H). ^13^C NMR (100 MHz, CDCl_3_) δ 138.5, 137.3, 134.3, 130.7, 129.5, 128.8, 125.8, 125.4, 124.5, 120.2, 118.5, 101.7, 61.8, 43.8, 43.6, 38.7, 37.8, 31.8, 29.7, 16.5, −1.1. HRMS (ESI) *m*/*z* calcd for C_25_H_32_NSSi+ (M + H)^+^ 406.2019, found 406.2011.

7-(o-tolyl)-1-((2*R*,3*S*)-3-(trimethylsilyl)bicyclo[2.2.1]heptan-2-yl)-1*H*-indole **(2s)**: pale yellow oil, isolated yield 81% (91 mg). ^1^H NMR (400 MHz, CDCl_3_) δ 7.58 (d, *J* = 7.88 Hz, 1H), 7.34–7.29 (m, 2H), 7.25–7.20 (m, 3H), 7.07 (t, *J* = 7.42 Hz, 1H), 6.86 (d, *J* = 6.96 Hz, 1H), 6.54 (d, *J* = 3.25 Hz, 1H), 4.09 (d, *J* = 9.97 Hz, 1H), 2.33 (s, 1H), 2.18 (s, 1H), 2.01 (s, 3H), 1.81 (d, *J* = 9.97 Hz, 1H), 1.43–1.41 (m, 2H), 0.86 (d, *J* = 7.42 Hz, 3H), 0.60 (d, *J* = 9.74 Hz, 1H), −0.45 (s, 9H). ^13^C NMR (100 MHz, CDCl_3_) δ 140.9, 137.4, 134.0, 129.3, 129.1, 128.4, 127.6, 125.5, 125.4, 125.1, 123.7, 119.9, 118.8, 101.6, 60.8, 44.1, 43.9, 38.6, 37.8, 31.6, 29.6, 20.1, −1.3. HRMS (ESI) *m*/*z* calcd for C_25_H_32_NSi+ (M + H)^+^ 374.2299, found 374.2291.

7-(*m*-tolyl)-1-((2*R*,3*S*)-3-(trimethylsilyl)bicyclo[2.2.1]heptan-2-yl)-1*H*-indole and 7-(5-methyl-2-(trimethylsilyl)phenyl)-1-((2*R*,3*S*)-3-(trimethylsilyl)bicyclo[2.2.1]heptan-2-yl)-1*H*-indole **(2t:2t’ = 1:1)**: pale yellow oil, isolated yield 80% (98 mg). ^1^H NMR (400 MHz, CDCl_3_) δ 7.57 (d, *J* = 7.76 Hz, 2H), 7.30 (t, *J* = 7.37 Hz, 2H), 7.23 (s, 4H), 7.20 (s, 2H), 7.18 (s, 1H), 7.05 (t, *J* = 7.24 Hz, 2H), 6.93 (d, *J* = 6.97 Hz, 2H), 6.55 (s, 2H), 4.42 (d, *J* = 9.74 Hz, 1H), 4.21 (d, *J* = 9.60 Hz, 1H), 2.39 (s, 6H), 2.31 (s, 1H), 2.25 (s, 1H), 2.21 (s, 2H), 1.81 (d, *J* = 9.87 Hz, 2H), 1.67 (s, 1H), 1.52–1.50 (m, 2H), 0.86 (d, *J* = 7.69 Hz, 4H), 0.80 (s, 1H), 0.77 (s, 1H), 0.75 (s, 2H), 0.73 (s, 1H), 0.07 (s, 9H), −0.36 (s, 9H), −0.41 (s, 9H). ^13^C NMR (100 MHz, CDCl_3_) δ 141.4, 137.1, 136.9, 134.4, 131.0, 130.1, 128.7, 127.7, 127.2, 126.6, 126.2, 125.3, 125.1, 124.2, 124.2, 120.0, 118.5, 101.7, 101.6, 61.7, 43.8, 43.6, 43.4, 38.7, 37.8, 37.8, 31.7, 29.7, 22.7, 21.3, 1.0, −1.1, −1.1.

7-(5-methoxy-2-(trimethylsilyl)phenyl)-1-((2*R*,3*S*)-3-(trimethylsilyl)bicyclo[2.2.1]heptan-2-yl)-1*H*-indole **(2u)**: pale yellow oil, isolated yield 76% (105 mg). ^1^H NMR (400 MHz, CDCl_3_) δ 8.35 (s, 1H), 8.04 (d, *J* = 7.84 Hz, 1H), 7.60 (t, *J* = 7.61 Hz, 1H), 7.41 (d, *J* = 6.04 Hz, 1H), 7.34 (d, *J* = 10.75 Hz, 2H), 7.29 (d, *J* = 7.61 Hz, 1H), 7.11 (d, *J* = 7.16 Hz, 1H), 4.04 (s, 3H), 3.58 (d, *J* = 10.52 Hz, 1H), 2.88 (s, 1H), 2.60 (d, *J* = 2.69 Hz, 1H), 2.40 (d, *J* = 8.95 Hz, 1H), 2.02–1.98 (m, 1H), 1.85–1.83 (m, 1H), 1.64 (d, *J* = 9.85 Hz, 1H), 1.60 (s, 1H), 1.04 (d, *J* = 8.06 Hz, 2H), 0.52 (s, 9H), −0.25 (s, 9H). ^13^C NMR (100 MHz, CDCl_3_) δ 160.3, 141.0, 136.5, 134.6, 130.1, 129.0, 127.2, 125.1, 121.5, 121.3, 120.4, 119.1, 113.6, 113.0, 55.3, 45.2, 43.9, 41.8, 39.4, 39.3, 33.3, 32.4, −0.1, −0.5. HRMS (ESI) *m*/*z* calcd for C_28_H_40_NOSi_2_+ (M + H)^+^ 462.2643, found 462.2635.

*N*,*N*-dimethyl-4-(trimethylsilyl)-3-(1-((2*R*,3*S*)-3-(trimethylsilyl)bicyclo[2.2.1]heptan-2-yl)-1*H*-indol-7-yl)aniline **(2v)**: pale yellow oil, isolated yield 41% (58 mg). ^1^H NMR (400 MHz, CDCl_3_) δ 8.28 (s, 1H), 7.85 (d, *J* = 8.27 Hz, 1H), 7.39 (t, *J* = 7.24 Hz, 1H), 7.19 (d, *J* = 6.94 Hz, 1H), 7.11 (t, *J* = 7.55 Hz, 1H), 7.01 (d, *J* = 8.97 Hz, 2H), 6.78 (d, *J* = 8.16 Hz, 1H), 3.40 (d, *J* = 10.30 Hz, 1H), 3.02 (s, 6H), 2.71 (s, 1H), 2.42 (s, 1H), 2.23 (d, *J* = 9.08 Hz, 1H), 1.82 (d, *J* = 10.60 Hz, 1H), 1.68–1.66 (m, 1H), 1.46 (d, *J* = 9.69 Hz, 1H), 1.42 (s, 1H), 1.25 (s, 2H), 0.35 (s, 9H), −0.43 (s, 9H). ^13^C NMR (100 MHz, CDCl_3_) δ 151.1, 140.3, 136.5, 134.3, 129.8, 128.8, 127.0, 126.0, 121.4, 120.9, 119.0, 116.1, 112.1, 111.3, 45.1, 43.8, 41.7, 40.6, 39.3, 39.3, 33.3, 32.4, −0.1, −0.6. HRMS (ESI) *m*/*z* calcd for C_29_H_43_N_2_Si_2_+ (M + H)^+^ 475.2959, found 475.2951.

7-(naphthalen-1-yl)-1-((2*R*,3*S*)-3-(trimethylsilyl)bicyclo[2.2.1]heptan-2-yl)-1*H*-indole **(2w)**: pale yellow oil, isolated yield 84% (103 mg). ^1^H NMR (400 MHz, CDCl_3_) δ 7.93 (d, *J* = 7.16 Hz, 2H), 7.70 (d, *J* = 7.73 Hz, 1H), 7.56 (d, *J* = 4.58 Hz, 2H), 7.48 (t, *J* = 7.73 Hz, 1H), 7.40 (d, *J* = 8.30 Hz, 1H), 7.35–7.31 (m, 1H), 7.21–7.15 (m, 2H), 7.11–7.06 (m, 1H), 6.62 (d, *J* = 2.86 Hz, 1H), 3.73 (d, *J* = 10.02 Hz, 1H), 2.07 (s, 1H), 2.00 (d, *J* = 10.59 Hz, 1H), 1.70 (d, *J* = 10.02 Hz, 1H), 1.28 (s, 2H), 1.07 (d, *J* = 9.45 Hz, 1H), 0.93–0.88 (m, 1H), 0.42 (d, *J* = 9.74 Hz, 1H), −0.42 (s, 9H), −0.51 (t, *J* = 9.74 Hz, 1H). ^13^C NMR (100 MHz, CDCl_3_) δ 138.9, 133.7, 132.8, 128.4, 128.0, 127.6, 127.5, 126.6, 126.5, 126.3, 125.9, 125.2, 125.1, 124.8, 124.0, 120.4, 118.8, 101.6, 61.2, 43.9, 43.8, 38.5, 37.6, 31.3, 28.7, −1.3. HRMS (ESI) *m*/*z* calcd for C_28_H_32_NSi+ (M + H)^+^ 410.2299, found 410.2297.

7-(benzo[*d*][1,3]dioxol-5-yl)-1-((2*R*,3*S*)-3-(trimethylsilyl)bicyclo[2.2.1]heptan-2-yl)-1*H*-indole **(2x)**: pale yellow oil, isolated yield 75% (91 mg). ^1^H NMR (400 MHz, CDCl_3_) δ 7.77 (d, *J* = 7.83 Hz, 1H), 7.66 (d, *J* = 7.39 Hz, 1H), 7.59 (t, *J* = 7.61 Hz, 2H), 7.53 (d, *J* = 6.49 Hz, 1H), 7.43 (s, 1H), 7.22 (t, *J* = 8.28 Hz, 2H), 7.11 (d, *J* = 7.16 Hz, 1H), 6.74 (d, *J* = 3.13 Hz, 1H), 5.35 (s, 2H), 4.53 (d, *J* = 9.89 Hz, 1H), 2.48 (d, *J* = 3.25 Hz, 1H), 2.40 (d, *J* = 2.32 Hz, 1H), 2.00 (d, *J* = 9.89 Hz, 1H), 1.74–1.67 (m, 1H), 1.59–1.52 (m, 1H), 1.42 (d, *J* = 12.21 Hz, 1H), 0.98 (d, *J* = 4.95 Hz, 1H), −0.22 (s, 9H). ^13^C NMR (100 MHz, CDCl_3_) δ 157.8, 136.9, 134.0, 131.2, 130.0, 128.6, 127.9, 127.3, 125.3, 124.7, 120.0, 118.4, 114.1, 113.9, 101.5, 70.0, 61.6, 43.7, 43.5, 38.6, 37.8, 31.7, −1.1. HRMS (ESI) *m*/*z* calcd for C_25_H_30_NO_2_Si+ (M + H)^+^ 404.2040, found 404.2042.

9-(4-(1-((2*R*,3*S*)-3-(trimethylsilyl)bicyclo[2.2.1]heptan-2-yl)-1*H*-indol-7-yl)phenyl)-9*H*-carbazole **(2y)**: pale yellow oil, isolated yield 77% (121 mg). ^1^H NMR (400 MHz, CDCl_3_) δ 8.20 (d, *J* = 7.11 Hz, 2H), 7.69–7.66 (m, 5H), 7.48–7.47 (m, 4H), 7.34 (s, 3H), 7.18–7.15 (m, 1H), 7.09 (d, *J* = 6.66 Hz, 1H), 6.64 (s, 1H), 4.51 (d, *J* = 9.77 Hz, 1H), 2.42 (s, 1H), 2.30 (s, 1H), 2.06 (s, 1H), 1.89 (d, *J* = 9.77 Hz, 1H), 1.33 (s, 1H), 1.27 (s, 1H), 0.98 (d, *J* = 7.55 Hz, 1H), 0.94 (d, *J* = 9.77 Hz, 1H), 0.41 (s, 1H), −0.34 (s, 9H). ^13^C NMR (100 MHz, CDCl_3_) δ 140.8, 140.8, 136.6, 134.2, 131.7, 130.5, 128.8, 126.4, 126.0, 125.4, 124.4, 123.3, 120.5, 120.4, 120.0, 118.6, 109.5, 101.8, 62.0, 43.9, 43.7, 38.7, 37.8, 31.7, 29.9, −1.1. HRMS (ESI) *m*/*z* calcd for C_36_H_37_N_2_Si+ (M + H)^+^ 525.2721, found 525.2713.

## 4. Conclusions

In conclusion, we have established an excellent method for selective C−H silylation of 7-phenyl-1*H*-indoles by palladium(II)/norbornene cooperative catalysis. Notably, the reaction exhibits a marked substituent electronic effect, which is consistent with the involvement of an eight-membered palladacycle intermediate. Further mechanistic studies and applications of this C–H silylation reaction are currently underway in our laboratory.

## Figures and Tables

**Table 1:** Optimization of the reaction conditions ^[a]^.

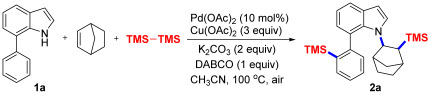					
Entry	Catalyst	Oxidant	Base	Solvent	Yield/% ^[b]^
1	Pd(OAc)_2_	Cu(OAc)_2_	K_2_CO_3_	MeCN	56
2 ^[c]^	Pd(OAc)_2_	Cu(OAc)_2_	K_2_CO_3_	MeCN	43
3	Pd_2_(dba)_3_	Cu(OAc)_2_	K_2_CO_3_	MeCN	trace ^[h]^
4	PdCl_2_(PPh_3_)_2_	Cu(OAc)_2_	K_2_CO_3_	MeCN	51
5	Pd(PPh_3_)_4_	Cu(OAc)_2_	K_2_CO_3_	MeCN	35
6	PdCl_2_	Cu(OAc)_2_	K_2_CO_3_	MeCN	27
7	/	Cu(OAc)_2_	K_2_CO_3_	MeCN	0
8	Pd(OAc)_2_	CuCl_2_	K_2_CO_3_	MeCN	0
9	Pd(OAc)_2_	BQ	K_2_CO_3_	MeCN	0
10	Pd(OAc)_2_	K_2_S_2_O_8_	K_2_CO_3_	MeCN	0
11	Pd(OAc)_2_	Cu(OAc)_2_	KOH	MeCN	16
12	Pd(OAc)_2_	Cu(OAc)_2_	*^t^*BuOK	MeCN	22
13	Pd(OAc)_2_	Cu(OAc)_2_	KOAc	MeCN	39
14	Pd(OAc)_2_	Cu(OAc)_2_	Cs_2_CO_3_	MeCN	34
15	Pd(OAc)_2_	Cu(OAc)_2_	K_3_PO_4_	MeCN	26
16	Pd(OAc)_2_	Cu(OAc)_2_	K_2_CO_3_	dioxane	trace ^[h]^
17	Pd(OAc)_2_	Cu(OAc)_2_	K_2_CO_3_	toluene	0
18	Pd(OAc)_2_	Cu(OAc)_2_	K_2_CO_3_	DMF	trace ^[h]^
19 ^[d]^	Pd(OAc)_2_	Cu(OAc)_2_	K_2_CO_3_	MeCN	75 (70) ^[g]^
20 ^[d,e]^	Pd(OAc)_2_	Cu(OAc)_2_	K_2_CO_3_	MeCN	69
21 ^[d,f]^	Pd(OAc)_2_	Cu(OAc)_2_	K_2_CO_3_	MeCN	64

^[a]^ Reaction conditions: 7-phenyl-1*H*-indole **1a** (0.3 mmol, 1.0 equiv), NBE (0.6 mmol, 2 equiv), hexamethyldisilane (0.6 mmol, 2 equiv), Pd(OAc)_2_ (10 mol%), Cu(OAc)_2_ (3 equiv), K_2_CO_3_ (2 equiv), DABCO (1 equiv), and MeCN (3 mL) at 100 °C for 24 h under an air atmosphere. ^[b]^ Isolated yield. ^[c]^ under N_2_ atmosphere. ^[d]^ 0.3 mmol of DABCO was added. ^[e]^ at 110 °C. ^[f]^ at 90 °C. ^[g]^ 1 mmol **1a** as substrate for the synthesis of **2a**. ^[h]^ trace < 10.

## Data Availability

All the data and materials described in this work are available in this article or in the Supplementary Materials.

## Supplementary Materials

The following supporting information can be downloaded at: https://www.mdpi.com/article/10.3390/molecules31152724/s1. The NMR spectra of the obtained compounds.
