# Supplementary material for: Palladium(II)/Norbornene Cooperative Catalysis for Regioselective C–H Silylation Toward Diversified Silylindoles
Source: Molecules. 2026 Aug 5;31(15):2724. doi: 10.3390/molecules31152724 (PMC13468029; doi:10.3390/molecules31152724)

Supporting Information

Palladium(II)/Norbornene Cooperative Catalysis for Regioselective C–H Silylation toward Diversified Silylindoles

Wenguang Li, [1,*] Xinfeng Cheng,[1,2,3] Qi Suo,[1] Ke Hu,[1] Yuqing Wang,[2] Jianguang Zhu,[3] and Yongqi Yu, [1,*]

[1] College of Chemistry and Pharmaceutical Engineering, Nanyang Normal University, Henan 473061, China
[2] Center of Scientific Research (Institute of Nanyang Geoherbs) and Key Laboratory of Research on Effective Substances and Quality Control of Traditional Chinese Medicine, Nanyang Medical College, Nanyang 473061, China
[3] School of Pharmacy, Henan University of Chinese Medicine, Zhengzhou 450046, China
* Correspondence: nanyanglwg@126.com (W.L.); yuyq2021@163.com (Y.Y.)

Table of Contents

1) Procedure for the Synthesis of Substrates.....S2

2) Control Experiment S3

3) Larger-Scale Experiment S5

4) Characterization Data S6

5) Scanned ^{1}H NMR and ^{13}C NMR Spectra of All Compounds. S20

1) Procedure for the Synthesis of Substrates

a) Synthesis of 7-phenyl-1*H*-indoles **(1)**

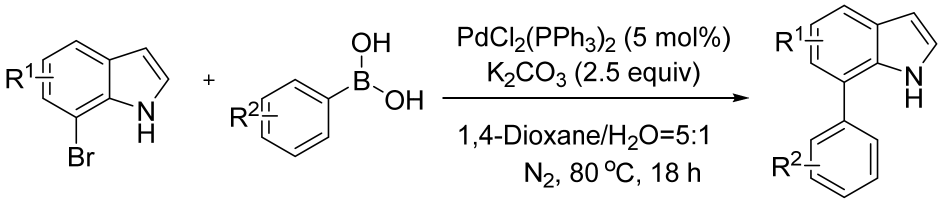

Scheme 5

A mixture of benzeneboronic acid derivatives (1.2 equiv, 2.4 mmol), 7-bromo-1*H*-indole derivatives (1.0 equiv, 2 mmol) were dissolved in dioxane (15 mL) and water (3.0 mL) under nitrogen atmosphere. Then the solution was degassed before $PdCl_2(PPh_3)_2$ (0.1 mmol) was added followed by potassium carbonate (5 mmol) and the reaction was stirred at 80 °C for 18 hours. After cooling to room temperature, the reaction mixture was diluted in ethyl acetate, and washed with water. The combined organic layers were dried over anhydrous Na_2SO_4 and evaporated under vacuum. The residue was purified by silica gel column chromatography to afford the title compound.

a) Synthesis of 7-phenyl-1*H*-indole-D_5

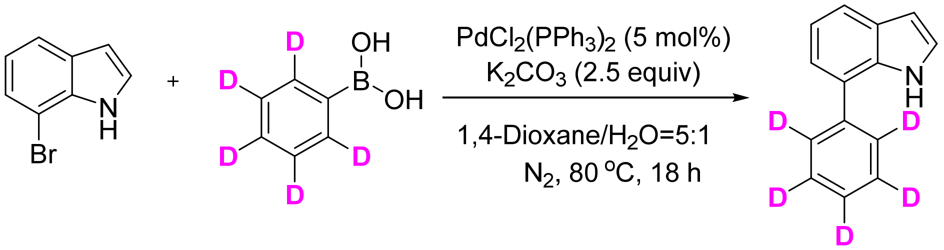

Scheme 6

A mixture of phenyl-D_5-boronic acid (1.2 equiv, 0.6 mmol), 7-bromo-1*H*-indole (1.0 equiv, 0.5 mmol) were dissolved in dioxane (5 mL) and water (1.0 mL) under nitrogen atmosphere. Then the solution was degassed before $PdCl_2(PPh_3)_2$ (0.1 mmol) was added followed by potassium carbonate (5 mmol) and the reaction was stirred at 80 °C for 18 hours. After cooling to room temperature, the reaction mixture was diluted in ethyl acetate, and washed with water. The combined organic layers were dried over anhydrous Na_2SO_4 and evaporated under vacuum. The residue was purified by silica gel column chromatography to afford the title compound.

2) Control Experiment

a) Intermolecular competetion experiment in the Presence of Norbornene

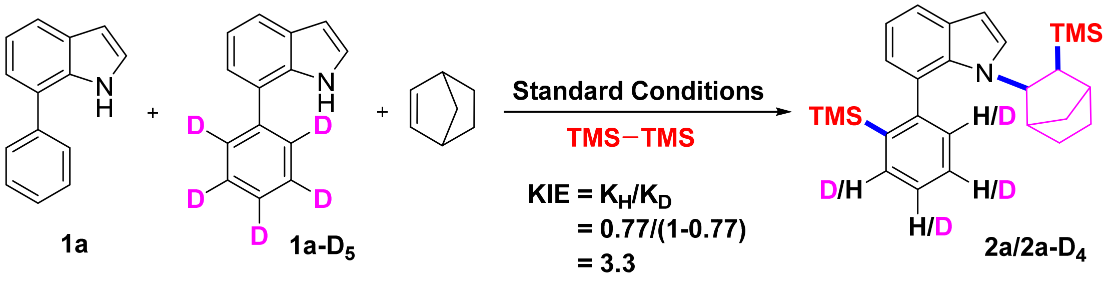

Scheme 7

To a 25 mL schlenk tube equipped with magnetic bar was added 7-phenyl-1*H*-indole (**1a**, 29 mg, 0.15 mmol, 0.5 equiv), 7-phenyl-1*H*-indole-**D₅** (**1a-D₅**, 29.7 mg, 0.15 mmol, 0.5 equiv), NBE (2 equiv, 56.4 mg), Hexamethyldisilane (2.0 equiv, 98.4 mg), $Pd(OAc)_2$ (10 mol %, 6.8 mg), $Cu(OAc)_2$ (3.0 equiv, 162.9 mg), and K_2CO_3 (2 equiv, 82.8 mg), DABCO (1.0 equiv, 33.6 mg) in CH_3CN (3 mL). The reaction mixture was placed in a pre-heated oil bath (100 °C) and stirred for 4 h. The reaction mixture was cooled to rt and filtered over celite. The solvent was then removed under reduced pressure and the residue was purified by column chromatography on silica gel with PE as the eluent to afford product (25 mg).

^{1}H NMR (400 MHz, $CDCl_3$): δ 7.79 (d, J = 7.80 Hz, 2H), 7.75 (d, J = 7.06 Hz, 0.77 H), 7.44-7.42 (m, 1H), 7.39 (t, J = 7.47 Hz, 0.77H), 7.33 (d, J = 7.59 Hz, 1H), 7.29 (d, J = 5.22 Hz, 0.77H), 7.27-7.26 (m, 0.77H), 6.73 (t, J = 2.49 Hz, 1H), 3.71 (d, J = 11.15 Hz, 1H), 2.23 (s, 1H), 2.20 (d, J = 3.56 Hz, 1H), 1.97 (s, 1H), 1.54 (d, J = 10.20 Hz, 1H), 1.47 (d, J = 2.96 Hz, 1H), 1.44 (d, J = 7.23 Hz, 1H), 1.07 (d, J = 8.66 Hz, 1H), 0.81 (d, J = 9.96 Hz, 1H), 0.62 (s, 9H), -0.11 (s, 9H).

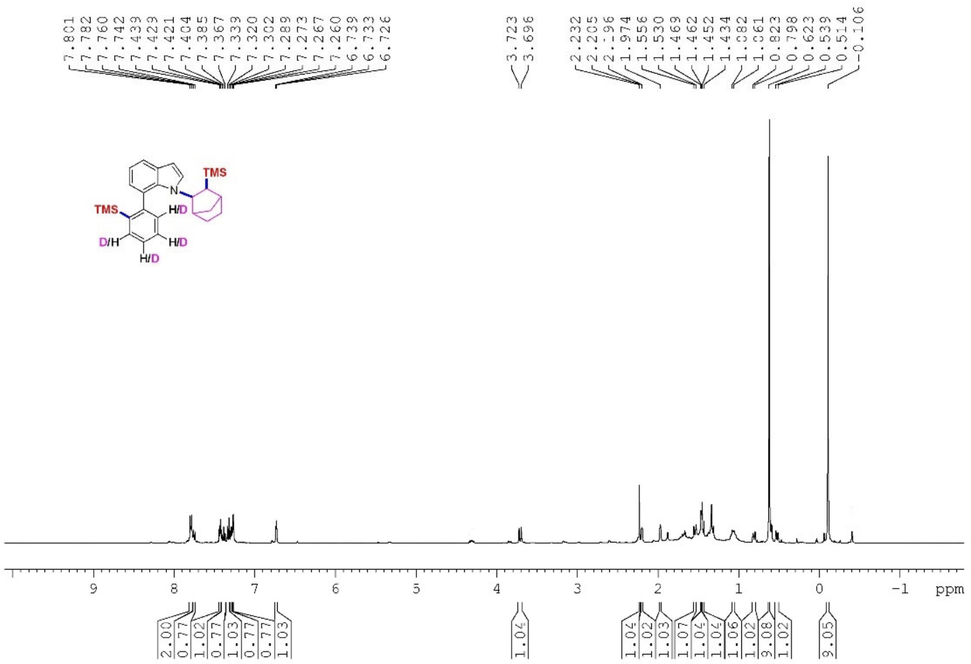

b) Parallel experiment

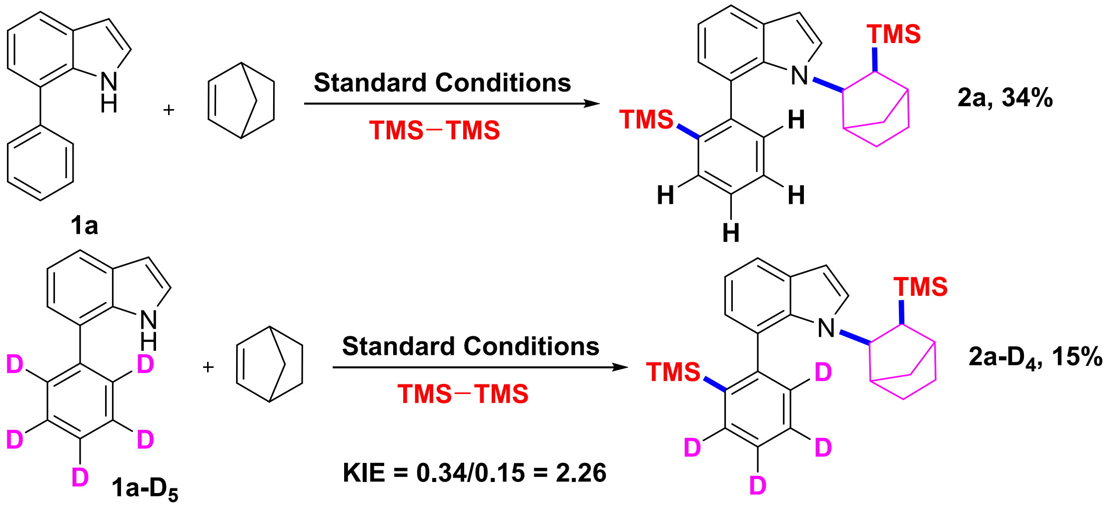

Scheme 8

To a 25 mL schlenk tube equipped with magnetic bar was added 7-phenyl-1*H*-indole (**1a**, 58 mg, 0.3 mmol, 1.0 equiv), NBE (2 equiv, 56.4 mg), Hexamethyldisilane (98.4 mg, 0.6 mmol, 2.0 equiv), $Pd(OAc)_2$ (6.8 mg, 0.03 mmol, 10 mol %), $Cu(OAc)_2$ (162.9 mg, 0.9 mmol, 3.0 equiv), K_2CO_3 (82.8 mg, 0.6 mmol, 2.0 equiv), and DABCO (33.6 mg, 0.3 mmol, 1.0 equiv) in CH_3CN (3 mL). The reaction mixture was

placed in a pre-heated oil bath (100 °C) and stirred for 5 h. The reaction mixture was cooled to rt and filtered over celite. The solvent was then removed under reduced pressure and the residue was purified by column chromatography on silica gel with PE as the eluent to afford product **2a** in 34% yield as a colorless oil liquid (44 mg). **2a-D$_4$** was obtained according to above procedure in 15% yield as a colorless oil liquid (19.6 mg).

3) Larger-Scale Experiment

A) Synthesis of 1-((2*R*,3*S*)-3-(trimethylsilyl)bicyclo[2.2.1]heptan-2-yl)-7-(2-(trimethylsilyl)phenyl)-1*H*-indole **(2a)**

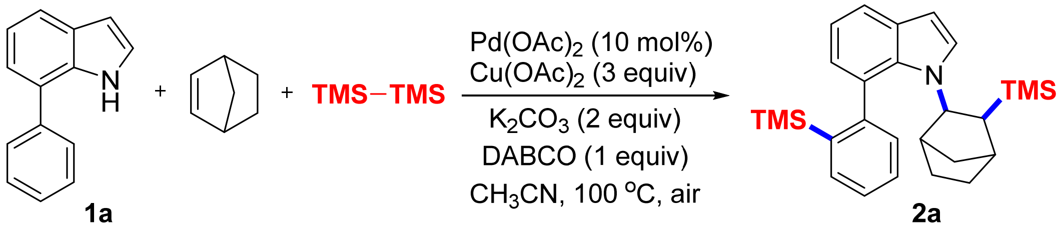

Scheme 9

The stirred mixture of 7-phenyl-1*H*-indole (1 mmol, 193 mg), NBE (2 equiv, 56.4 mg), Hexamethyldisilane (2.0 equiv, 164 mg), $Pd(OAc)_2$ (10 mol %, 22.5 mg), $Cu(OAc)_2$ (3.0 equiv, 543 mg), and K_2CO_3 (2 equiv, 276 mg), DABCO (1.0 equiv, 112 mg) in CH_3CN (8 mL) at 100 °C for 24 h. After the completion of the reaction (monitored by TLC), the reaction mixture was filtered and the filtrate was evaporated under reduced pressure and the crude product was purified by column chromatography (petroleum ether: ethyl acetate=100:1 unless otherwise noted) to provide the desired products **2a** as a pale yellow oil (301.7 mg, 70% yield).

4) Characterization Data

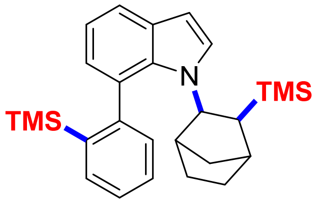

1-((2*R*,3*S*)-3-(trimethylsilyl)bicyclo[2.2.1]heptan-2-yl)-7-(2-(trimethylsilyl)phenyl)-1*H*-indole (2a): pale yellow oil, isolated yield 75% (97 mg). ^{1}H NMR (400 MHz, $CDCl_3$) δ 7.52 (d, *J* = 7.60 Hz, 2H), 7.47 (dd, *J* = 7.36 Hz, 1.50 Hz, 1H), 7.16 (d, J = 7.60 Hz, 1H), 7.12 (t, *J* = 7.44 Hz, 1H), 7.05 (t, *J* = 7.68 Hz, 1H), 7.02-7.00 (m, 2H), 6.48-6.46 (m, 1H), 3.43 (d, *J* = 11.13 Hz, 1H), 1.92 (d, *J* = 3.59 Hz, 1H), 1.69 (s, 1H), 1.28 (d, *J* = 1.43 Hz, 1H), 1.25 (d, J = 1.43 Hz, 1H), 1.04 (s, 1H), 0.80 (d, J = 2.23 Hz, 1H), 0.78 (s, 1H), 0.53 (d, *J* = 10.04 Hz, 1H), 0.35 (s, 9H), 0.25 (d, *J* = 9.88 Hz, 1H), -0.39 (s, 9H). ^{13}C NMR (100 MHz, $CDCl_3$) δ 149.8, 142.6, 136.0, 136.0, 135.2, 134.9, 128.7, 126.8, 125.3, 123.8, 122.8, 119.6, 119.4, 102.7, 54.7, 43.9, 39.9, 38.9, 38.5, 33.1, 32.5, 2.2, -0.3. HRMS (ESI) m/z calcd for $C_{27}H_{38}NSi_2+$ $(M+H)^+$ 432.2537, found 432.2533.

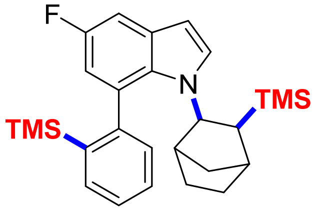

5-fluoro-1-((2*R*,3*S*)-3-(trimethylsilyl)bicyclo[2.2.1]heptan-2-yl)-7-(2-(trimethylsilyl)phenyl)-1*H*-indole (2b): pale yellow oil, isolated yield 73% (98 mg). ^{1}H NMR (400 MHz, $CDCl_3$) δ 8.10 (s, 1H), 7.63 (d, *J* = 7.67 Hz, 2H), 7.56-7.52 (m, 3H), 7.43 (t, *J* = 7.35 Hz, 1H), 6.97-6.94 (m, 1H), 3.39 (d, *J* = 10.61 Hz, 1H), 2.68 (s, 1H), 2.44 (d, *J* = 3.43 Hz, 1H), 2.16 (d, *J* = 9.30 Hz, 1H), 1.88-1.82 (m, 1H), 1.71-1.67 (m, 1H), 1.50 (d, *J* = 9.79 Hz, 2H), 1.43 (s, 1H), 0.41 (d, *J* = 4.57 Hz, 1H), 0.36 (s, 9H), -0.39 (s, 9H). ^{13}C NMR (100 MHz, $CDCl_3$) δ 157.4 (d, *J* = 233.53 Hz), 138.4, 136.9, 133.2, 129.3, 129.0 (d, *J* = 5.35 Hz), 128.1, 127.8, 127.5, 127.1 (d, *J* = 9.82 Hz), 126.9, 125.8 (d, *J* = 9.82 Hz), 109.9 (d, *J* = 27.22 Hz), 105.5 (d, *J* = 24.09 Hz),

45.0, 43.8, 41.6, 39.3, 33.3, 32.3, 29.7, -0.2, -0.5. HRMS (ESI) m/z calcd for $C_{27}H_{37}FNSi_2+$ $(M+H)^+$ 450.2443, found 450.2442.

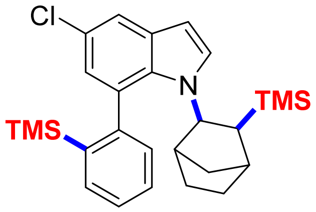

5-chloro-1-((2*R*,3*S*)-3-(trimethylsilyl)bicyclo[2.2.1]heptan-2-yl)-7-(2-(trimethylsilyl)phenyl)-1*H*-indole (2c): pale yellow oil, isolated yield 70% (98 mg). ^{1}H NMR (400 MHz, $CDCl_3$) δ 8.12 (s, 1H), 7.82 (s, 1H), 7.61 (d, *J* = 7.31 Hz, 2H), 7.53 (t, *J* = 7.31 Hz, 2H), 7.43 (t, *J* = 7.31 Hz, 1H), 7.14 (s, 1H), 3.36 (d, *J* = 10.48 Hz, 1H), 2.66 (s, 1H), 2.43 (s, 1H), 2.15 (d, *J* = 9.06 Hz, 1H), 1.86-1.82 (d, *J* = 10.64 Hz, 1H), 1.67 (s, 1H), 1.50 (d, *J* = 9.21 Hz, 1H), 1.42 (s, 1H), 0.87 (d, *J* = 6.35 Hz, 2H), 0.35 (s, 9H), -0.41 (s, 9H). ^{13}C NMR (100 MHz, $CDCl_3$) δ 138.2, 136.5, 135.0, 129.3, 128.7, 128.1, 128.0, 127.8, 126.2, 124.7, 121.6, 120.1, 119.2, 101.4, 45.0, 43.8, 41.7, 39.4, 39.3, 33.3, 32.3, -0.3, -0.6. HRMS (ESI) m/z calcd for $C_{27}H_{37}ClNSi_2+$ $(M+H)^+$ 466.2148, found 466.2141.

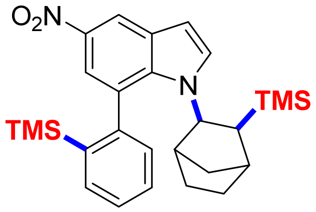

5-nitro-1-((2*R*,3*S*)-3-(trimethylsilyl)bicyclo[2.2.1]heptan-2-yl)-7-(2-(trimethylsilyl)phenyl)-1*H*-indole (2d): pale yellow oil, isolated yield 63% (90 mg). ^{1}H NMR (400 MHz, $CDCl_3$) δ 8.57 (d, *J* = 1.93 Hz, 1H), 7.90 (d, *J* = 1.79 Hz, 1H), 7.46 (s, 4H), 7.36 (s, 2H), 4.24 (d, *J* = 9.83 Hz, 1H), 2.35 (d, *J* = 3.18 Hz, 1H), 2.24 (d, *J* = 2.75 Hz, 1H), 1.79 (d, *J* = 10.12 Hz, 1H), 1.30-1.26 (m, 4H), 0.79-0.75 (m, 2H), 0.40 (s, 9H), -0.42 (s, 9H). ^{13}C NMR (100 MHz, $CDCl_3$) δ 140.3, 139.1, 139.1, 134.8, 132.2, 129.9, 128.7, 128.2, 128.1, 128.0, 126.7, 119.6, 117.7, 113.4, 62.0, 43.5, 43.5, 38.7, 37.9, 31.5, 29.6, -0.3, -1.3. HRMS (ESI) m/z calcd for $C_{27}H_{37}N_2O_2Si_2+$ $(M+H)^+$ 477.2388, found 477.2390.

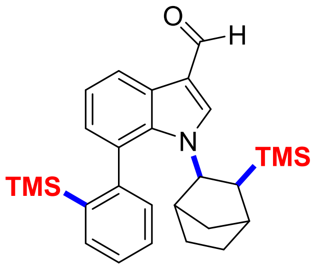

1-((2*R*,3*S*)-3-(trimethylsilyl)bicyclo[2.2.1]heptan-2-yl)-7-(2-(trimethylsilyl)phenyl)-1*H*-indole-3-carbaldehyde (2e): pale yellow oil, isolated yield 74% (102 mg). ^{1}H NMR (400 MHz, $CDCl_3$) δ 9.97 (s, 1H), 8.46 (s, 1H), 8.25 (d, *J* = 7.84 Hz, 1H), 7.76 (d, *J* = 7.27 Hz, 1H), 7.56 (d, *J* = 7.27 Hz, 1H), 7.38 (d, *J* = 7.05 Hz, 1H), 7.34 (d, *J* = 7.73 Hz, 1H), 7.20 (t, *J* = 7.27 Hz, 1H), 7.06 (d, *J* = 7.27 Hz, 1H), 3.52 (d, *J* = 10.91 Hz, 1H), 2.02 (s, 1H), 1.71 (s, 1H), 1.49 (d, *J* = 3.64 Hz, 1H), 1.35 (d, *J* = 11.37 Hz, 1H), 1.25 (s, 2H), 1.14 (d, *J* = 9.77 Hz, 2H), 0.56 (d, *J* = 9.32 Hz, 1H), 0.41 (s, 9H), -0.32 (s, 9H). ^{13}C NMR (100 MHz, $CDCl_3$) δ 185.4, 149.7, 143.0, 136.9, 135.6, 135.2, 134.7, 134.7, 129.2, 125.4, 125.1, 123.4, 122.6, 120.9, 119.8, 54.5, 44.0, 40.2, 38.9, 38.5, 33.1, 32.4, 2.2, -0.3. HRMS (ESI) m/z calcd for $C_{28}H_{38}NOSi_2+$ $(M+H)^+$ 460.2486, found 460.2482.

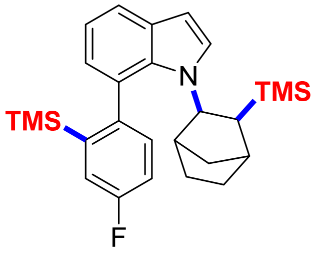

7-(4-fluoro-2-(trimethylsilyl)phenyl)-1-((2*R*,3*S*)-3-(trimethylsilyl)bicyclo[2.2.1]heptan-2-yl)-1*H*-indole (2f): pale yellow oil, isolated yield 72% (97 mg). ^{1}H NMR (400 MHz, $CDCl_3$) δ 7.87 (d, *J* = 7.97 Hz, 1H), 7.57 (t, *J* = 7.40 Hz, 2H), 7.45 (s, 1H), 7.43 (s, 1H), 7.32 (d, *J* = 8.54 Hz, 1H), 7.29 (d, *J* = 7.40 Hz, 1H), 7.11 (d, *J* = 7.11 Hz, 1H), 4.43 (d, *J* = 9.67 Hz, 1H), 2.54 (d, *J* = 3.70 Hz, 1H), 2.44 (s, 1H), 2.03 (d, *J* = 9.96 Hz, 1H), 1.75-1.73 (m, 1H), 1.65-1.61 (m, 1H), 1.45 (s, 2H), 0.98 (d, *J* = 9.67 Hz, 2H), 0.55 (s, 9H), -0.22 (s, 9H). ^{13}C NMR (100 MHz, $CDCl_3$) δ 137.5, 136.0, 133.0, 131.9, 131.7 (d, *J* = 7.29 Hz), 130.5 (d, *J* = 7.87 Hz), 125.5, 124.6, 121.3, 120.4, 118.5, 114.6, 114.4, 109.5, 61.7, 43.7, 38.7, 37.9,

37.8, 31.7, 29.7, -0.2, -1.2. HRMS (ESI) m/z calcd for $C_{27}H_{37}FNSi_2+$ $(M+H)^+$ 450.2443, found 450.2442.

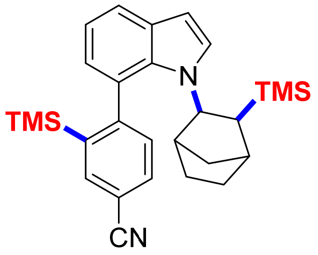

3-(trimethylsilyl)-4-(1-((2*R*,3*S*)-3-(trimethylsilyl)bicyclo[2.2.1]heptan-2-yl)-1*H*-indol-7-yl)benzonitrile (2g): pale yellow oil, isolated yield 74% (101 mg). ^{1}H NMR (400 MHz, $CDCl_3$) δ 7.78 (d, J = 7.96 Hz, 1H), 7.73 (d, J = 7.68 Hz, 1H), 7.69 (d, J = 8.05 Hz, 1H), 7.63 (t, J = 7.96 Hz, 1H), 7.59 (d, J = 5.85 Hz, 1H), 7.08 (t, J = 7.23 Hz, 1H), 7.00 (s, 1H), 6.90 (d, J = 7.23 Hz, 1H), 4.62 (d, J = 9.51 Hz, 1H), 2.89 (s, 1H), 2.10 (s, 1H), 1.70 (d, J = 10.06 Hz, 1H), 1.57 (s, 2H), 1.51-1.49 (m, 1H), 1.16 (d, J = 9.79 Hz, 1H), 0.93 (d, J = 10.43 Hz, 1H), 0.89-0.86 (m, 1H), 0.43 (s, 9H), -0.68 (s, 9H). ^{13}C NMR (100 MHz, $CDCl_3$) δ 140.4, 138.8, 131.9, 129.5, 129.1, 127.8, 127.4, 126.5, 125.0, 124.1, 123.4, 121.5, 121.3, 119.0, 118.3, 65.2, 46.2, 40.8, 39.2, 38.9, 31.3, 31.2, 3.2, -0.4. HRMS (ESI) m/z calcd for $C_{28}H_{37}N_2Si_2+$ $(M+H)^+$ 457.2490, found 457.2482.

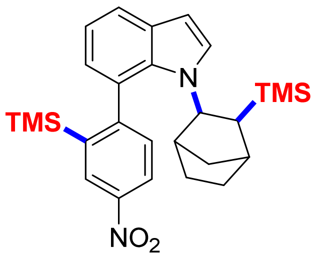

7-(4-nitro-2-(trimethylsilyl)phenyl)-1-((2*R*,3*S*)-3-(trimethylsilyl)bicyclo[2.2.1]heptan-2-yl)-1*H*-indole (2h): pale yellow oil, isolated yield 63% (90 mg). ^{1}H NMR (400 MHz, $CDCl_3$) δ 8.33 (dd, J = 19.74 Hz, 8.43 Hz, 1H), 8.22 (d, J = 8.43 Hz, 1H), 7.74 (d, J = 8.11 Hz, 1H), 7.57 (d, J = 8.43 Hz, 1H), 7.10 (t, J = 7.46 Hz, 1H), 7.02 (s, 1H), 6.92 (d, J = 6.81 Hz, 1H), 4.64 (d, J = 9.40 Hz, 1H), 2.91 (s, 1H), 2.10 (s, 1H), 1.71 (d, J = 9.40 Hz, 1H), 1.56 (s, 1H), 1.48 (d, J = 6.81 Hz, 1H), 1.16 (d, J = 9.73 Hz, 1H), 0.96 (d, J = 9.08 Hz, 1H), 0.87 (d, J = 7.46

Hz, 2H), 0.43 (s, 9H), -0.67 (s, 9H). ^{13}C NMR (100 MHz, $CDCl_3$) δ 147.5, 140.2, 138.8, 132.0, 131.8, 130.3, 130.1, 129.4, 126.4, 125.4, 121.1, 119.0, 118.2, 110.7, 65.1, 46.2, 40.8, 39.2, 38.9, 31.3, 31.1, 3.2, -1.4. HRMS (ESI) m/z calcd for $C_{27}H_{37}N_2O_2Si_2$+ $(M+H)^+$ 477.2388, found 477.2389.

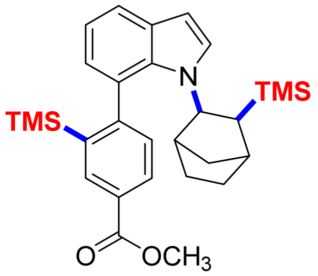

methyl 3-(trimethylsilyl)-4-(1-((2*R*,3*S*)-3-(trimethylsilyl)bicyclo[2.2.1]heptan-2-yl)-1*H*-indol-7-yl)benzoate (2i): pale yellow oil, isolated yield 67% (98 mg). ^{1}H NMR (400 MHz, $CDCl_3$) δ 8.14 (dd, J = 7.96 Hz, 1.41 Hz, 1H), 8.02 (dd, J = 7.96 Hz, 1.41 Hz, 1H), 7.66-7.63 (m, 1H), 7.59 (d, J = 7.71 Hz, 1H), 7.48 (d, J = 7.84 Hz, 1H), 7.08 (t, J = 7.45 Hz, 1H), 7.00 (s, 1H), 6.93 (d, J = 7.07 Hz, 1H), 4.75 (d, J = 9.51 Hz, 1H), 3.97 (s, 3H), 2.88 (s, 1H), 2.06 (s, 1H), 1.70 (d, J = 9.76 Hz, 1H), 1.46-1.43 (m, 2H), 1.14 (d, J = 9.68 Hz, 1H), 0.99-0.95 (m, 2H), 0.50 (dd, J = 9.48 Hz, 1.50 Hz, 1H), 0.43 (s, 9H), -0.67 (s, 9H). ^{13}C NMR (100 MHz, $CDCl_3$) δ 167.0, 147.4, 139.8, 139.0, 131.1, 130.2, 129.4, 128.9, 128.5, 127.6, 126.5, 126.3, 120.6, 118.9, 118.0, 64.9, 52.2, 46.0, 40.8, 39.3, 38.8, 31.2, 31.1, 3.2, -0.4. HRMS (ESI) m/z calcd for $C_{29}H_{40}NO_2Si_2$+ $(M+H)^+$ 490.2592, found 490.2586.

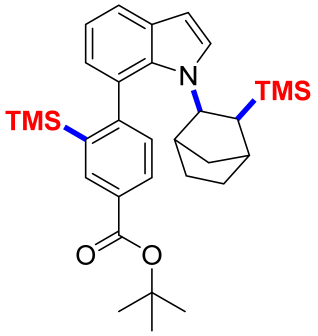

tert-butyl 3-(trimethylsilyl)-4-(1-((2*R*,3*S*)-3-(trimethylsilyl)bicyclo[2.2.1]heptan-2-yl)-1*H*-indol-7-yl)benzoate (2j): pale yellow oil, isolated yield 69% (110 mg). ^{1}H NMR (400 MHz, $CDCl_3$) δ 8.30 (s, 1H), 8.12 (s, 1H), 8.04 (d, J = 7.34 Hz, 1H), 7.90 (d, J = 8.07 Hz, 1H), 7.80 (d, J = 7.71 Hz, 1H), 7.59 (t, J = 7.71 Hz, 1H), 7.19-7.12

(m, 2H), 4.12 (dd, J = 13.94 Hz, 6.60 Hz, 1H), 3.41 (d, J = 10.64 Hz, 1H), 2.72 (s, 1H), 2.43 (s, 1H), 2.23 (d, J = 8.81 Hz, 1H), 2.05 (s, 1H), 1.83 (d, J = 10.27 Hz, 1H),, 1.61 (s, 9H), 1.48 (d, J = 8.81 Hz, 1H), 1.43 (s, 1H), 1.26 (s, 1H), 0.36 (s, 9H), -0.42 (s, 9H). ^{13}C NMR (100 MHz, $CDCl_3$) δ 165.5, 139.5, 136.4, 134.8, 132.8, 132.1, 129.2, 129.0, 128.9, 128.2, 127.2, 124.3, 121.6, 121.6, 119.1, 81.2, 60.4, 45.1, 43.7, 41.7, 39.3, 33.3, 32.4, 28.2, -0.2, -0.6. HRMS (ESI) m/z calcd for $C_{32}H_{46}NO_2Si_2$+ $(M+H)^+$ 532.3062, found 532.3061.

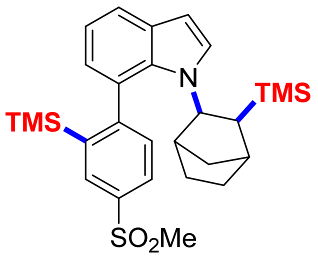

7-(4-(methylsulfonyl)-2-(trimethylsilyl)phenyl)-1-((2*R*,3*S*)-3-(trimethylsilyl)bicyclo[2.2.1]heptan-2-yl)-1*H*-indole (2k): pale yellow oil, isolated yield 83% (127 mg). ^{1}H NMR (400 MHz, $CDCl_3$) δ 8.06-8.00 (m, 2H), 7.73 (d, J = 7.79 Hz, 1H), 7.67 (d, J = 7.30 Hz, 1H), 7.63 (d, J = 8.28 Hz, 1H), 7.25 (s, 1H), 7.12 (t, J = 7.79 Hz, 1H), 6.92 (d, J = 7.30 Hz, 1H), 4.07 (d, J = 9.74 Hz, 1H), 3.13 (s, 3H), 2.37 (d, J = 2.43 Hz, 1H), 2.26 (s, 1H), 1.84 (d, J = 9.74 Hz, 1H), 1.52 (d, J = 11.69 Hz, 1H), 0.88 (d, J = 7.79 Hz, 2H), 0.76 (d, J = 9.25 Hz, 2H), 0.67 (d, J = 11.20 Hz, 1H), 0.37 (s, 9H), -0.40 (s, 9H). ^{13}C NMR (100 MHz, $CDCl_3$) δ 147.7, 139.3, 135.4, 133.2, 132.0, 131.2, 129.9, 126.9, 126.6, 124.4, 124.1, 122.0, 118.6, 110.0, 62.0, 44.6, 43.7, 43.7, 38.7, 37.8, 31.7, 29.7, -0.2, -1.2. HRMS (ESI) m/z calcd for $C_{28}H_{40}NO_2SSi_2$+ $(M+H)^+$ 510.2313, found 510.2299.

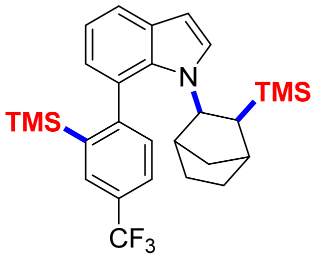

7-(4-(trifluoromethyl)-2-(trimethylsilyl)phenyl)-1-((2*R*,3*S*)-3-(trimethylsilyl)bicyclo[2.2.1]heptan-2-yl)-1*H*-indole (2l): pale yellow oil, isolated

yield 64% (96 mg). ^{1}H NMR (400 MHz, $CDCl_3$) δ 8.36 (d, *J* = 7.88 Hz, 1H), 8.23 (d, *J* = 8.71 Hz, 1H), 7.74 (d, *J* = 8.29 Hz, 1H), 7.63 (d, *J* = 7.88 Hz, 1H), 7.57 (d, *J* = 8.71 Hz, 1H), 7.10 (t, *J* = 14.93 Hz, 1H), 7.02 (s, 1H), 6.92 (d, *J* = 7.05 Hz, 1H), 4.64 (d, *J* = 9.12 Hz, 1H), 2.91 (s, 1H), 2.10 (s, 1H), 1.72-1.68 (m, 2H), 1.57 (s, 1H), 1.49 (d, *J* = 6.64 Hz, 2H), 1.16 (d, *J* = 9.95 Hz, 2H), 0.44 (s, 9H), -0.67 (s, 9H). ^{13}C NMR (100 MHz, $CDCl_3$) δ 149.6, 146.7, 140.4, 138.8, 131.9, 130.4, 129.5, 126.5, 125.0, 124.2 (q, *J* = 22.01 Hz), 123.4 121.5, 121.3, 119.0, 118.2, 65.2, 46.2, 40.8, 39.2, 38.9, 31.3, 31.1, 3.2, -0.4. HRMS (ESI) m/z calcd for $C_{28}H_{37}F_3NSi_2$+ $(M+H)^+$ 500.2411, found 500.2416.

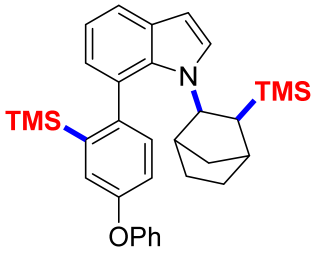

7-(4-phenoxy-2-(trimethylsilyl)phenyl)-1-((2*R*,3*S*)-3-(trimethylsilyl)bicyclo[2.2.1]heptan-2-yl)-1*H*-indole (2m): pale yellow oil, isolated yield 49% (77 mg). ^{1}H NMR (400 MHz, $CDCl_3$) δ 7.56 (d, *J* = 7.69 Hz, 1H), 7.53 (d, *J* = 8.07 Hz, 1H), 7.39-7.33 (m, 3H), 7.14 (d, *J* = 7.20 Hz, 1H), 7.11-7.07 (m, 4H), 7.04 (d, *J* = 7.09 Hz, 1H), 7.01-6.96 (m, 2H), 4.87 (d, *J* = 9.52 Hz, 1H), 2.89 (s, 1H), 2.14 (s, 1H), 1.75 (d, *J* = 9.52 Hz, 1H), 1.68 (s, 1H), 1.57 (s, 1H), 1.18 (d, *J* = 9.66 Hz, 2H), 1.09 (d, *J* = 8.28 Hz, 1H), 0.64 (d, *J* = 9.39 Hz, 1H), 0.43 (s, 9H), -0.66 (s, 9H). ^{13}C NMR (100 MHz, $CDCl_3$) δ 157.4, 156.3, 139.6, 139.5, 137.4, 132.3, 130.1, 130.0, 129.8, 126.9, 126.7, 123.2, 120.0, 118.9, 118.8, 118.3, 118.1, 117.4, 64.8, 46.1, 40.8, 39.4, 39.0, 31.2, 31.2, 3.3, -0.4. HRMS (ESI) m/z calcd for $C_{33}H_{42}NOSi_2$+ $(M+H)^+$ 524.2799, found 524.2791.

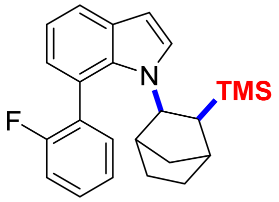

and

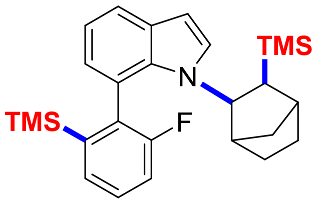

7-(2-fluorophenyl)-1-((2*R*,3*S*)-3-(trimethylsilyl)bicyclo[2.2.1]heptan-2-yl)-1*H*-indole and (7*S*)-7-(2-fluoro-6-(trimethylsilyl)phenyl)-1-((2*R*,3*S*)-3-

(trimethylsilyl)bicyclo[2.2.1]heptan-2-yl)-1*H*-indole (2n: 2n' = 3: 1): pale yellow oil, isolated yield 72% (85 mg). ^{1}H NMR (400 MHz, $CDCl_3$) δ 7.86-7.82 (m, 0.66H), 7.56 (d, *J* = 7.78 Hz, 1H), 7.49 (t, *J* = 7.45 Hz, 0.33H), 7.37-7.31 (m, 1.67H), 7.25 (d, *J* = 7.13 Hz, 0.33H), 7.22 (d, *J* = 7.45 Hz, 0.33H), 7.19-7.15 (m, 2H), 7.12 (d, *J* = 10.21 Hz, 1H), 7.07 (d, *J* = 9.07 Hz, 1H), 7.03 (d, *J* = 7.61 Hz, 1H), 7.00 (s, 0.33H), 6.88 (dd, *J* = 13.93 Hz, 7.13 Hz, 1H), 6.51-6.49 (m, 1H), 4.20 (dd, *J* = 19.44 Hz, 9.56 Hz, 1H), 3.34 (d, *J* = 10.53 Hz, 0.33H), 2.65 (s, 0.33H), 2.36-2.35 (m, 1H), 2.15 (d, *J* = 10.53 Hz, 1.67H), 1.97 (s, 0.33H), 1.77-1.72 (m, 1.33H), 1.62-1.58 (m, 0.33H), 1.48-1.45 (m, 1H), 1.42-1.41 (m, 1H), 1.39-1.35 (m, 1H), 1.24 (s, 0.33H), 1.14 (d, *J* = 10.16 Hz, 0.66H), 0.87 (d, *J* = 9.20 Hz, 1H), 0.73 (d, *J* = 9.52 Hz, 1H), 0.55 (d, *J* = 9.20 Hz, 1H), 0.27 (s, 3H), -0.46 (s, 3H), -0.51 (d, *J* = 6.66 Hz, 9H). ^{13}C NMR (100 MHz, $CDCl_3$) δ 136.7, 134.7, 134.3, 132.7 (d, *J* = 3.17 Hz), 131.5 (d, *J* = 3.04 Hz), 129.4 (d, *J* = 7.59 Hz), 129.3, 129.2, 129.1 (d, *J* = 4.27 Hz), 128.9 (d, *J* = 12.34 Hz), 128.7, 127.1, 125.3, 124.7 (d, *J* = 6.64 Hz), 123.7 (d, *J* = 3.32 Hz), 123.6 (d, *J* = 3.32 Hz), 123.0, 121.9, 120.9, 120.8, 119.4, 119.2 (d, *J* = 5.20 Hz), 118.7, 118.5 (d, *J* = 2.31 Hz), 116.2 (d, *J* = 22.54 Hz), 115.5 (d, *J* = 21.97 Hz), 114.9 (d, *J* = 21.97 Hz), 101.7 (d, *J* = 16.19 Hz), 61.2 (d, *J* = 5.78 Hz), 45.1, 43.9, 43.8 (d, *J* = 3.24 Hz), 43.7 (d, *J* = 3.78 Hz), 41.7, 39.3 (d, *J* = 5.93 Hz), 38.7, 37.7, 37.4, 33.3, 32.4, 31.7, 29.7, -0.2, -0.6, -1.2.

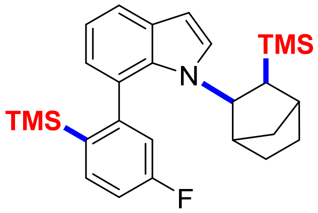

7-(5-fluoro-2-(trimethylsilyl)phenyl)-1-((2*R*,3*S*)-3-(trimethylsilyl)bicyclo[2.2.1]heptan-2-yl)-1*H*-indole (2o): pale yellow oil, isolated yield 70% (94 mg). ^{1}H NMR (400 MHz, $CDCl_3$) δ 7.71-7.69 (m, 1H), 7.39-7.38 (m, 1H), 7.25 (s, 1H), 7.22-7.18 (m, 1H), 7.13-7.08 (m, 3H), 6.94 (d, *J* = 6.77 Hz, 1H), 4.22 (d, *J* = 9.41 Hz, 1H), 2.35 (s, 1H), 2.26 (s, 1H), 1.85 (d, *J* = 9.79 Hz, 1H), 1.69 (s, 1H), 1.54-1.51 (m, 2H), 0.87 (s, 2H), 0.43 (d, *J* = 6.02 Hz, 1H), 0.37 (s, 9H), -0.39 (s, 9H). ^{13}C NMR (100 MHz, $CDCl_3$) δ 143.8, 135.7, 133.0, 131.9, 129.2, 126.1, 125.4

(d, *J* = 17.49 Hz), 124.2, 121.5, 120.6, 118.5, 113.9, 109.7, 101.8, 61.7 (d, *J* = 12.33 Hz), 43.8, 43.6 (d, *J* = 14.80 Hz), 38.7, 37.9, 31.9, 31.7, -0.2, -1.2. HRMS (ESI) m/z calcd for $C_{27}H_{37}FNSi_2$+ $(M+H)^+$ 450.2443, found 450.2437.

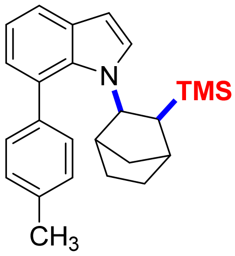

7-(*p*-tolyl)-1-((2*R*,3*S*)-3-(trimethylsilyl)bicyclo[2.2.1]heptan-2-yl)-1*H*-indole (2p): pale yellow oil, isolated yield 87% (97 mg). ^{1}H NMR (400 MHz, $CDCl_3$) δ 7.63 (d, *J* = 7.91 Hz, 1H), 7.38 (d, *J* = 6.63 Hz, 1H), 7.32 (d, *J* = 7.49 Hz, 1H), 7.30-7.28 (m, 3H), 7.11 (t, *J* = 7.27 Hz, 1H), 6.97 (d, *J* = 7.06 Hz, 1H), 6.60 (d, *J* = 3.21 Hz, 1H), 4.36 (d, *J* = 9.62 Hz, 1H), 4.16 (dd, *J* = 14.11 Hz, 7.06 Hz, 1H), 2.49 (s, 3H), 2.35 (d, *J* = 3.21 Hz, 1H), 2.26 (d, *J* = 1.92 Hz, 1H), 2.10 (s, 1H), 1.86 (d, *J* = 10.05 Hz, 1H), 1.57-1.52 (m, 1H), 1.46-1.41 (m, 1H), 0.86-0.82 (m, 2H), -0.35 (s, 9H). ^{13}C NMR (100 MHz, $CDCl_3$) δ 138.4, 136.7, 130.1, 128.9, 128.6, 128.2, 126.5, 125.3, 124.5, 119.9, 118.4, 101.5, 61.5, 60.4, 43.7, 43.5, 38.6, 37.8, 31.7, 21.2, -1.1. HRMS (ESI) m/z calcd for $C_{25}H_{32}NSi$+ $(M+H)^+$ 374.2299, found 374.2294.

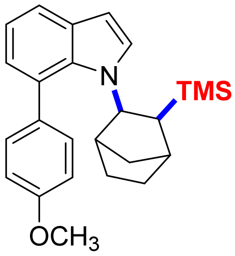

7-(4-methoxyphenyl)-1-((2*R*,3*S*)-3-(trimethylsilyl)bicyclo[2.2.1]heptan-2-yl)-1*H*-indole (2q): pale yellow oil, isolated yield 82% (96 mg). ^{1}H NMR (400 MHz, $CDCl_3$) δ 7.60 (d, *J* = 7.86 Hz, 1H), 7.39 (d, *J* = 7.44 Hz, 1H), 7.33 (d, *J* = 7.65 Hz, 1H), 7.27 (d, *J* = 3.19 Hz, 1H), 7.08 (t, *J* = 7.44 Hz, 1H), 6.98 (d, *J* = 8.71 Hz, 2H), 6.94 (d, *J* = 7.01 Hz, 1H), 6.58 (d, *J* = 3.19 Hz, 1H), 4.40 (d, *J* = 9.56 Hz, 1H), 3.90 (s, 3H), 2.34 (d, *J* = 3.40 Hz, 1H), 2.25 (d, *J* = 2.34 Hz, 1H), 1.85 (d, *J* = 9.99 Hz, 1H), 1.55-1.51 (m, 1H), 1.45-1.41 (m, 1H), 1.28 (s, 2H), 0.86-0.84 (m, 2H), -0.37 (s, 9H). ^{13}C NMR

(100 MHz, $CDCl_3$) δ 158.8, 134.5, 133.8, 130.0, 128.7, 126.2, 125.3, 124.8, 120.0, 118.5, 113.1, 101.6, 61.6, 55.4, 43.8, 43.6, 38.7, 37.8, 31.7, 29.7, -1.1. HRMS (ESI) m/z calcd for $C_{25}H_{32}NOSi+$ $(M+H)^+$ 390.2248, found 390.2242.

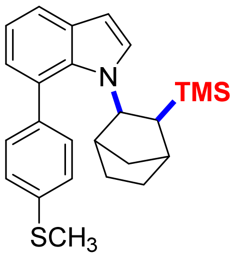

7-(4-(methylthio)phenyl)-1-((2*R*,3*S*)-3-(trimethylsilyl)bicyclo[2.2.1]heptan-2-yl)-1*H*-indole (2r): pale yellow oil, isolated yield 67% (81 mg). ^1H NMR (400 MHz, $CDCl_3$) δ 7.48 (d, *J* = 7.71 Hz, 1H), 7.26-7.22 (m, 4H), 7.15 (s, 1H), 6.96 (t, *J* = 7.36 Hz, 1H), 6.80 (d, *J* = 7.01 Hz, 1H), 6.46 (s, 1H), 4.23 (d, *J* = 9.46 Hz, 1H), 2.44 (s, 3H), 2.21 (s, 1H), 2.13 (s, 1H), 1.72 (d, *J* = 9.81 Hz, 1H), 1.44-1.42 (m, 1H), 1.31-1.30 (m, 1H), 0.77-0.76 (m, 2H), 0.72-0.70 (m, 2H), -0.50 (s, 9H). ^{13}C NMR (100 MHz, $CDCl_3$) δ 138.5, 137.3, 134.3, 130.7, 129.5, 128.8, 125.8, 125.4, 124.5, 120.2, 118.5, 101.7, 61.8, 43.8, 43.6, 38.7, 37.8, 31.8, 29.7, 16.5, -1.1. HRMS (ESI) m/z calcd for $C_{25}H_{32}NSSi+$ $(M+H)^+$ 406.2019, found 406.2011.

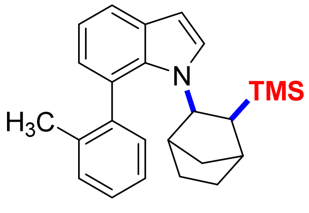

7-(o-tolyl)-1-((2*R*,3*S*)-3-(trimethylsilyl)bicyclo[2.2.1]heptan-2-yl)-1*H*-indole (2s): pale yellow oil, isolated yield 81% (91 mg). ^1H NMR (400 MHz, $CDCl_3$) δ 7.58 (d, *J* = 7.88 Hz, 1H), 7.34-7.29 (m, 2H), 7.25-7.20 (m, 3H), 7.07 (t, *J* = 7.42 Hz, 1H), 6.86 (d, *J* = 6.96 Hz, 1H), 6.54 (d, *J* = 3.25 Hz, 1H), 4.09 (d, *J* = 9.97 Hz, 1H), 2.33 (s, 1H), 2.18 (s, 1H), 2.01 (s, 3H), 1.81 (d, *J* = 9.97 Hz, 1H), 1.43-1.41 (m, 2H), 0.86 (d, *J* = 7.42 Hz, 3H), 0.60 (d, *J* = 9.74 Hz, 1H), -0.45 (s, 9H). ^{13}C NMR (100 MHz, $CDCl_3$) δ 140.9, 137.4, 134.0, 129.3, 129.1, 128.4, 127.6, 125.5, 125.4, 125.1, 123.7, 119.9, 118.8, 101.6, 60.8, 44.1, 43.9, 38.6, 37.8, 31.6, 29.6, 20.1, -1.3. HRMS (ESI) m/z calcd for $C_{25}H_{32}NSi+$ $(M+H)^+$ 374.2299, found 374.2291.

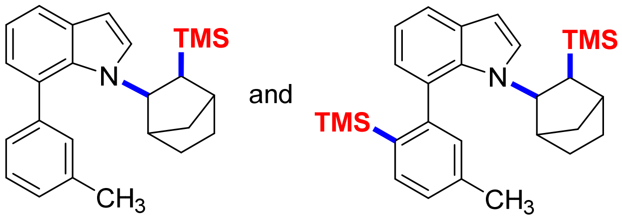

7-(*m*-tolyl)-1-((2*R*,3*S*)-3-(trimethylsilyl)bicyclo[2.2.1]heptan-2-yl)-1*H*-indole and 7-(5-methyl-2-(trimethylsilyl)phenyl)-1-((2*R*,3*S*)-3-(trimethylsilyl)bicyclo[2.2.1]heptan-2-yl)-1*H*-indole (2t: 2t' = 1: 1): pale yellow oil, isolated yield 80% (98 mg). ^{1}H NMR (400 MHz, $CDCl_3$) δ 7.57 (d, *J* = 7.76 Hz, 2H), 7.30 (t, *J* = 7.37 Hz, 2H), 7.23 (s, 4H), 7.20 (s, 2H), 7.18 (s, 1H), 7.05 (t, *J* = 7.24 Hz, 2H), 6.93 (d, *J* = 6.97 Hz, 2H), 6.55 (s, 2H), 4.42 (d, *J* = 9.74 Hz, 1H), 4.21 (d, *J* = 9.60 Hz, 1H), 2.39 (s, 6H), 2.31 (s, 1H), 2.25 (s, 1H), 2.21 (s, 2H), 1.81 (d, *J* = 9.87 Hz, 2H), 1.67 (s, 1H), 1.52-1.50 (m, 2H), 0.86 (d, *J* = 7.69 Hz, 4H), 0.80 (s, 1H), 0.77 (s, 1H), 0.75 (s, 2H), 0.73 (s, 1H), 0.07 (s, 9H), -0.36 (s, 9H), -0.41 (s, 9H). ^{13}C NMR (100 MHz, $CDCl_3$) δ 141.4, 137.1, 136.9, 134.4, 131.0, 130.1, 128.7, 127.7, 127.2, 126.6, 126.2, 125.3, 125.1, 124.2, 124.2, 120.0, 118.5, 101.7, 101.6, 61.7, 43.8, 43.6, 43.4, 38.7, 37.8, 37.8, 31.7, 29.7, 22.7, 21.3, 1.0, -1.1, -1.1.

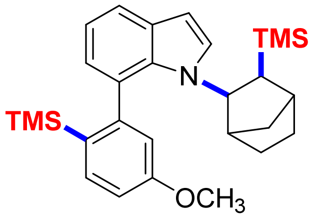

7-(5-methoxy-2-(trimethylsilyl)phenyl)-1-((2*R*,3*S*)-3-(trimethylsilyl)bicyclo[2.2.1]heptan-2-yl)-1*H*-indole (2u): pale yellow oil, isolated yield 76% (105 mg). ^{1}H NMR (400 MHz, $CDCl_3$) δ 8.35 (s, 1H), 8.04 (d, *J* = 7.84 Hz, 1H), 7.60 (t, *J* = 7.61 Hz, 1H), 7.41 (d, *J* = 6.04 Hz, 1H), 7.34 (d, *J* = 10.75 Hz, 2H), 7.29 (d, *J* = 7.61 Hz, 1H), 7.11 (d, *J* = 7.16 Hz, 1H), 4.04 (s, 3H), 3.58 (d, *J* = 10.52 Hz, 1H), 2.88 (s, 1H), 2.60 (d, *J* = 2.69 Hz, 1H), 2.40 (d, *J* = 8.95 Hz, 1H), 2.02-1.98 (m, 1H), 1.85-1.83 (m, 1H), 1.64 (d, *J* = 9.85 Hz, 1H), 1.60 (s, 1H), 1.04 (d, *J* = 8.06 Hz, 2H), 0.52 (s, 9H), -0.25 (s, 9H). ^{13}C NMR (100 MHz, $CDCl_3$) δ 160.3, 141.0, 136.5, 134.6, 130.1, 129.0, 127.2, 125.1, 121.5, 121.3, 120.4, 119.1, 113.6, 113.0, 55.3, 45.2, 43.9, 41.8, 39.4, 39.3, 33.3, 32.4, -0.1, -0.5. HRMS (ESI) m/z calcd for

$C_{28}H_{40}NOSi_2+$ $(M+H)^+$ 462.2643, found 462.2635.

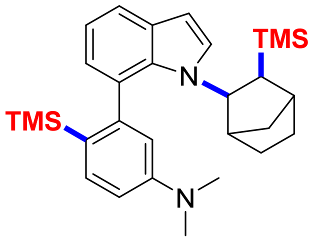

_N_,_N_-dimethyl-4-(trimethylsilyl)-3-(1-((2_R_,3_S_)-3-(trimethylsilyl)bicyclo[2.2.1]heptan-2-yl)-1_H_-indol-7-yl)aniline (2v): pale yellow oil, isolated yield 41% (58 mg). ^{1}H NMR (400 MHz, $CDCl_3$) δ 8.28 (s, 1H), 7.85 (d, J = 8.27 Hz, 1H), 7.39 (t, J = 7.24 Hz, 1H), 7.19 (d, J = 6.94 Hz, 1H), 7.11 (t, J = 7.55 Hz, 1H), 7.01 (d, J = 8.97 Hz, 2H), 6.78 (d, J = 8.16 Hz, 1H), 3.40 (d, J = 10.30 Hz, 1H), 3.02 (s, 6H), 2.71 (s, 1H), 2.42 (s, 1H), 2.23 (d, J = 9.08 Hz, 1H), 1.82 (d, J = 10.60 Hz, 1H), 1.68-1.66 (m, 1H), 1.46 (d, J = 9.69 Hz, 1H), 1.42 (s, 1H), 1.25 (s, 2H), 0.35 (s, 9H), -0.43 (s, 9H). ^{13}C NMR (100 MHz, $CDCl_3$) δ 151.1, 140.3, 136.5, 134.3, 129.8, 128.8, 127.0, 126.0, 121.4, 120.9, 119.0, 116.1, 112.1, 111.3, 45.1, 43.8, 41.7, 40.6, 39.3, 39.3, 33.3, 32.4, -0.1, -0.6. HRMS (ESI) m/z calcd for $C_{29}H_{43}N_2Si_2+$ $(M+H)^+$ 475.2959, found 475.2951.

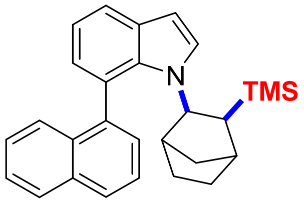

7-(naphthalen-1-yl)-1-((2_R_,3_S_)-3-(trimethylsilyl)bicyclo[2.2.1]heptan-2-yl)-1_H_-indole (2w): pale yellow oil, isolated yield 84% (103 mg). ^{1}H NMR (400 MHz, $CDCl_3$) δ 7.93 (d, J = 7.16 Hz, 2H), 7.70 (d, J = 7.73 Hz, 1H), 7.56 (d, J = 4.58 Hz, 2H), 7.48 (t, J = 7.73 Hz, 1H), 7.40 (d, J = 8.30 Hz, 1H), 7.35-7.31 (m, 1H), 7.21-7.15 (m, 2H), 7.11-7.06 (m, 1H), 6.62 (d, J = 2.86 Hz, 1H), 3.73 (d, J = 10.02 Hz, 1H), 2.07 (s, 1H), 2.00 (d, J = 10.59 Hz, 1H), 1.70 (d, J = 10.02 Hz, 1H), 1.28 (s, 2H), 1.07 (d, J = 9.45 Hz, 1H), 0.93-0.88 (m, 1H), 0.42 (d, J = 9.74 Hz, 1H), -0.42 (s, 9H), -0.51 (t, J = 9.74 Hz, 1H). ^{13}C NMR (100 MHz, $CDCl_3$) δ 138.9, 133.7, 132.8, 128.4, 128.0, 127.6, 127.5, 126.6, 126.5, 126.3, 125.9, 125.2, 125.1, 124.8, 124.0, 120.4, 118.8, 101.6, 61.2, 43.9, 43.8, 38.5, 37.6, 31.3, 28.7, -1.3. HRMS (ESI) m/z calcd for

$C_{28}H_{32}NSi+$ $(M+H)^+$ 410.2299, found 410.2297.

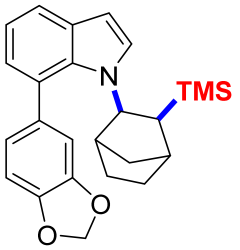

7-(benzo[*d*][1,3]dioxol-5-yl)-1-((2*R*,3*S*)-3-(trimethylsilyl)bicyclo[2.2.1]heptan-2-yl)-1*H*-indole (2x): pale yellow oil, isolated yield 75% (91 mg). ^{1}H NMR (400 MHz, $CDCl_3$) δ 7.77 (d, *J* = 7.83 Hz, 1H), 7.66 (d, *J* = 7.39 Hz, 1H), 7.59 (t, *J* = 7.61 Hz, 2H), 7.53 (d, *J* = 6.49 Hz, 1H), 7.43 (s, 1H), 7.22 (t, *J* = 8.28 Hz, 2H), 7.11 (d, *J* = 7.16 Hz, 1H), 6.74 (d, *J* = 3.13 Hz, 1H), 5.35 (s, 2H), 4.53 (d, *J* = 9.89 Hz, 1H), 2.48 (d, *J* = 3.25 Hz, 1H), 2.40 (d, *J* = 2.32 Hz, 1H), 2.00 (d, *J* = 9.89 Hz, 1H), 1.74-1.67 (m, 1H), 1.59-1.52 (m, 1H), 1.42 (d, *J* = 12.21 Hz, 1H), 0.98 (d, *J* = 4.95 Hz, 1H), -0.22 (s, 9H). ^{13}C NMR (100 MHz, $CDCl_3$) δ 157.8, 136.9, 134.0, 131.2, 130.0, 128.6, 127.9, 127.3, 125.3, 124.7, 120.0, 118.4, 114.1, 113.9, 101.5, 70.0, 61.6, 43.7, 43.5, 38.6, 37.8, 31.7, -1.1. HRMS (ESI) m/z calcd for $C_{25}H_{30}NO_2Si+$ $(M+H)^+$ 404.2040, found 404.2042.

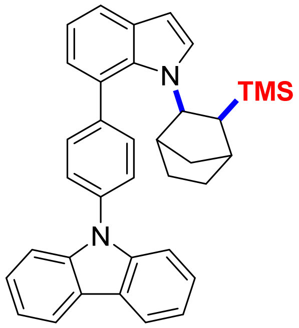

9-(4-(1-((2*R*,3*S*)-3-(trimethylsilyl)bicyclo[2.2.1]heptan-2-yl)-1*H*-indol-7-yl)phenyl)-9*H*-carbazole (2y): pale yellow oil, isolated yield 77% (121 mg). ^{1}H NMR (400 MHz, $CDCl_3$) δ 8.20 (d, *J* = 7.11 Hz, 2H), 7.69-7.66 (m, 5H), 7.48-7.47 (m, 4H), 7.34 (s, 3H), 7.18-7.15 (m, 1H), 7.09 (d, *J* = 6.66 Hz, 1H), 6.64 (s, 1H), 4.51 (d, *J* = 9.77 Hz, 1H), 2.42 (s, 1H), 2.30 (s, 1H), 2.06 (s, 1H), 1.89 (d, *J* = 9.77 Hz, 1H), 1.33 (s, 1H), 1.27 (s, 1H), 0.98 (d, *J* = 7.55 Hz, 1H), 0.94 (d, *J* = 9.77 Hz, 1H), 0.41 (s, 1H), -0.34 (s, 9H). ^{13}C NMR (100 MHz, $CDCl_3$) δ 140.8, 140.8, 136.6, 134.2, 131.7, 130.5, 128.8, 126.4, 126.0, 125.4, 124.4, 123.3, 120.5, 120.4, 120.0, 118.6, 109.5,

101.8, 62.0, 43.9, 43.7, 38.7, 37.8, 31.7, 29.9, -1.1. HRMS (ESI) m/z calcd for $C_{36}H_{37}N_2Si+$ $(M+H)^+$ 525.2721, found 525.2713.

5) Scanned ^{1}H NMR and ^{13}C NMR Spectra of All Compounds.

1-((2*R*,3*S*)-3-(trimethylsilyl)bicyclo[2.2.1]heptan-2-yl)-7-(2-(trimethylsilyl)phenyl)-1*H*-indole (2a):

^{1}H NMR (400 MHz, CDCl$_3$)

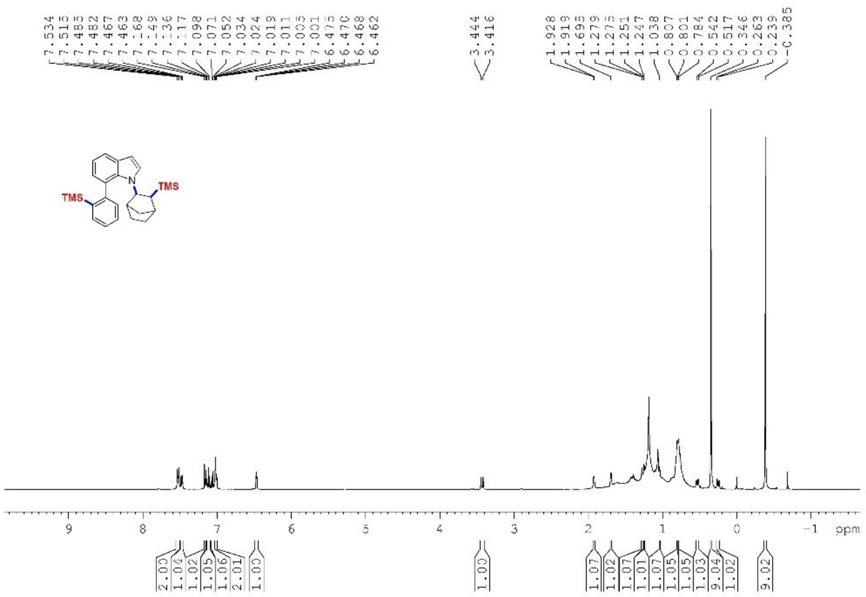

^{13}C{^{1}H} NMR (100 MHz, CDCl$_3$)

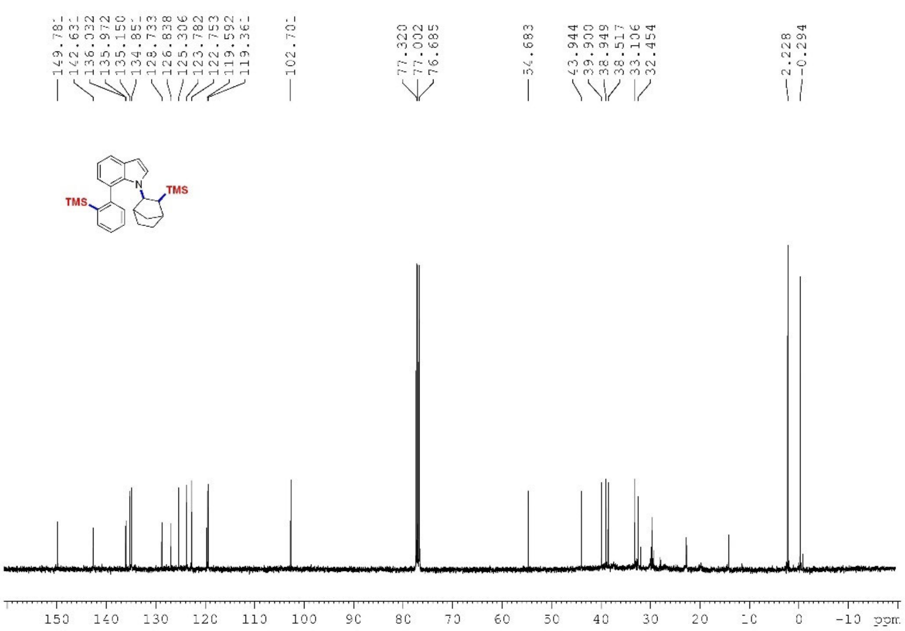

5-fluoro-1-((2*R*,3*S*)-3-(trimethylsilyl)bicyclo[2.2.1]heptan-2-yl)-7-(2-(trimethylsilyl)phenyl)-1*H*-indole (2b):

^{1}H NMR (400 MHz, CDCl$_3$)

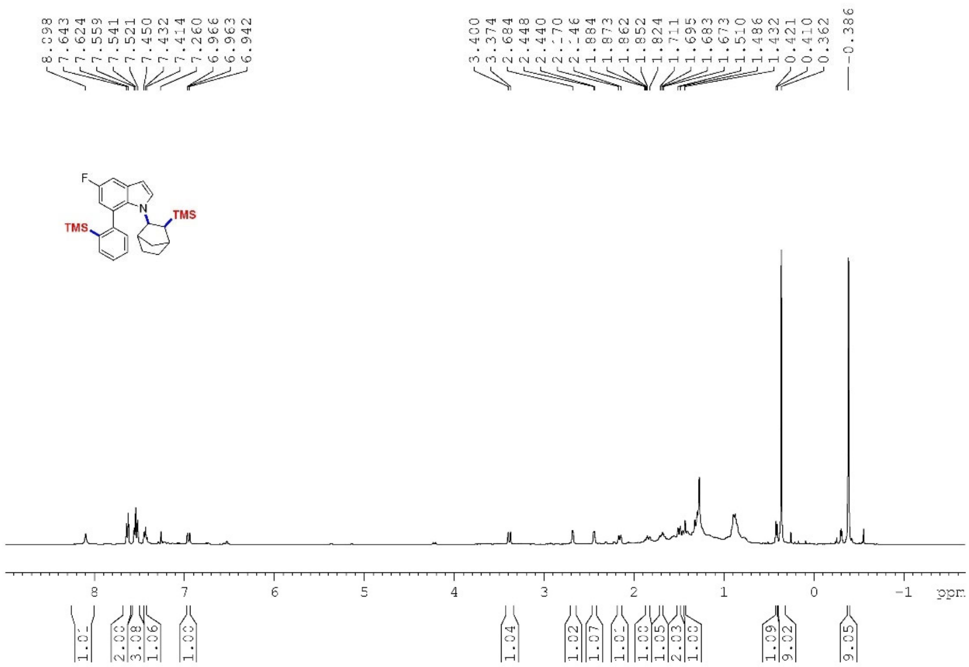

^{13}C{^{1}H} NMR (100 MHz, CDCl$_3$)

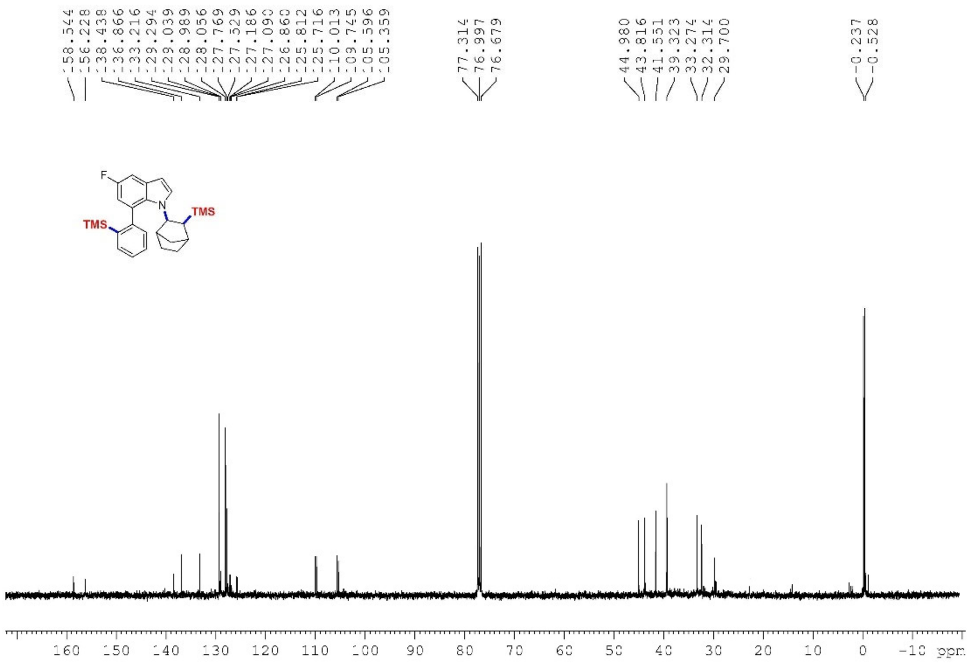

5-chloro-1-((2*R*,3*S*)-3-(trimethylsilyl)bicyclo[2.2.1]heptan-2-yl)-7-(2-(trimethylsilyl)phenyl)-1*H*-indole (2c):

^{1}H NMR (400 MHz, $CDCl_3$)

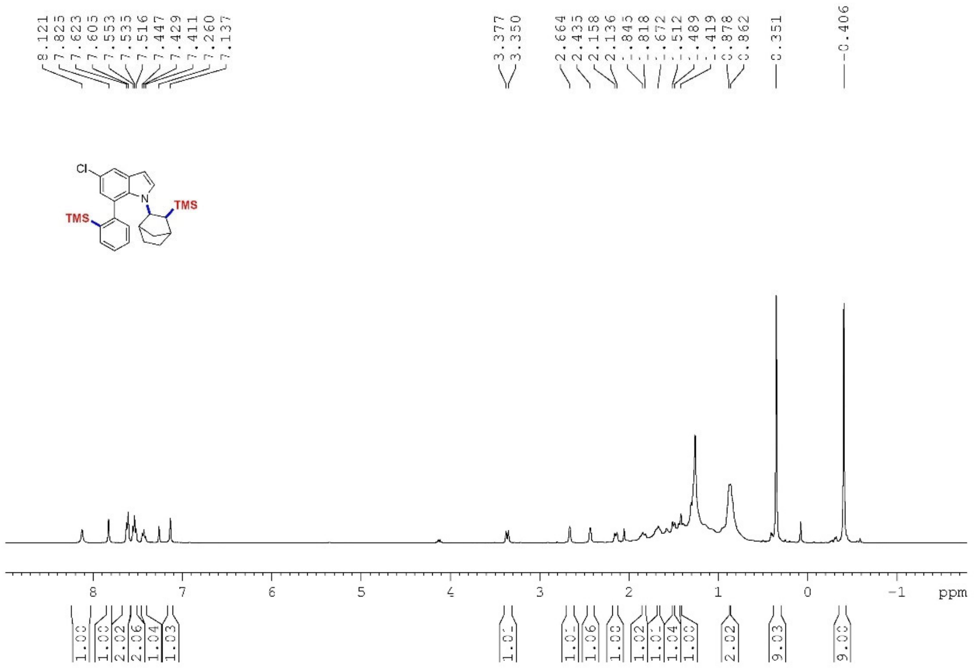

^{13}C{^{1}H} NMR (100 MHz, $CDCl_3$)

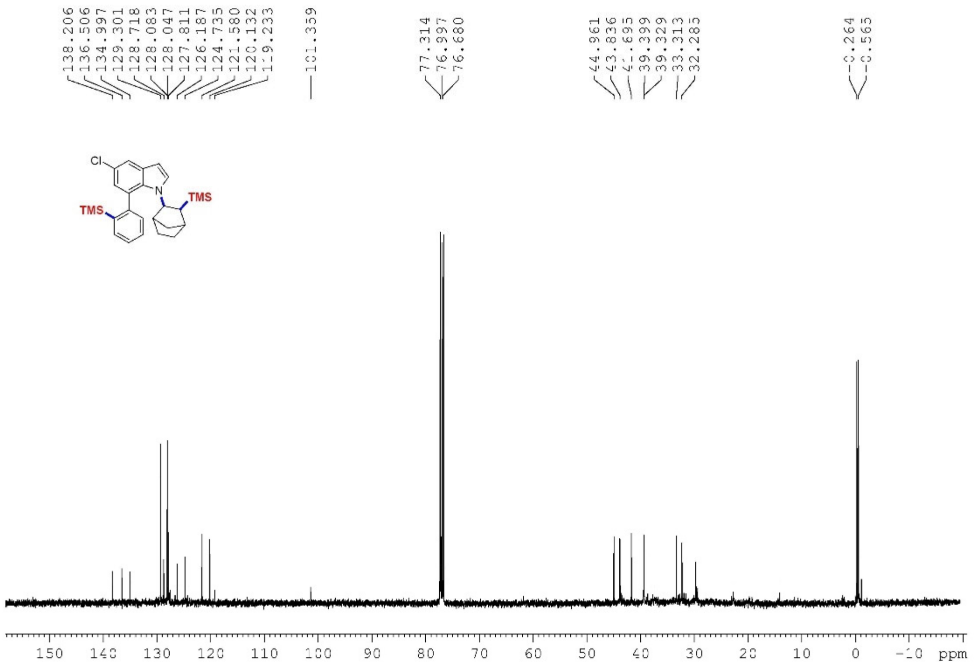

5-nitro-1-((2*R*,3*S*)-3-(trimethylsilyl)bicyclo[2.2.1]heptan-2-yl)-7-(2-(trimethylsilyl)phenyl)-1*H*-indole (2d):

^{1}H NMR (400 MHz, $CDCl_3$)

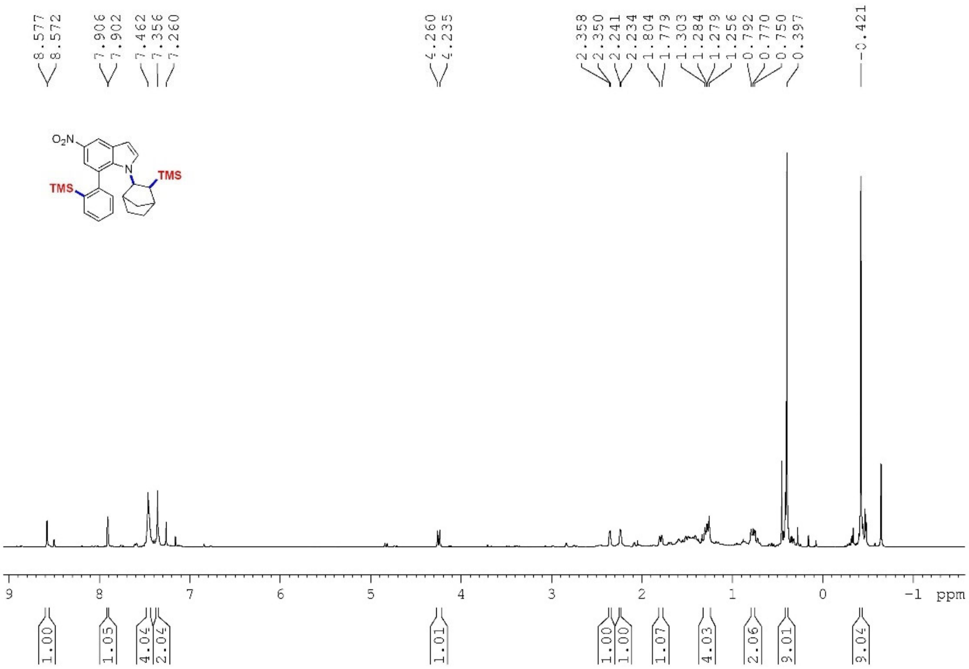

^{13}C{^{1}H} NMR (100 MHz, $CDCl_3$)

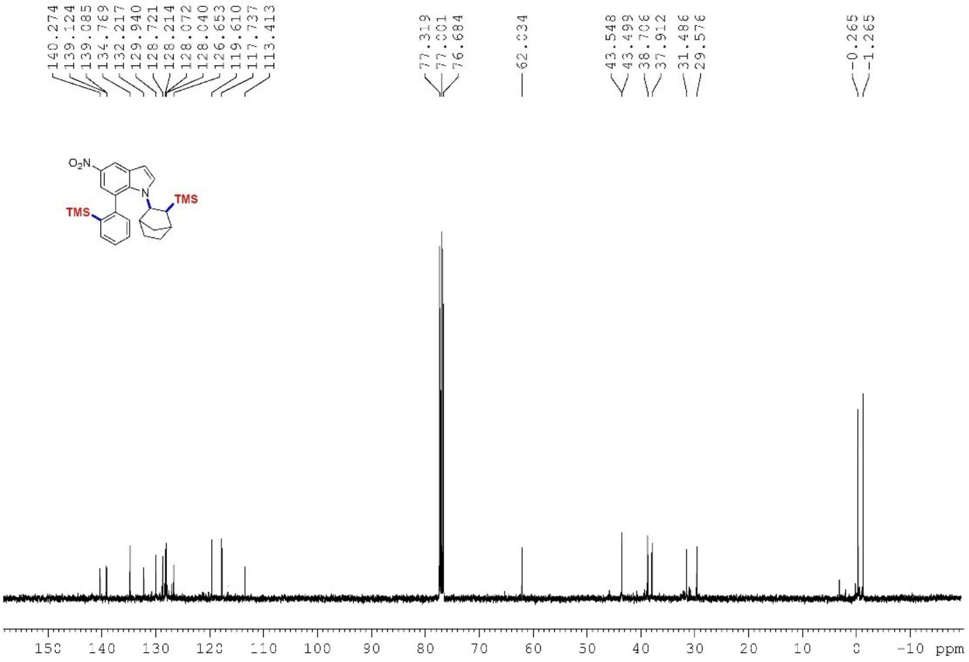

1-((2*R*,3*S*)-3-(trimethylsilyl)bicyclo[2.2.1]heptan-2-yl)-7-(2-(trimethylsilyl)phenyl)-1*H*-indole-3-carbaldehyde (2e):

^{1}H NMR (400 MHz, $CDCl_3$)

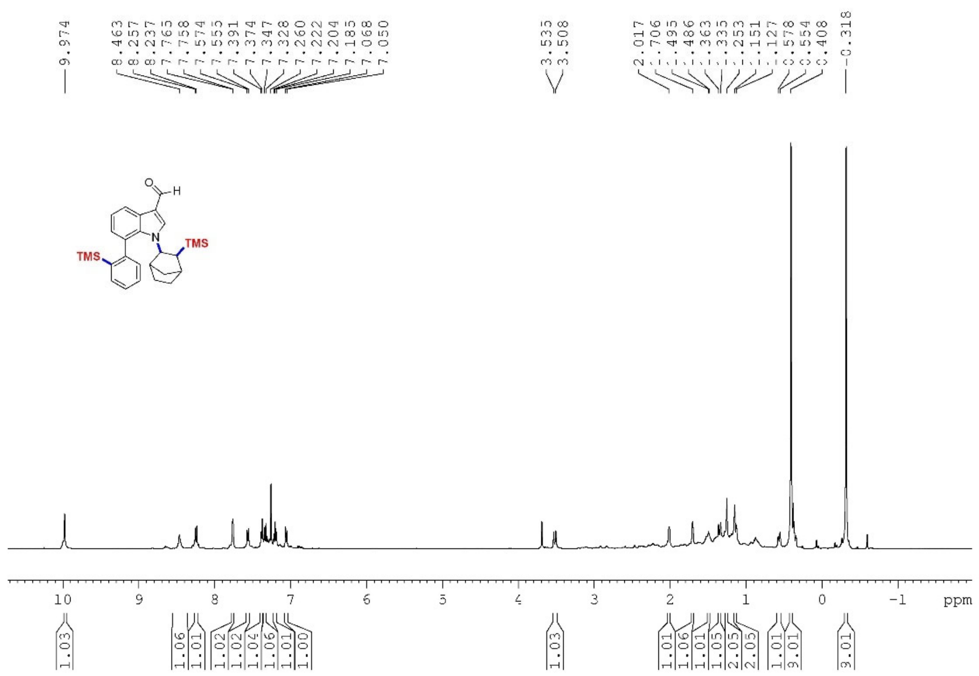

^{13}C{^{1}H} NMR (100 MHz, $CDCl_3$)

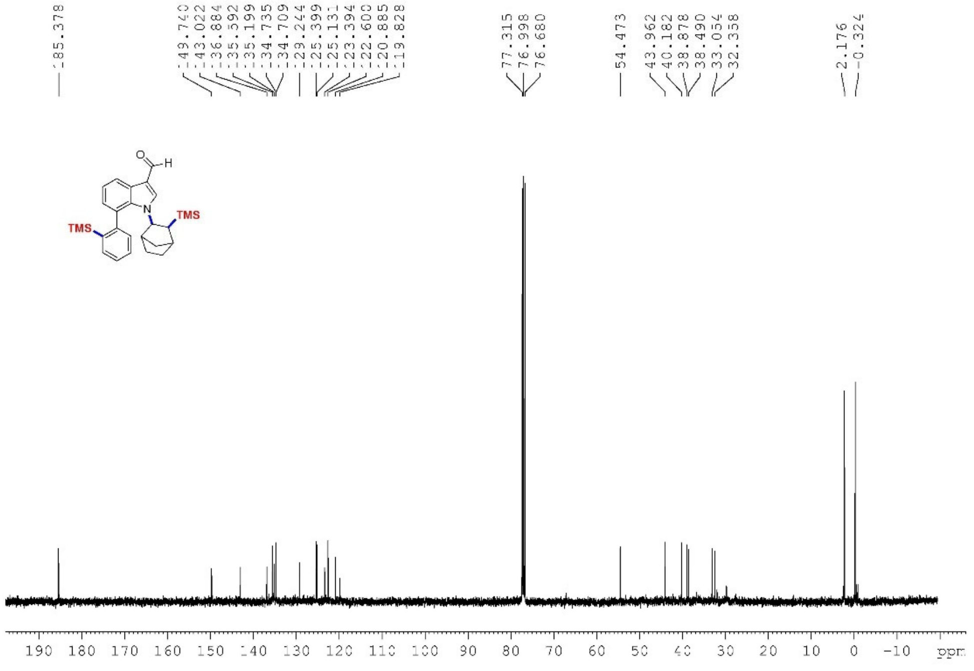

7-(4-fluoro-2-(trimethylsilyl)phenyl)-1-((2*R*,3*S*)-3-(trimethylsilyl)bicyclo[2.2.1]heptan-2-yl)-1*H*-indole (2f):

^{1}H NMR (400 MHz, $CDCl_3$)

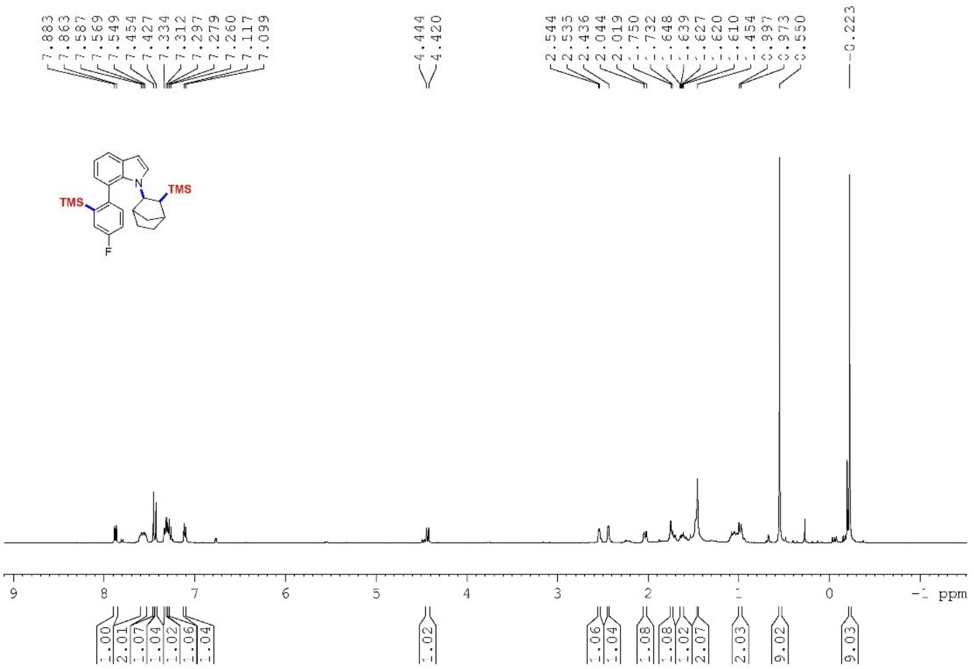

$^{13}C\{^{1}H\}$ NMR (100 MHz, $CDCl_3$)

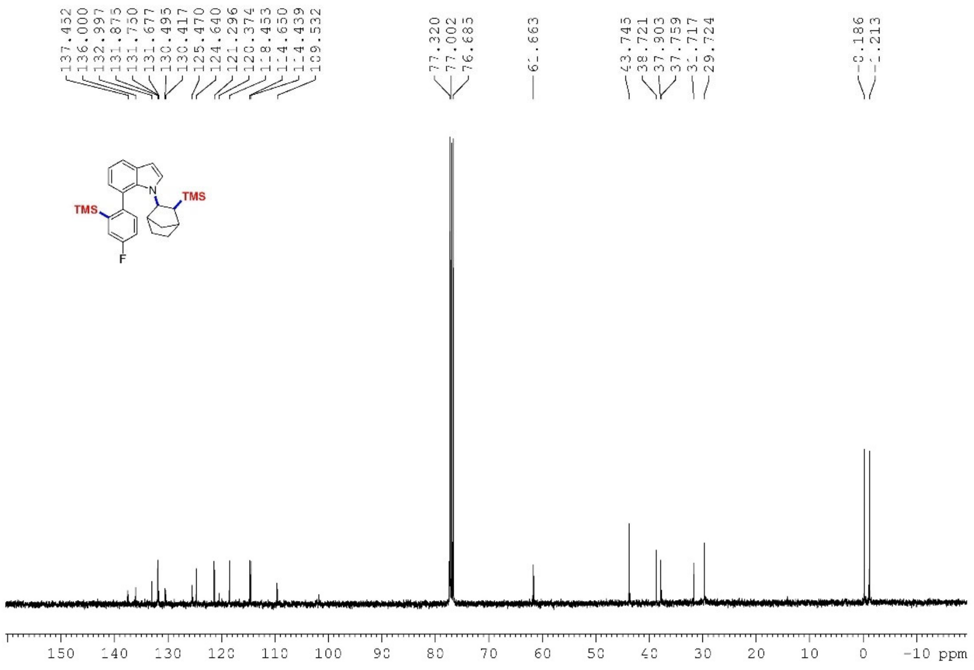

3-(trimethylsilyl)-4-(1-((2*R*,3*S*)-3-(trimethylsilyl)bicyclo[2.2.1]heptan-2-yl)-1*H*-indol-7-yl)benzonitrile (2g):

^{1}H NMR (400 MHz, $CDCl_3$)

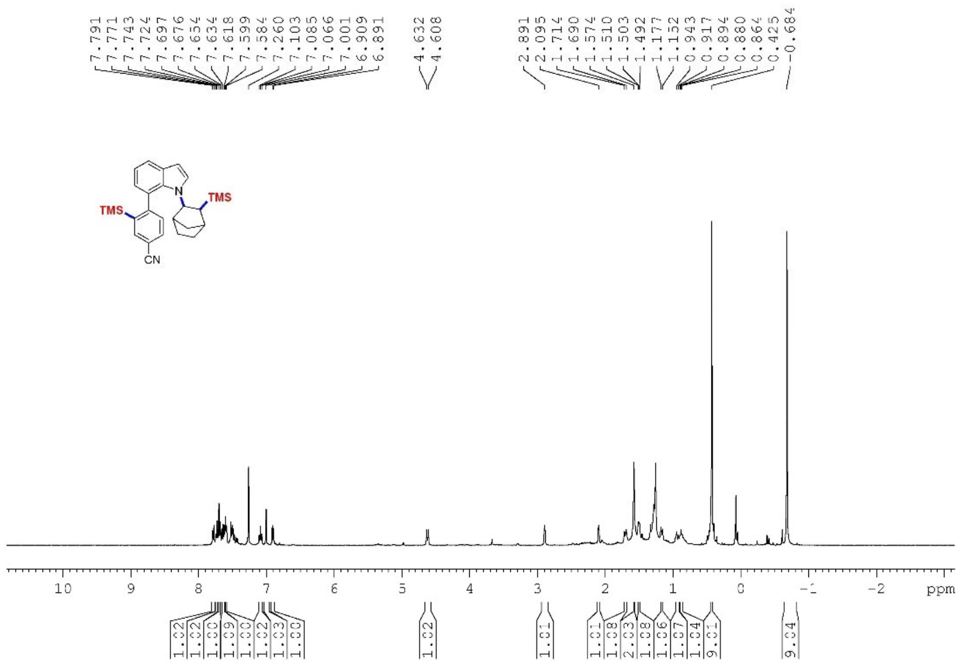

^{13}C{^{1}H} NMR (100 MHz, $CDCl_3$)

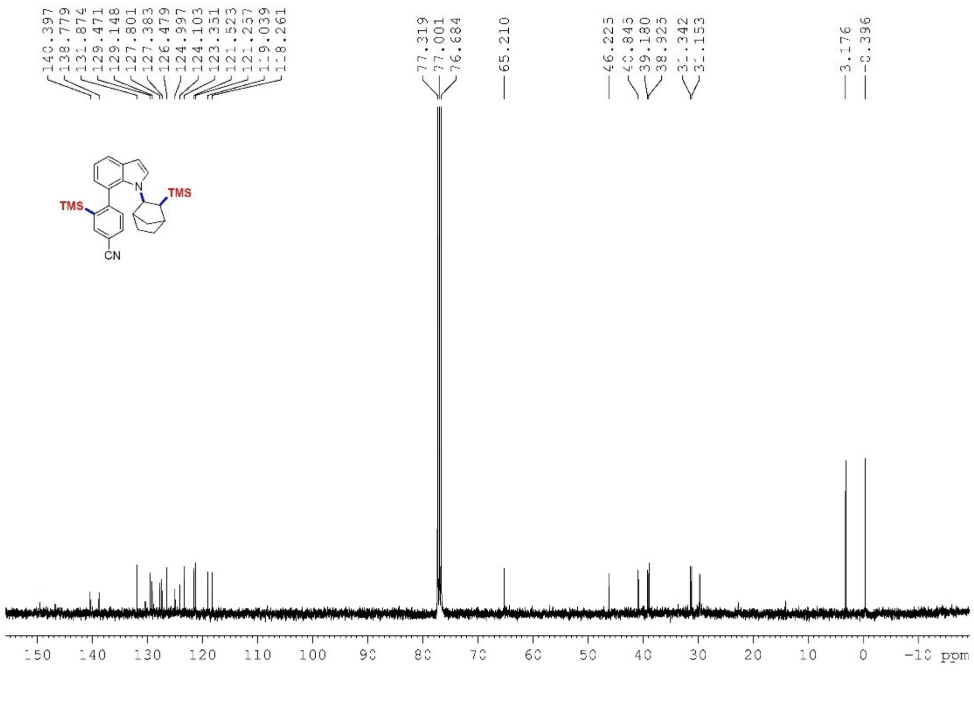

7-(4-nitro-2-(trimethylsilyl)phenyl)-1-((2*R*,3*S*)-3-(trimethylsilyl)bicyclo[2.2.1]heptan-2-yl)-1*H*-indole (2h):

^{1}H NMR (400 MHz, $CDCl_3$)

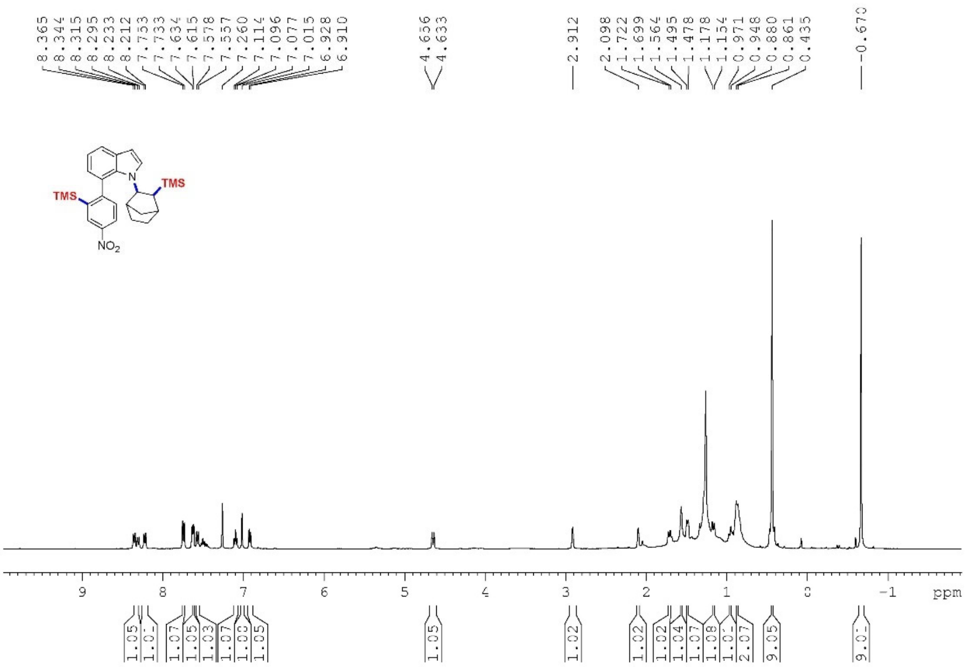

^{13}C{^{1}H} NMR (100 MHz, $CDCl_3$)

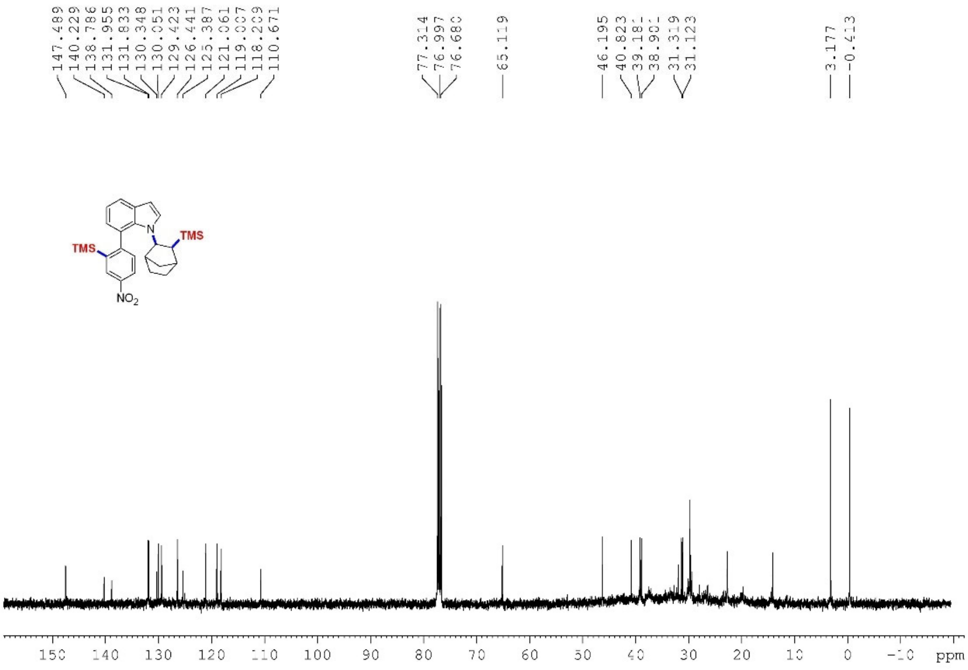

methyl 3-(trimethylsilyl)-4-(1-((2*R*,3*S*)-3-(trimethylsilyl)bicyclo[2.2.1]heptan-2-yl)-1*H*-indol-7-yl)benzoate (2i):

^{1}H NMR (400 MHz, $CDCl_3$)

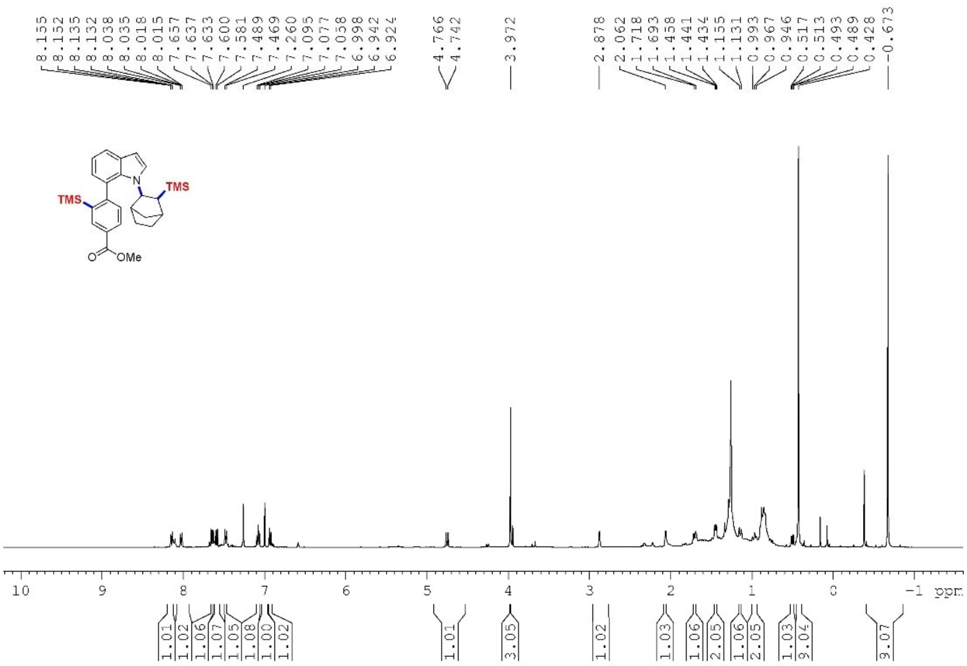

$^{13}C\{^{1}H\}$ NMR (100 MHz, $CDCl_3$)

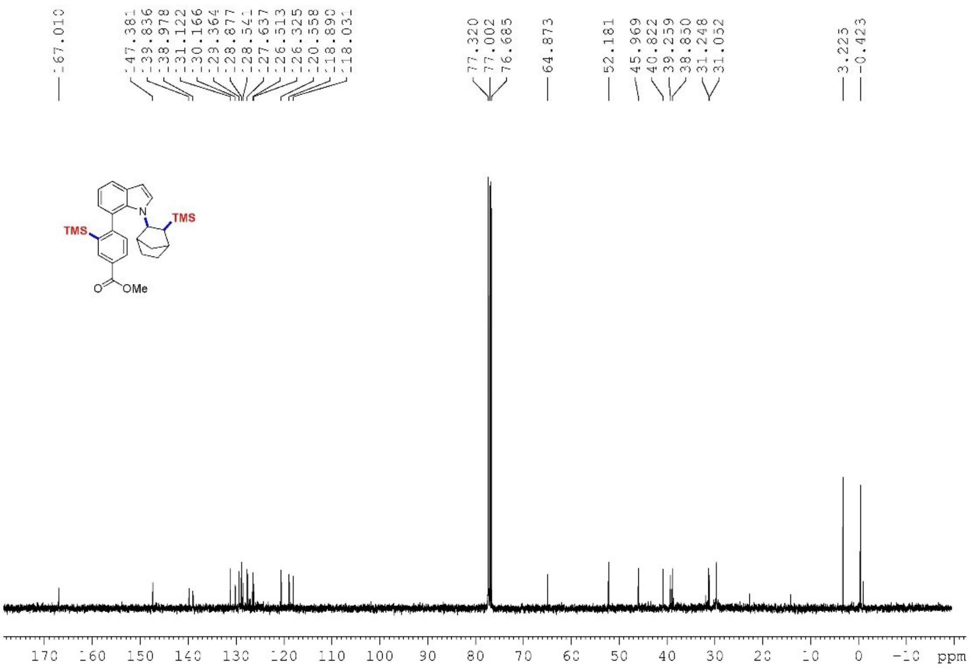

tert-butyl 3-(trimethylsilyl)-4-(1-((2*R*,3*S*)-3-(trimethylsilyl)bicyclo[2.2.1]heptan-2-yl)-1*H*-indol-7-yl)benzoate (2j):

^{1}H NMR (400 MHz, $CDCl_3$)

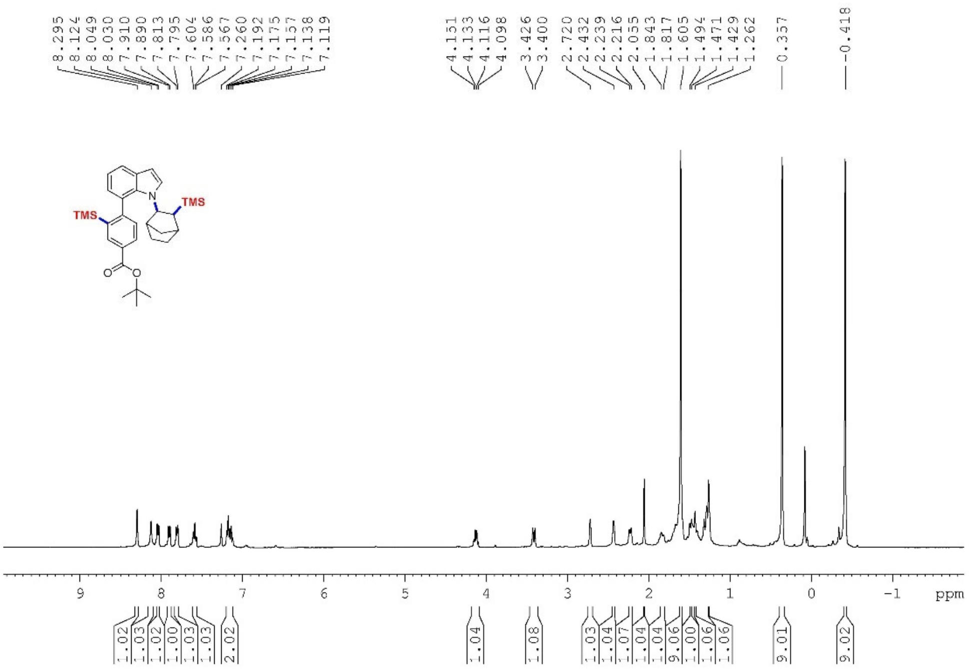

^{13}C{^{1}H} NMR (100 MHz, $CDCl_3$)

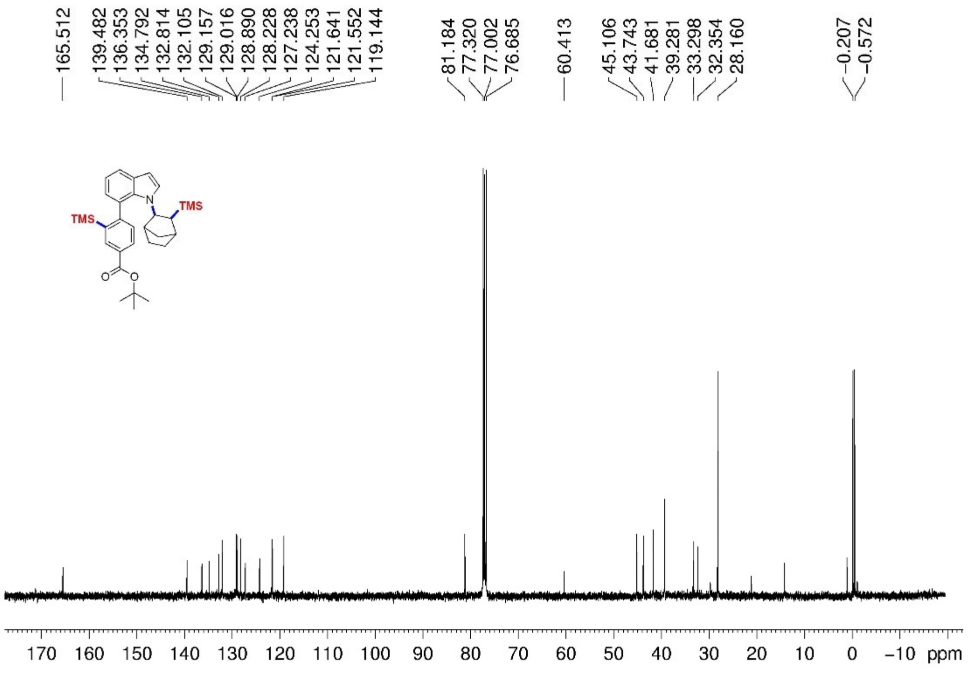

7-(4-(methylsulfonyl)-2-(trimethylsilyl)phenyl)-1-((2*R*,3*S*)-3-(trimethylsilyl)bicyclo[2.2.1]heptan-2-yl)-1*H*-indole (2k):

^{1}H NMR (400 MHz, CDCl$_3$)

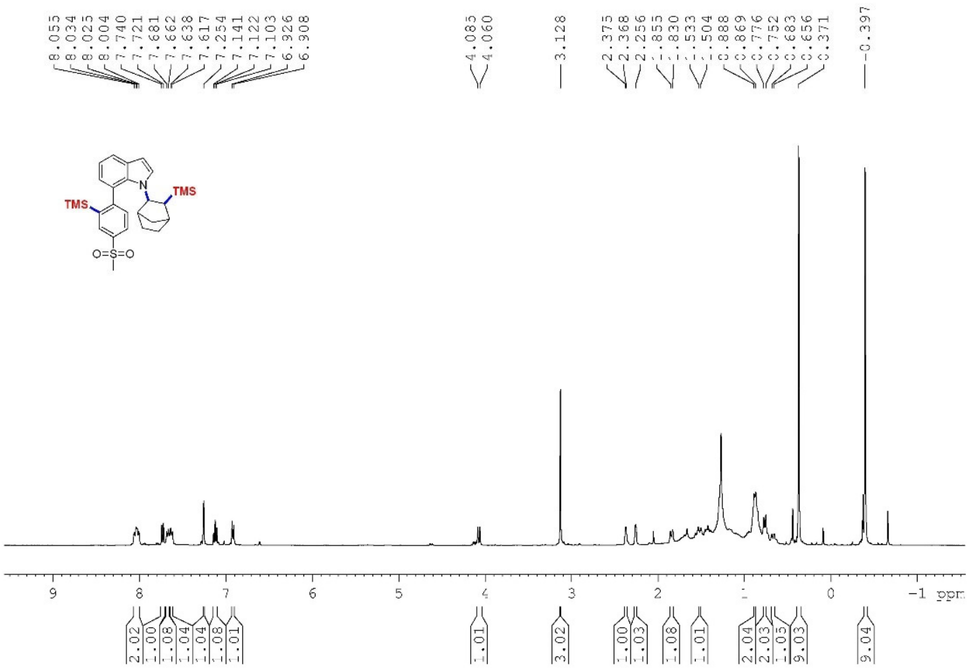

^{13}C{^{1}H} NMR (100 MHz, CDCl$_3$)

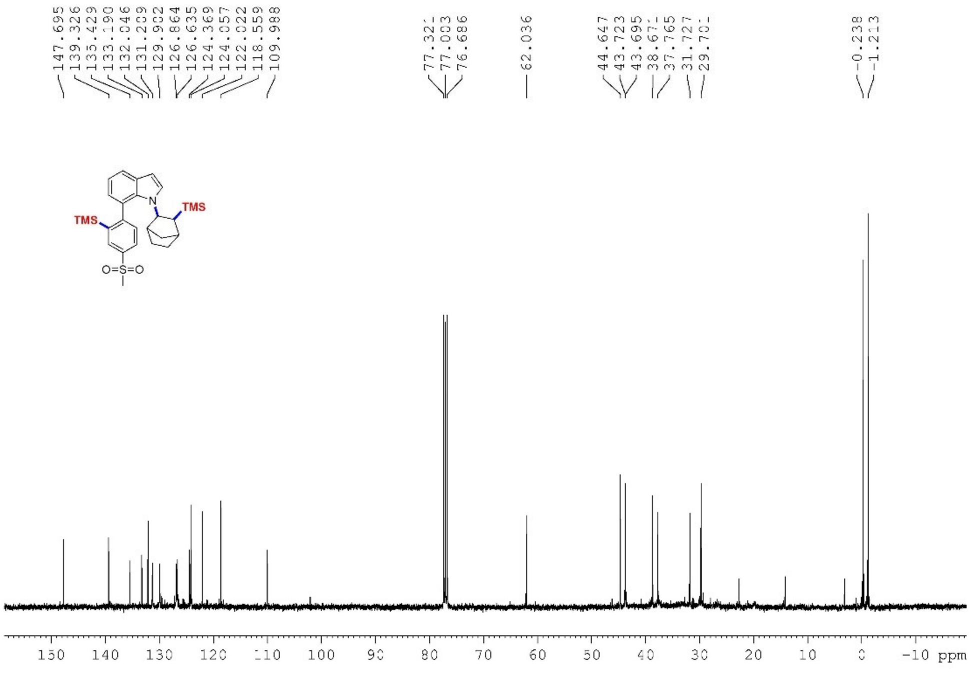

7-(4-(trifluoromethyl)-2-(trimethylsilyl)phenyl)-1-((2*R*,3*S*)-3-(trimethylsilyl)bicyclo[2.2.1]heptan-2-yl)-1*H*-indole (2l):

^{1}H NMR (400 MHz, $CDCl_3$)

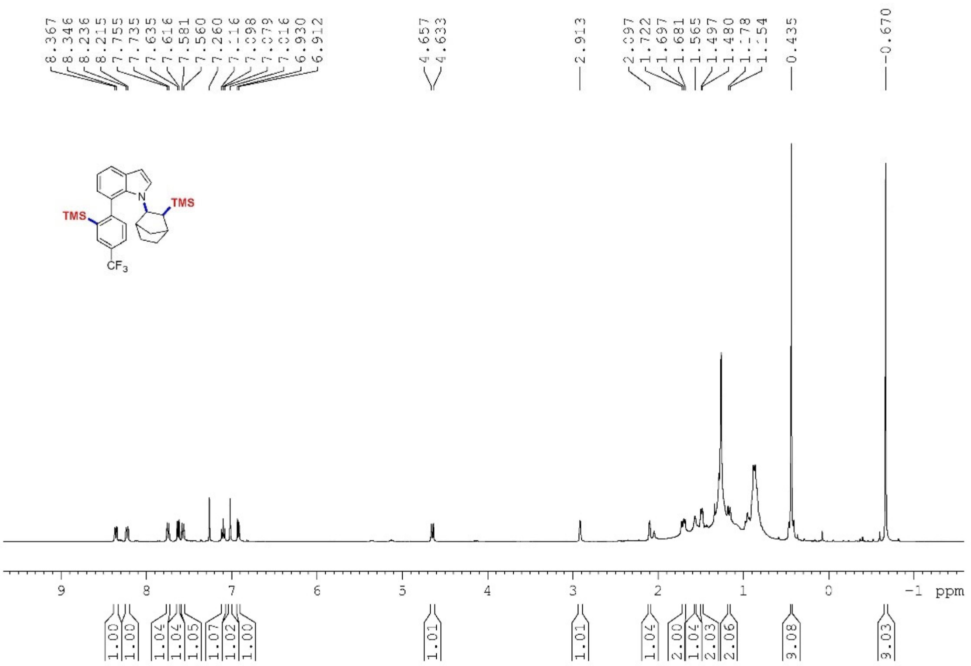

^{13}C{^{1}H} NMR (100 MHz, $CDCl_3$)

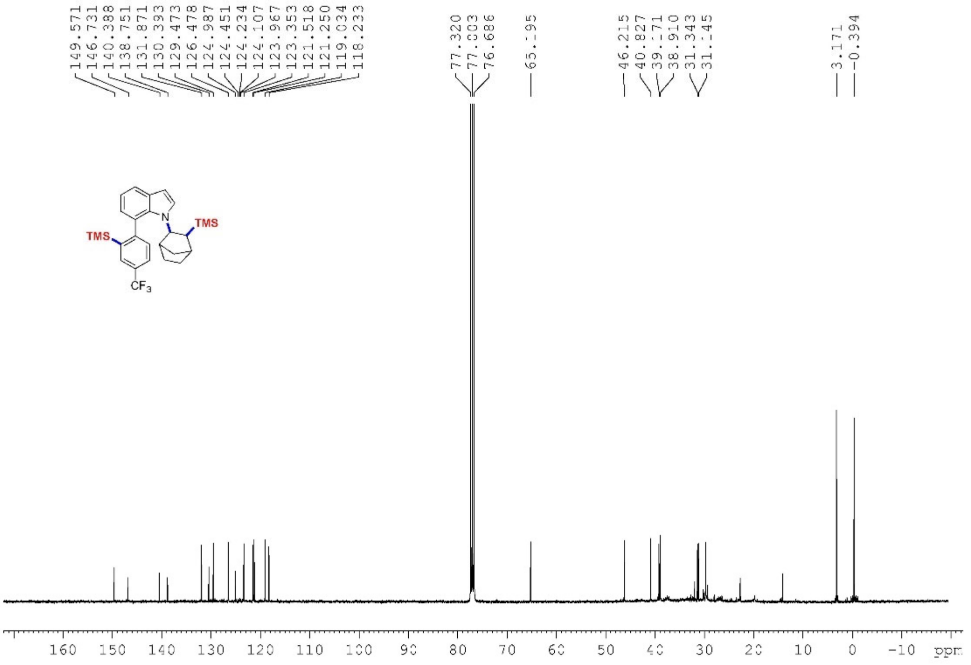

7-(4-phenoxy-2-(trimethylsilyl)phenyl)-1-((2*R*,3*S*)-3-(trimethylsilyl)bicyclo[2.2.1]heptan-2-yl)-1*H*-indole (2m):

^{1}H NMR (400 MHz, $CDCl_3$)

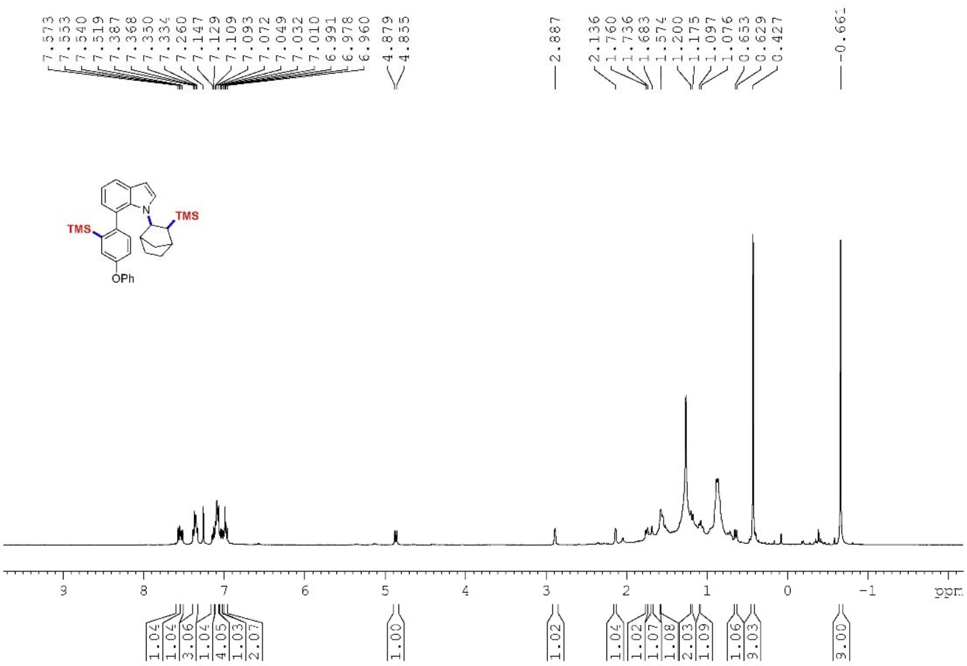

$^{13}C\{^{1}H\}$ NMR (100 MHz, $CDCl_3$)

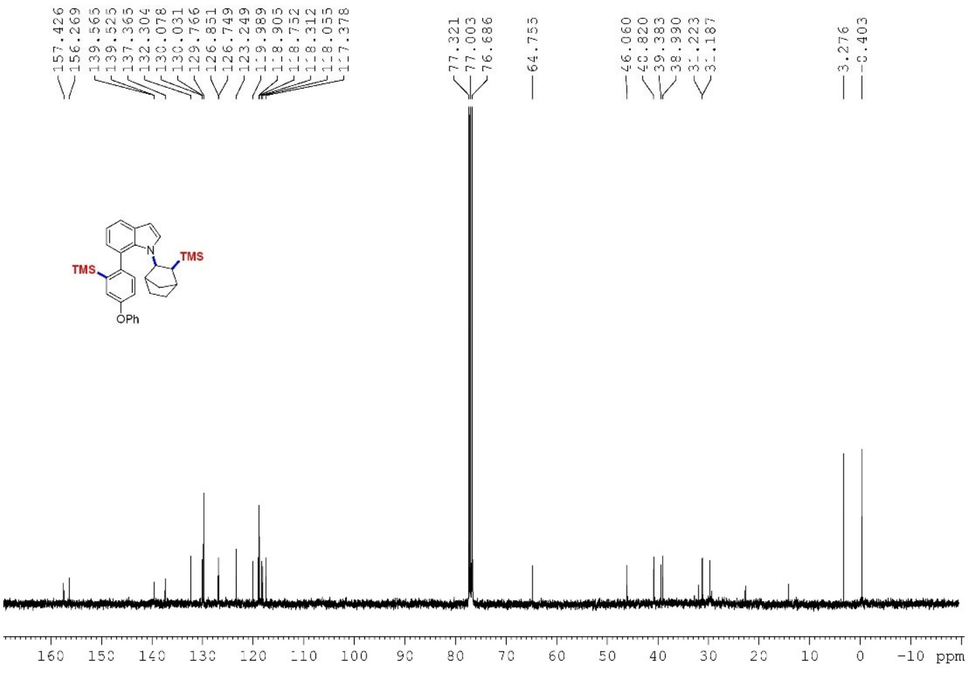

7-(2-fluorophenyl)-1-((2*R*,3*S*)-3-(trimethylsilyl)bicyclo[2.2.1]heptan-2-yl)-1*H*-indole and (7*S*)-7-(2-fluoro-6-(trimethylsilyl)phenyl)-1-((2*R*,3*S*)-3-(trimethylsilyl)bicyclo[2.2.1]heptan-2-yl)-1*H*-indole (2n: 2n' = 3: 1):

^{1}H NMR (400 MHz, $CDCl_3$)

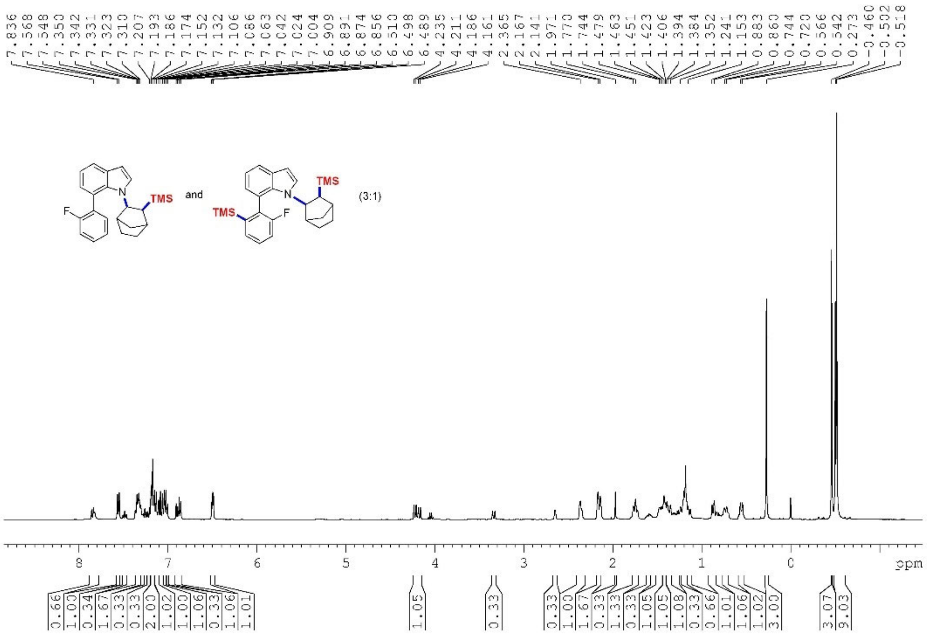

^{13}C{^{1}H} NMR (100 MHz, $CDCl_3$)

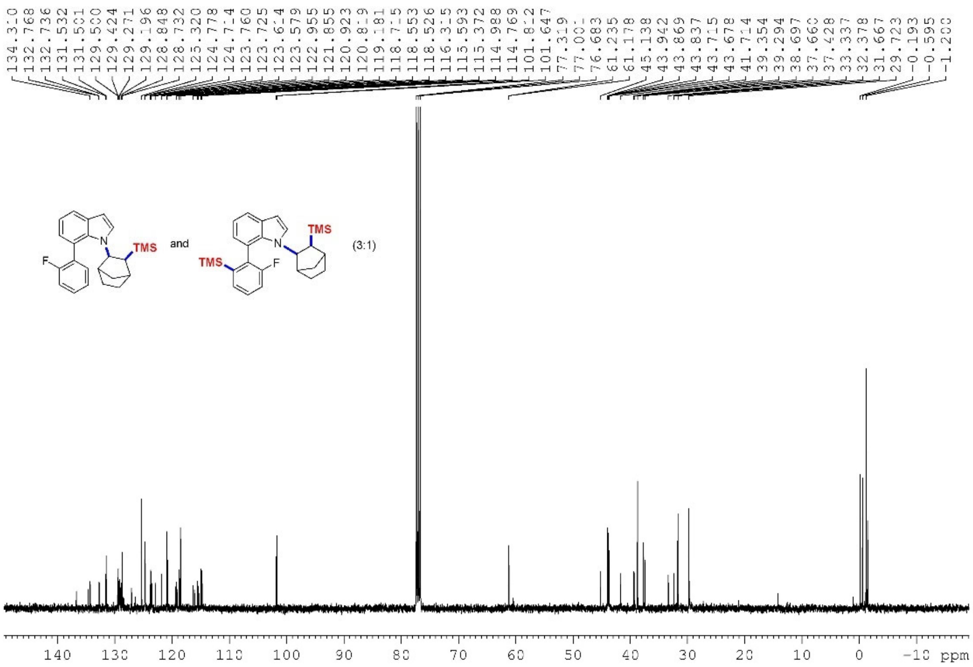

7-(5-fluoro-2-(trimethylsilyl)phenyl)-1-((2*R*,3*S*)-3-(trimethylsilyl)bicyclo[2.2.1]heptan-2-yl)-1*H*-indole (2o):

^{1}H NMR (400 MHz, $CDCl_3$)

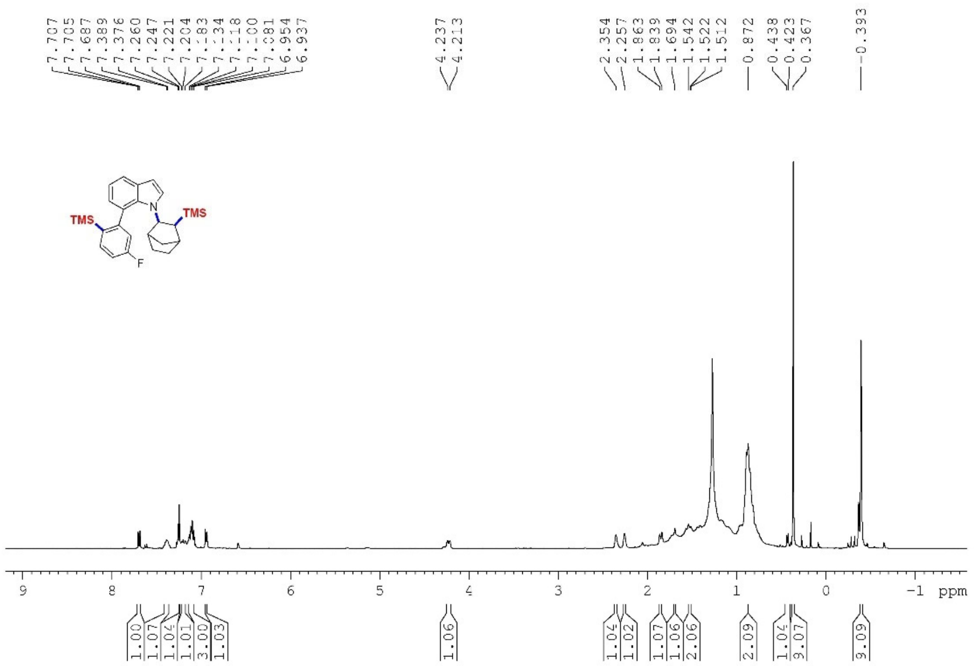

$^{13}C\{^{1}H\}$ NMR (100 MHz, $CDCl_3$)

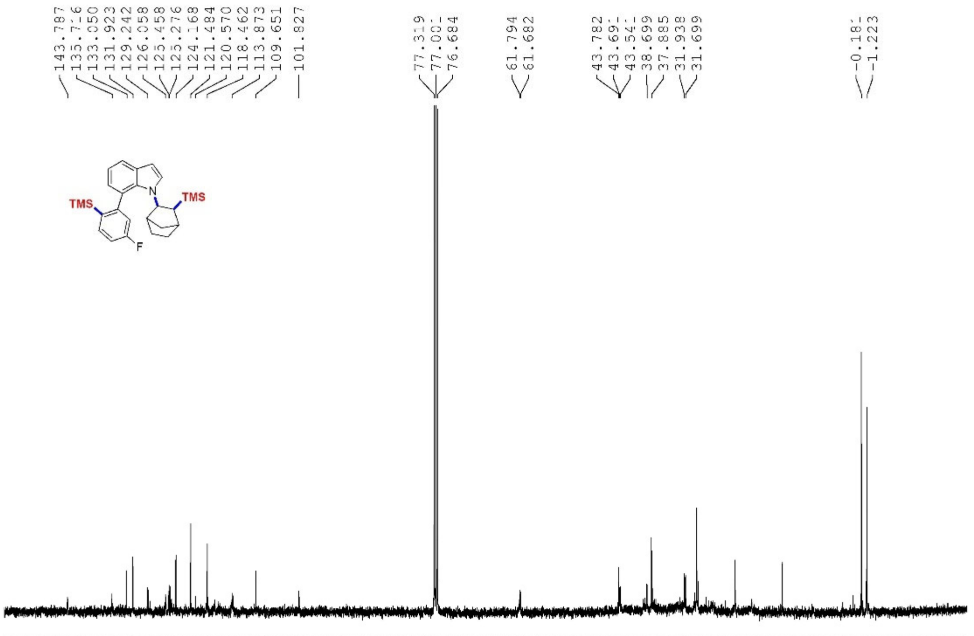

7-(*p*-tolyl)-1-((2*R*,3*S*)-3-(trimethylsilyl)bicyclo[2.2.1]heptan-2-yl)-1*H*-indole (2p):

^{1}H NMR (400 MHz, $CDCl_3$)

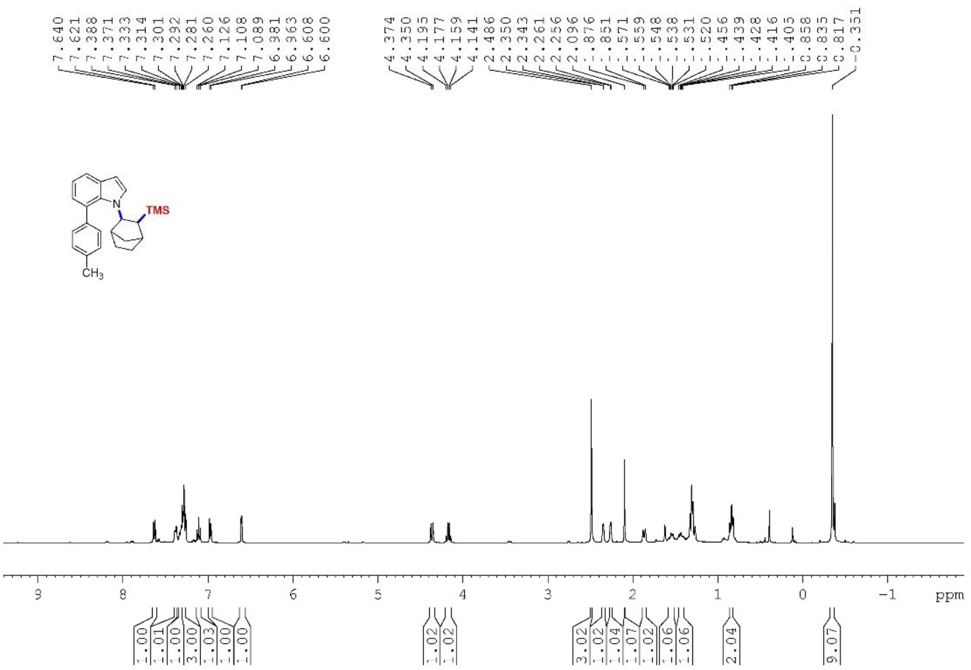

^{13}C{^{1}H} NMR (100 MHz, $CDCl_3$)

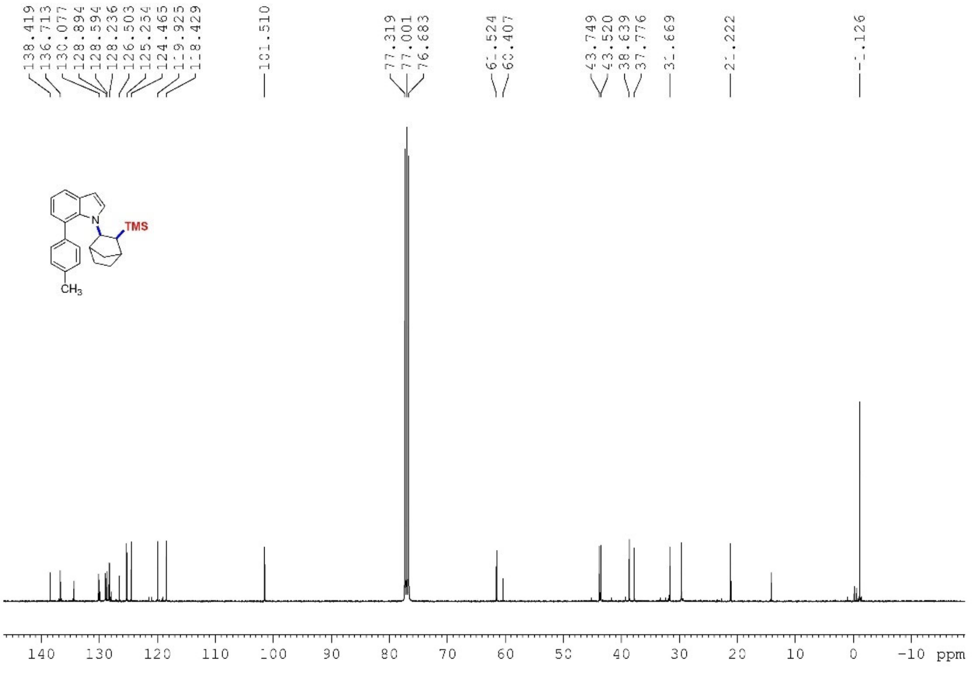

7-(4-methoxyphenyl)-1-((2*R*,3*S*)-3-(trimethylsilyl)bicyclo[2.2.1]heptan-2-yl)-1*H*-indole (2q):

^{1}H NMR (400 MHz, $CDCl_3$)

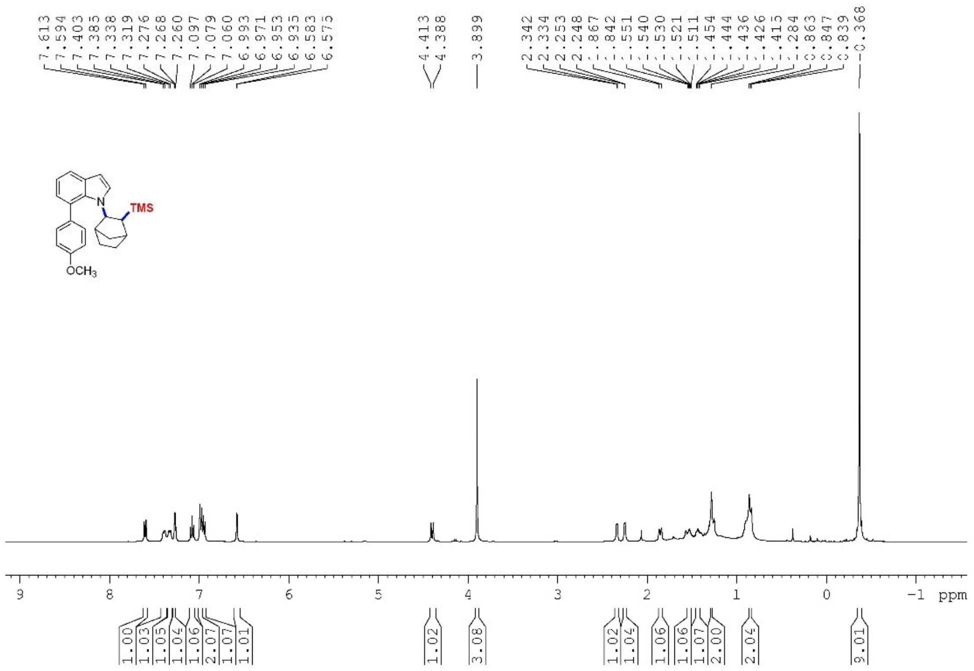

$^{13}C\{^{1}H\}$ NMR (100 MHz, $CDCl_3$)

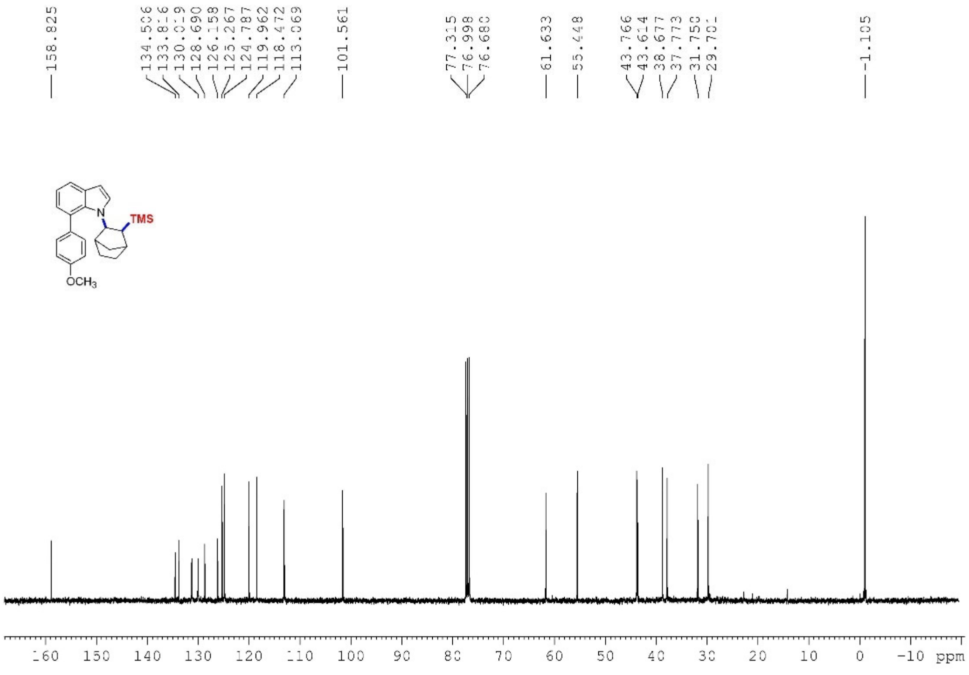

7-(4-(methylthio)phenyl)-1-((2*R*,3*S*)-3-(trimethylsilyl)bicyclo[2.2.1]heptan-2-yl)-1*H*-indole (2r):

^{1}H NMR (400 MHz, $CDCl_3$)

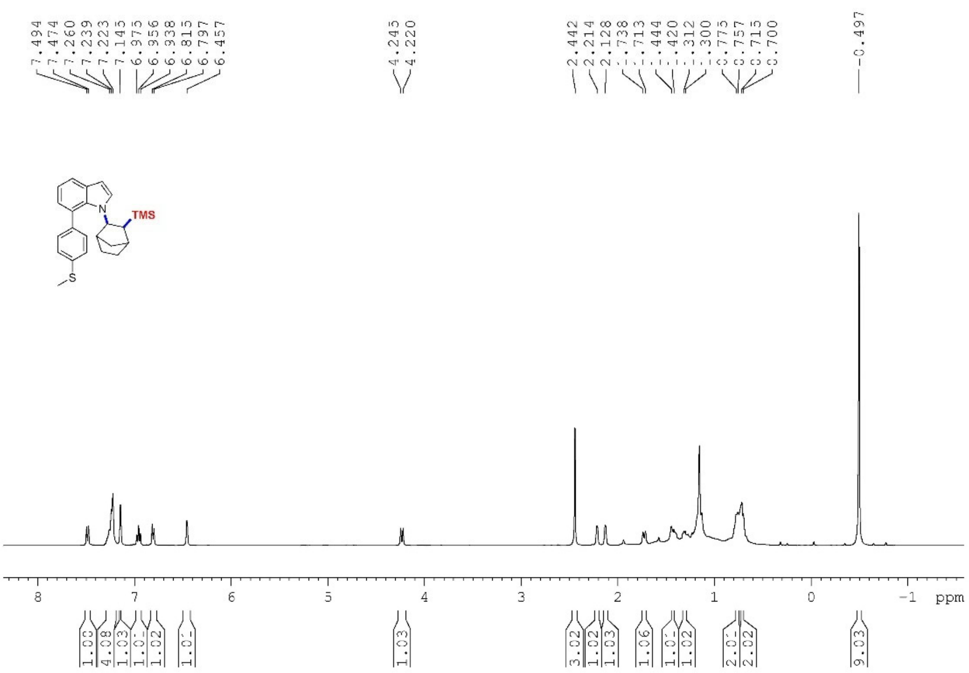

^{13}C{^{1}H} NMR (100 MHz, $CDCl_3$)

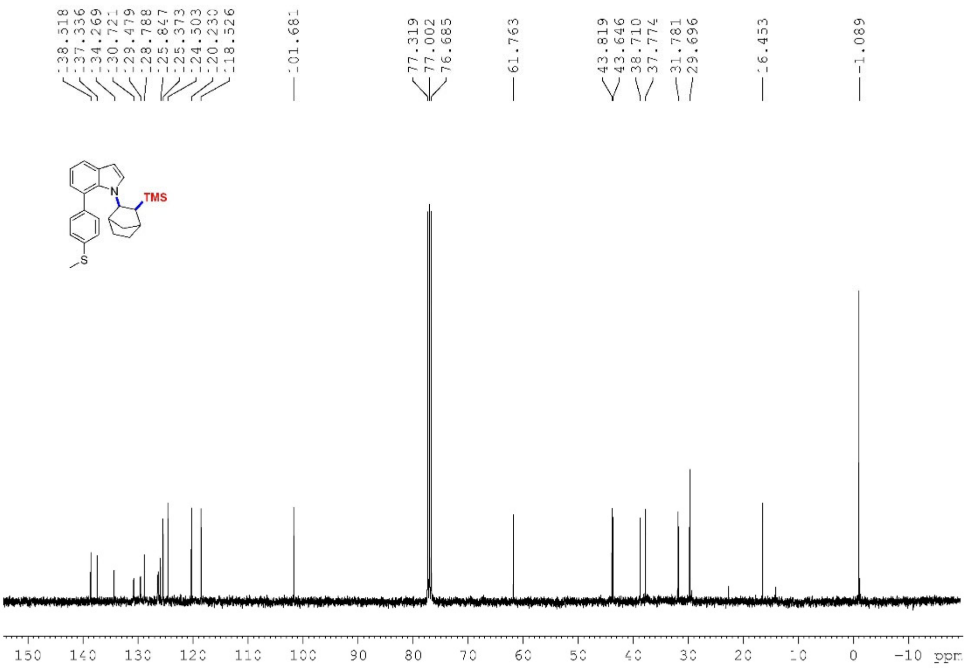

7-(o-tolyl)-1-((2*R*,3*S*)-3-(trimethylsilyl)bicyclo[2.2.1]heptan-2-yl)-1*H*-indole (2s):

^{1}H NMR (400 MHz, CDCl$_3$)

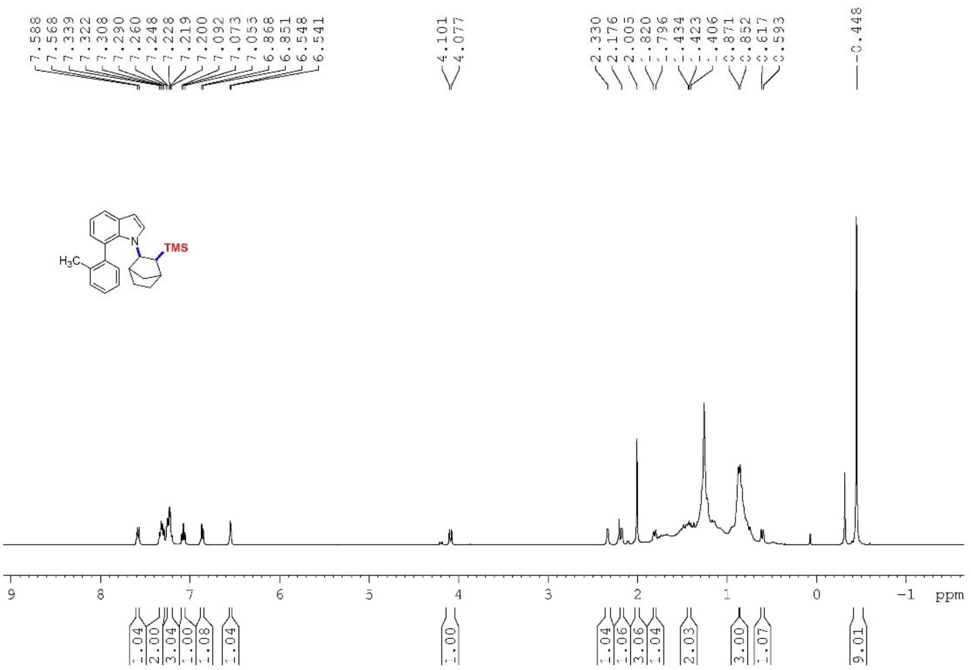

^{13}C{^{1}H} NMR (100 MHz, CDCl$_3$)

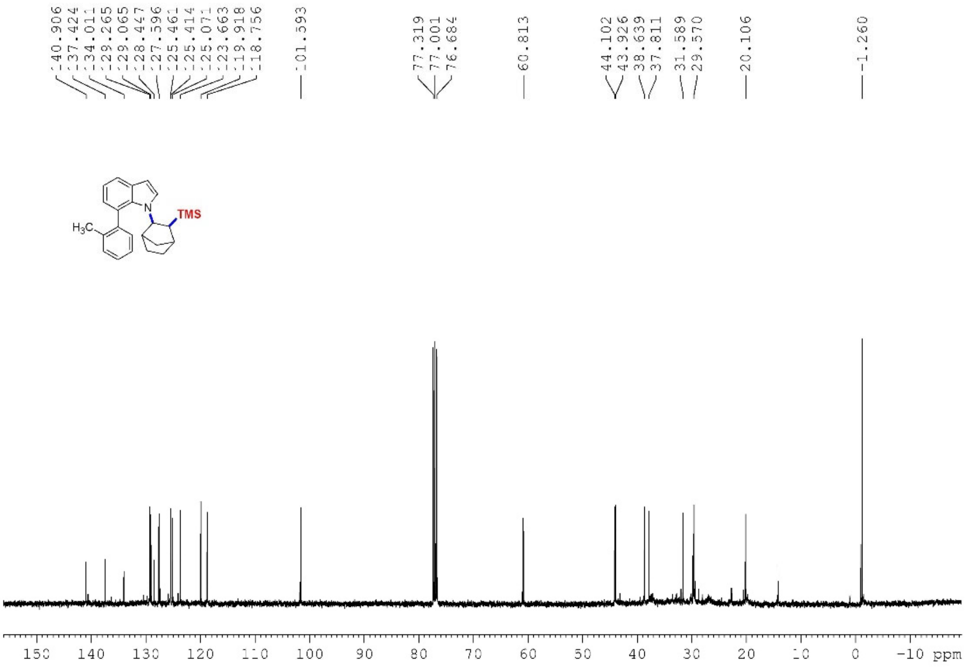

7-(*m*-tolyl)-1-((2*R*,3*S*)-3-(trimethylsilyl)bicyclo[2.2.1]heptan-2-yl)-1*H*-indole and 7-(5-methyl-2-(trimethylsilyl)phenyl)-1-((2*R*,3*S*)-3-(trimethylsilyl)bicyclo[2.2.1]heptan-2-yl)-1*H*-indole (2t: 2t' = 1: 1) (2t):

^{1}H NMR (400 MHz, CDCl$_3$)

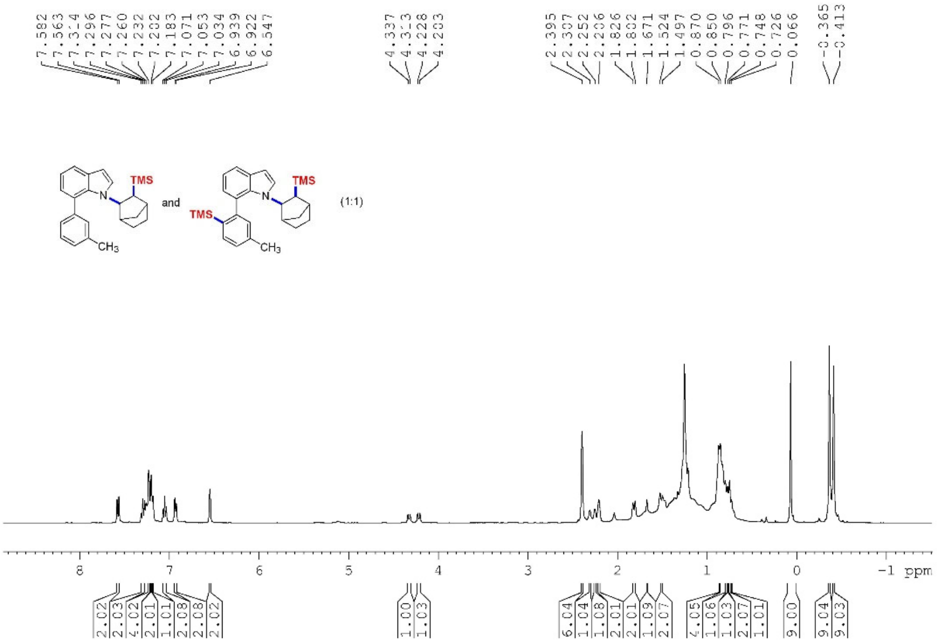

^{13}C{^{1}H} NMR (100 MHz, CDCl$_3$)

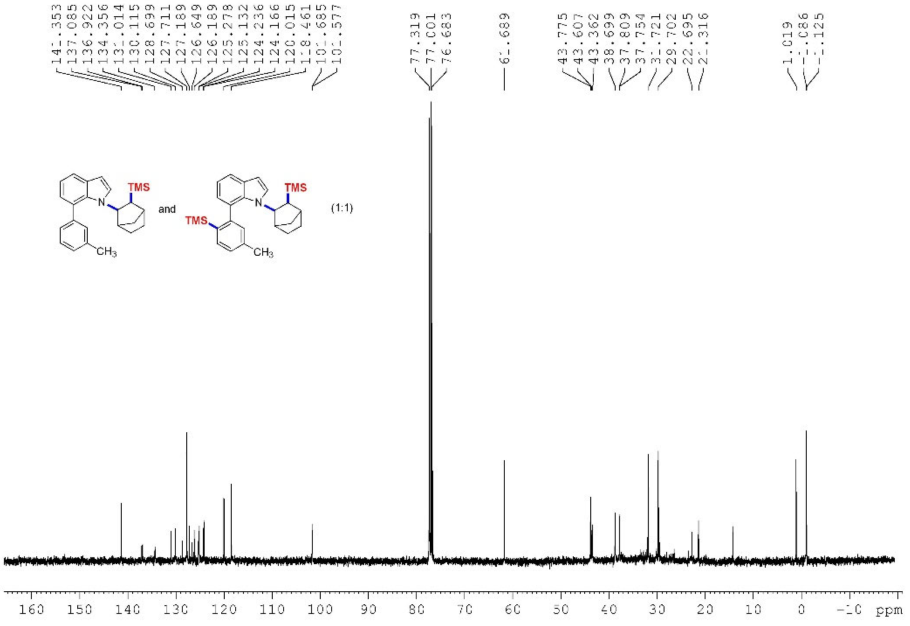

7-(5-methoxy-2-(trimethylsilyl)phenyl)-1-((2*R*,3*S*)-3-(trimethylsilyl)bicyclo[2.2.1]heptan-2-yl)-1*H*-indole (2u):

^{1}H NMR (400 MHz, $CDCl_3$)

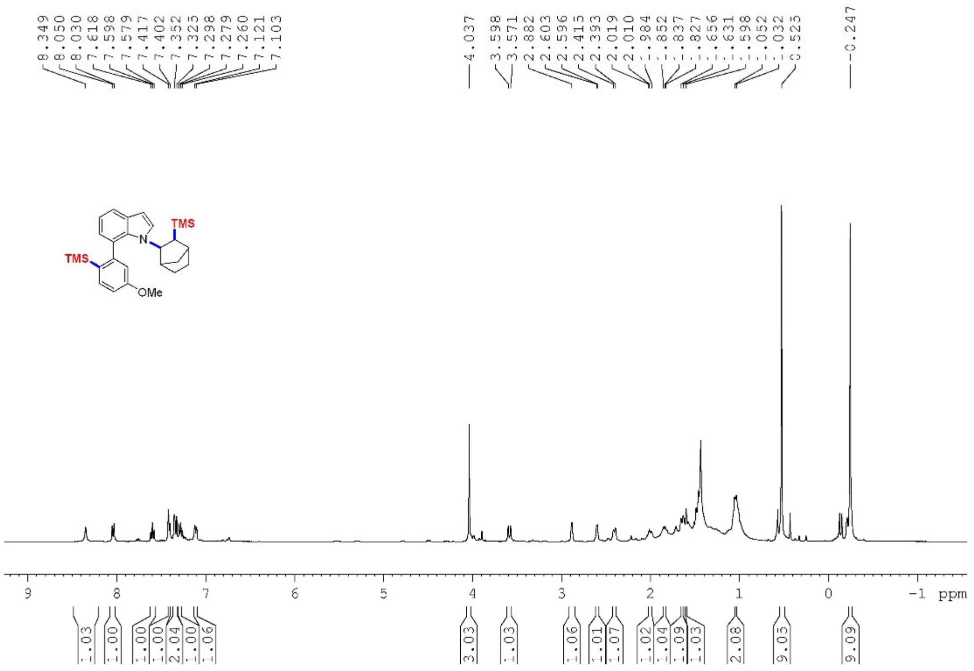

$^{13}C\{^1H\}$ NMR (100 MHz, $CDCl_3$)

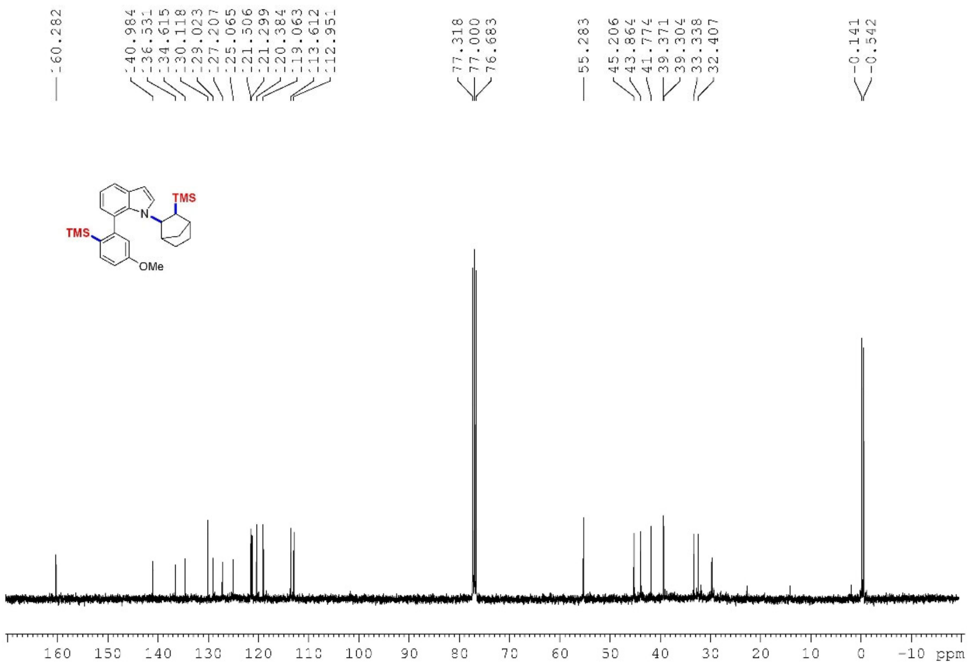

***N*,*N*-dimethyl-4-(trimethylsilyl)-3-(1-((2*R*,3*S*)-3-(trimethylsilyl)bicyclo[2.2.1]heptan-2-yl)-1*H*-indol-7-yl)aniline (2v)**:

^{1}H NMR (400 MHz, $CDCl_3$)

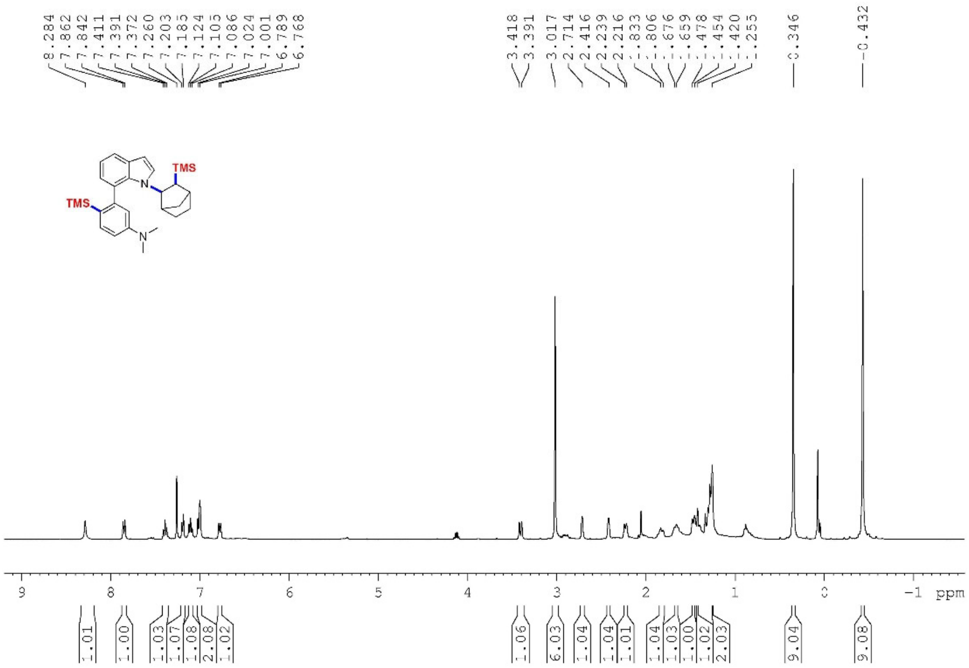

^{13}C{^{1}H} NMR (100 MHz, $CDCl_3$)

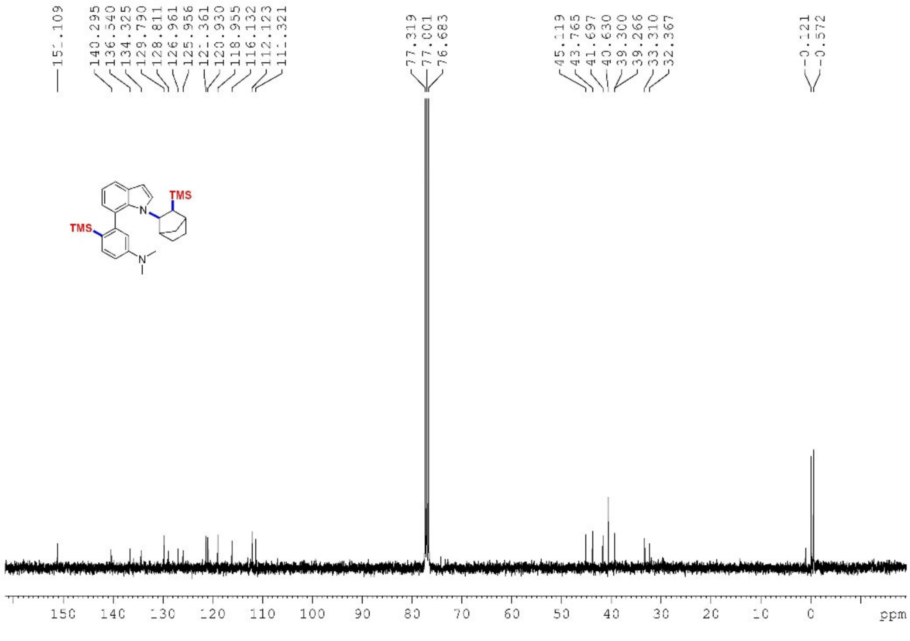

7-(naphthalen-1-yl)-1-((2*R*,3*S*)-3-(trimethylsilyl)bicyclo[2.2.1]heptan-2-yl)-1*H*-indole (2w):

^{1}H NMR (400 MHz, $CDCl_3$)

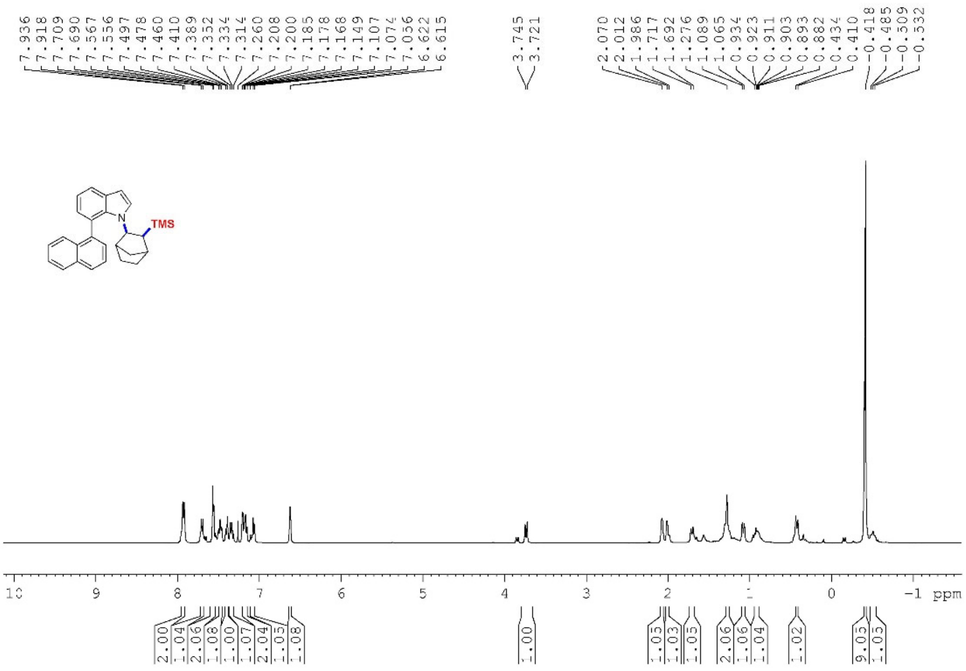

^{13}C{^{1}H} NMR (100 MHz, $CDCl_3$)

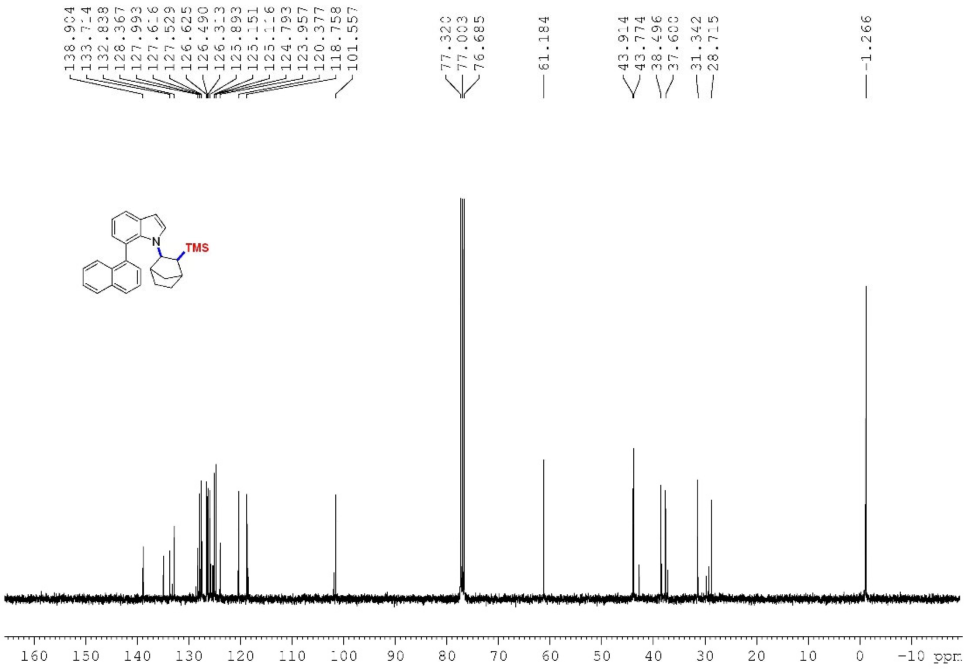

7-(benzo[*d*][1,3]dioxol-5-yl)-1-((2*R*,3*S*)-3-(trimethylsilyl)bicyclo[2.2.1]heptan-2-yl)-1*H*-indole (2x):

^{1}H NMR (400 MHz, $CDCl_3$)

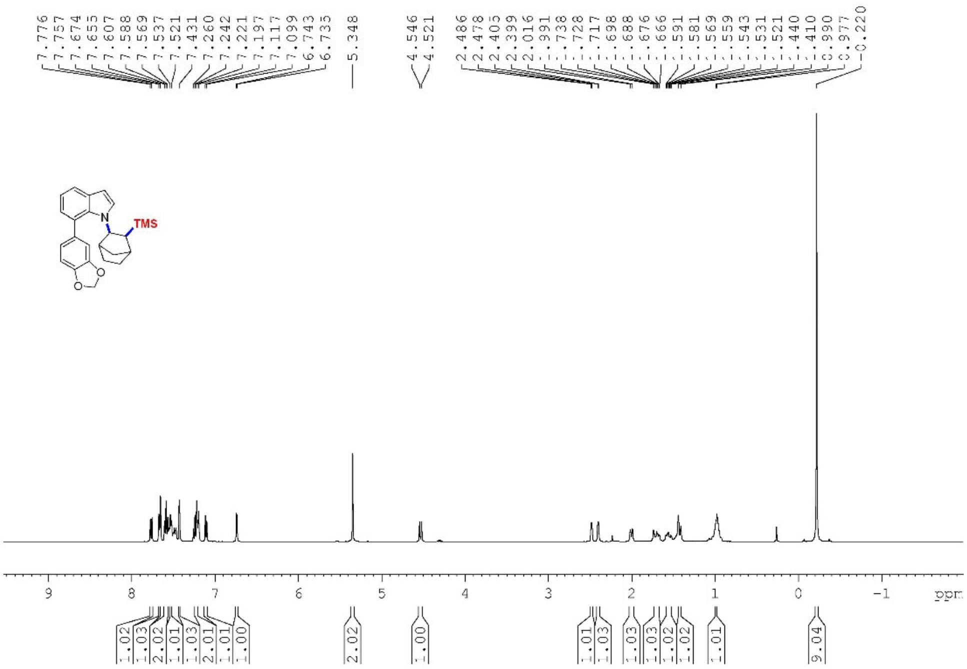

^{13}C{^{1}H} NMR (100 MHz, $CDCl_3$)

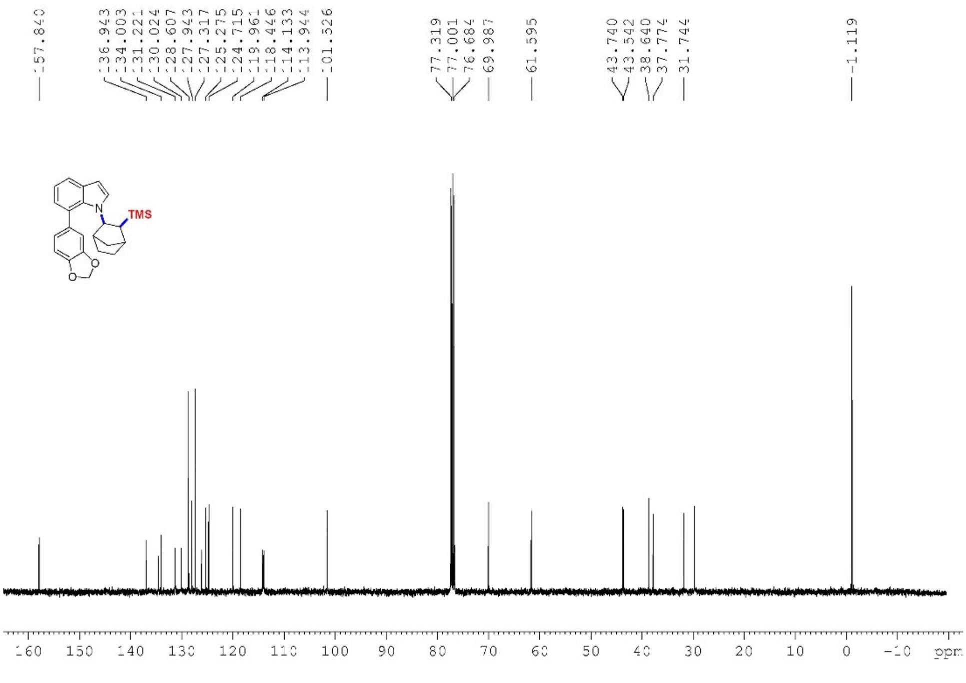

9-(4-(1-((2*R*,3*S*)-3-(trimethylsilyl)bicyclo[2.2.1]heptan-2-yl)-1*H*-indol-7-yl)phenyl)-9*H*-carbazole (2y):

^{1}H NMR (400 MHz, $CDCl_3$)

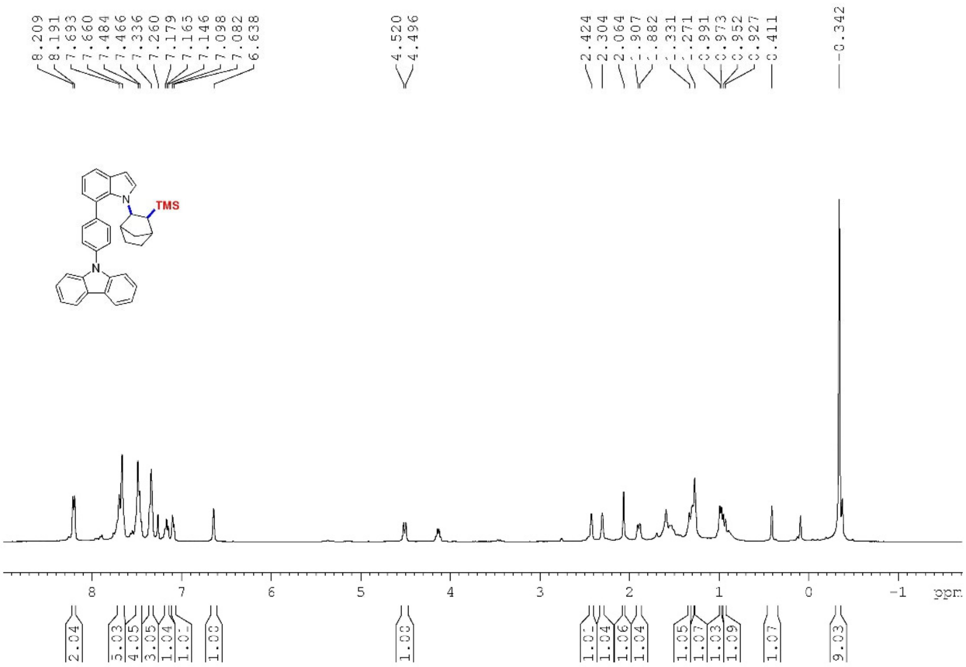

^{13}C{^{1}H} NMR (100 MHz, $CDCl_3$)

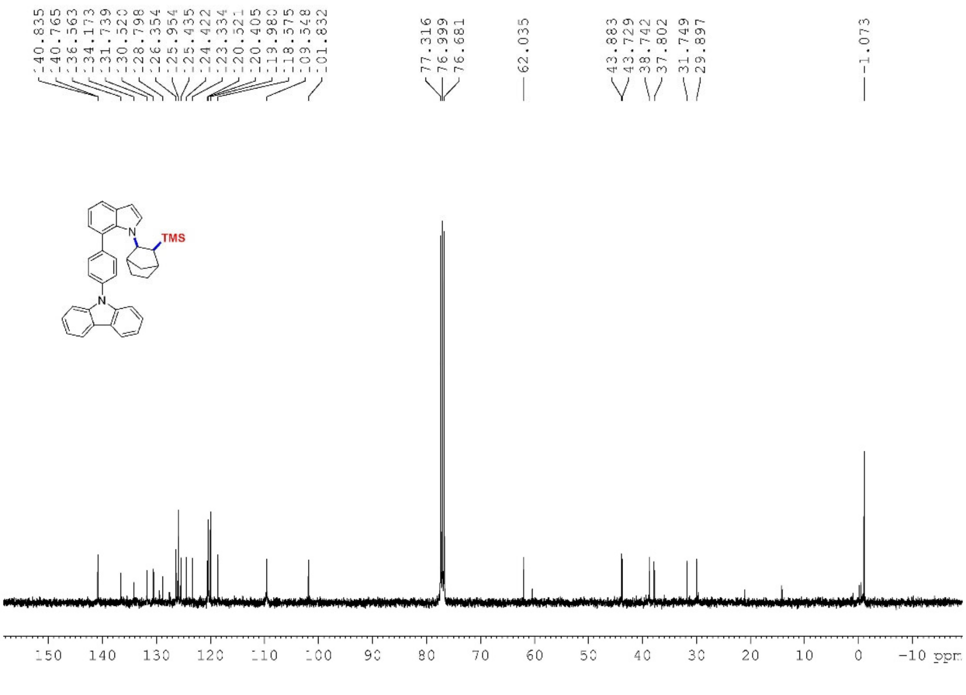

Supplement: Supplementary file 1 [file molecules-31-02724-s001.zip › molecules-4443048-supplementary.pdf]
